# Establishing mRNA and microRNA interactions driving disease heterogeneity in amyotrophic lateral sclerosis patient survival

**DOI:** 10.1093/braincomms/fcad331

**Published:** 2023-12-07

**Authors:** Rachel Waller, Joanna J Bury, Charlie Appleby-Mallinder, Matthew Wyles, George Loxley, Aditi Babel, Saleh Shekari, Mbombe Kazoka, Helen Wollff, Ammar Al-Chalabi, Paul R Heath, Pamela J Shaw, Janine Kirby

**Affiliations:** Sheffield Institute for Translational Neuroscience (SITraN), The University of Sheffield, Sheffield S10 2HQ, UK; Neuroscience Institute, The University of Sheffield, Sheffield S10 2TN, UK; Sheffield Institute for Translational Neuroscience (SITraN), The University of Sheffield, Sheffield S10 2HQ, UK; Sheffield Institute for Translational Neuroscience (SITraN), The University of Sheffield, Sheffield S10 2HQ, UK; Sheffield Institute for Translational Neuroscience (SITraN), The University of Sheffield, Sheffield S10 2HQ, UK; Sheffield Institute for Translational Neuroscience (SITraN), The University of Sheffield, Sheffield S10 2HQ, UK; Sheffield Institute for Translational Neuroscience (SITraN), The University of Sheffield, Sheffield S10 2HQ, UK; Sheffield Institute for Translational Neuroscience (SITraN), The University of Sheffield, Sheffield S10 2HQ, UK; Sheffield Institute for Translational Neuroscience (SITraN), The University of Sheffield, Sheffield S10 2HQ, UK; Sheffield Institute for Translational Neuroscience (SITraN), The University of Sheffield, Sheffield S10 2HQ, UK; Department of Basic and Clinical Neuroscience, Institute of Psychiatry Psychology and Neuroscience, King’s College London, London, SE5 9RX, UK; Department of Neurology, King’s College Hospital, London, SE5 9RS, UK; Sheffield Institute for Translational Neuroscience (SITraN), The University of Sheffield, Sheffield S10 2HQ, UK; Sheffield Institute for Translational Neuroscience (SITraN), The University of Sheffield, Sheffield S10 2HQ, UK; Neuroscience Institute, The University of Sheffield, Sheffield S10 2TN, UK; Sheffield Institute for Translational Neuroscience (SITraN), The University of Sheffield, Sheffield S10 2HQ, UK; Neuroscience Institute, The University of Sheffield, Sheffield S10 2TN, UK

**Keywords:** amyotrophic lateral sclerosis, lymphoblastoid cell line, miRNA, mRNA, survival

## Abstract

Amyotrophic lateral sclerosis is a fatal neurodegenerative disease, associated with the degeneration of both upper and lower motor neurons of the motor cortex, brainstem and spinal cord. Death in most patients results from respiratory failure within 3–4 years from symptom onset. However, due to disease heterogeneity some individuals survive only months from symptom onset while others live for several years. Identifying specific biomarkers that aid in establishing disease prognosis, particularly in terms of predicting disease progression, will help our understanding of amyotrophic lateral sclerosis pathophysiology and could be used to monitor a patient’s response to drugs and therapeutic agents. Transcriptomic profiling technologies are continually evolving, enabling us to identify key gene changes in biological processes associated with disease. MicroRNAs are small non-coding RNAs typically associated with regulating gene expression, by degrading mRNA or reducing levels of gene expression. Being able to associate gene expression changes with corresponding microRNA changes would help to distinguish a more complex biomarker signature enabling us to address key challenges associated with complex diseases such as amyotrophic lateral sclerosis. The present study aimed to investigate the transcriptomic profile (mRNA and microRNA) of lymphoblastoid cell lines from amyotrophic lateral sclerosis patients to identify key signatures that are distinguishable in those patients who suffered a short disease duration (<12 months) (*n* = 22) compared with those that had a longer disease duration (>6 years) (*n* = 20). Transcriptional profiling of microRNA–mRNA interactions from lymphoblastoid cell lines in amyotrophic lateral sclerosis patients revealed differential expression of genes involved in cell cycle, DNA damage and RNA processing in patients with longer survival from disease onset compared with those with short survival. Understanding these particular microRNA–mRNA interactions and the pathways in which they are involved may help to distinguish potential therapeutic targets that could exert neuroprotective effects to prolong the life expectancy of amyotrophic lateral sclerosis patients.

## Teaser

Waller *et al*. report alterations in cell cycle, DNA damage and RNA processing are driving disease heterogeneity in patient survival as evidenced in interacting mRNA and microRNA. Understanding these impacted biological pathways could help to distinguish potential therapeutic targets aimed at slowing the disease course of amyotrophic lateral sclerosis.

## Graphical Abstract

**Graphical Abstract:**
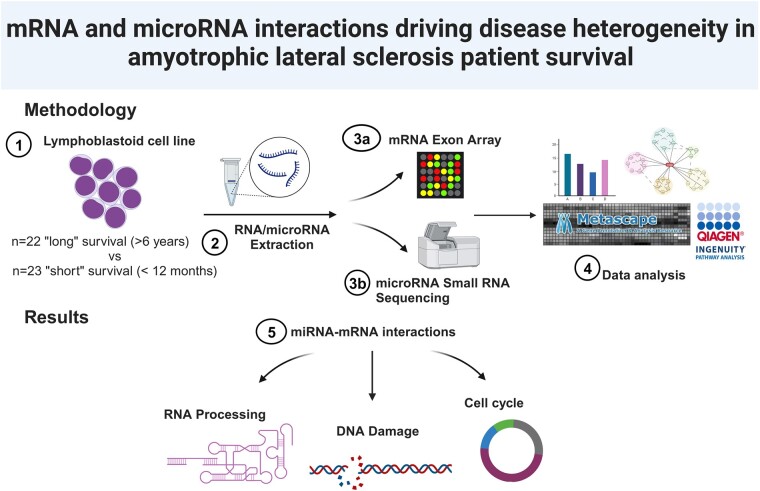

## Introduction

Amyotrophic lateral sclerosis (ALS) is a fatal neurodegenerative disease, associated with the degeneration of both upper and lower motor neurons of the motor cortex, brainstem and spinal cord. Motor neuron loss leads to progressive muscle wasting, with death in most patients resulting from respiratory failure within 3–4 years from symptom onset.^1^ ALS typically follows a variable disease trajectory with some individuals surviving only months from symptom onset while others live for several years, with ∼10% of affected individuals living >10 years.^2^

Identifying specific biomarkers to aid disease diagnosis and prognosis, particularly in terms of distinguishing ALS from disease mimics, classifying disease subtypes and predicting disease progression, has been undertaken by ourselves and others.^3-5^ Identifying biomarkers, such as neurofilament light (NfL), a known ALS pathological marker reflecting axonal degeneration may also help our understanding of ALS pathology and has also been used to monitor patient’s response to drugs and therapeutic agents.^6-8^

Transcriptomic profiling via microarray and next generation sequencing technologies are continually evolving and reducing in cost, enabling them to be used to identify key gene changes in biological processes associated with disease as well as potential biomarkers. MicroRNAs (miRNAs) are small non-coding RNAs typically associated with regulating gene expression, by degrading mRNA or reducing levels of gene expression.^9^ MiRNAs are extremely stable, resistant to RNase degradation, repeated freeze thaw cycles and changes in pH, making them ideal as potential biomarkers.^10,11^ MiRNA biomarkers examined from biological fluid and histological tissue have been associated with a range of diseases, and there is increasing evidence for their role in neurological diseases as recently reviewed.^12^ Being able to associate gene expression changes with corresponding miRNA changes would help to distinguish a more complete biomarker signature that could help to address key challenges associated with complex diseases such as ALS.

This study aims to investigate the transcriptomic profile (mRNA and miRNA) of lymphoblastoid cell lines (LCLs) from ALS patients to identify key signatures that are distinguishable in those that experienced a rapid disease course and short disease duration (<12 months) (*n* = 22) compared with those that had a more slowly progressive disease course with a longer disease duration (>6 years) (*n* = 20). LCLs have been used as models to study several neurological diseases with transcriptomic studies carried out in patients with spastic staxia,^13^ bipolar disorder,^14^ schizophrenia^15^ and Alzheimer’s Disease.^16^ In ALS, features typical of degenerating motor neurons including protein aggregation and mitochondrial dysfunction were identified from LCL.^17,18^ By identifying pathways that are attributed to a particular disease course, therapeutic strategies could be developed to promote or suppress specific gene expression changes and thereby extend survival.

## Materials and methods

### Ethics

The work was approved by the South Sheffield Research Ethics Committee (REC12/YH/0330; STH16573). Informed written consent was obtained for all patients agreeing to participate in the study.

### Study cohort

**Figure 1:**
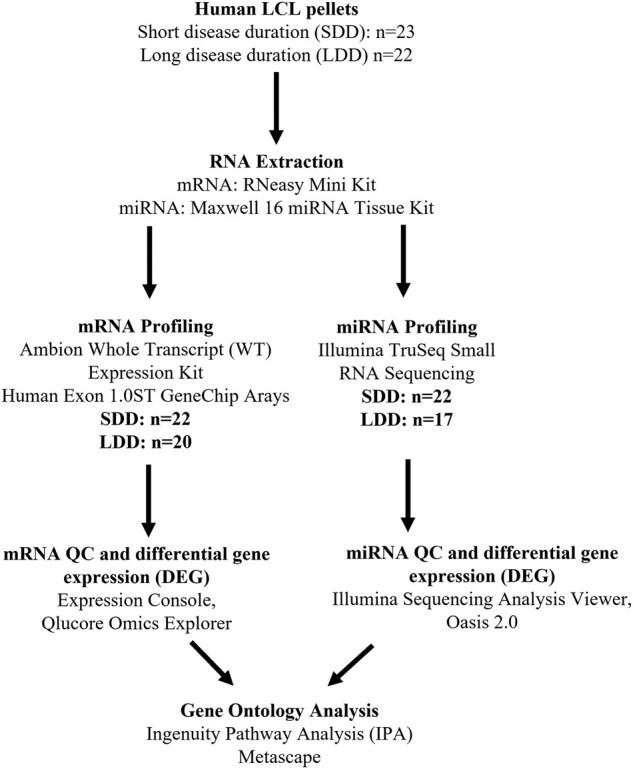
Study workflow mRNA and miRNA sample preparation, expression profiling and data analysis.

Frozen Epstein–Barr virus-transformed, immortalized lymphoblastoid cell (LCL) pellets, preserved in RNAlater™ (Sigma-Aldrich^®^, UK), were obtained from the National MNDA DNA Bank held at the Health Protection Agency’s European Collection of Cell Cultures (ECACC) (Public Health England, UK). The cohort comprised of 45 participants in total, with 23 ALS patients identified as having short disease duration (survival <12 months after symptom onset) and 22 ALS patients having a long disease duration (survival >6 years after symptom onset). See Supplementary Table 1 for full clinical details. The workflow of the study is outlined in Fig. 1.

### RNA extraction

For mRNA extraction, 75 µl LCL suspension containing ∼5 × 10^6^ cells was taken through the QIAGEN RNeasy Mini Kit protocol following the manufacturer’s instructions, with total RNA eluted in 40 μl RNase-free water. The quantity and quality of the extracted RNA were determined using a Nanodrop 1000 spectrophotometer (Thermo Fisher Scientific, UK) and a 2100 Bioanalyzer (Agilent, USA), respectively. For miRNA extraction, 100 μl LCL suspension containing ∼6.6 × 10^6^ cells was taken through the Maxwell 16 miRNA tissue kit protocol using the Maxwell 16MDX instrument following the manufacturer’s instructions, with miRNA eluted in 60 μl RNase-free water. To control for variations of miRNA extraction, each sample was spiked with 5 μl of 5 nM synthetic *Caenorhabditis elegans* miRNA 39 (Syn-cel-miR-39) (Qiagen, UK). Following extraction, it was not possible to quantify the miRNA concentration of LCL samples using the Nanodrop system, therefore, equivalent LCL volumes were used as input for the RNA extraction. Bioanalyzer Nanochips (Agilent) were used to identify the presence of miRNAs in the extracted samples (Supplementary Fig. 1). All extracted RNA/miRNA samples were stored at −80°C prior to expression profiling.

### mRNA exon array

Transcriptional profiling of peripheral LCL’s from ALS patients with short and long disease was performed using Affymetrix Human Exon 1.0ST GeneChip microarrays. In brief, ∼300 ng of extracted RNA was processed using the Ambion Whole Transcript (WT) Expression Kit (Thermo Fisher Scientific) producing fragmented biotin-labelled sense-stranded copy DNA that was hybridized onto Human Exon 1.0ST GeneChip microarrays. Post-hybridization array washing, and staining were performed in the GeneChip Fluidics station and arrays scanned using the GeneChip 3000 7G scanner.

### miRNA, small RNA sequencing

Small RNA sequencing of peripheral LCL’s from ALS patients with short and long disease was performed using the Illumina TruSeq Small RNA library preparation kit following a modified protocol as described previously.^19^ Briefly, adapter-ligated RNA was reverse transcribed to generate a cDNA library and amplified following 15 cycles of polymerase chain reaction. For cluster generation, 1000 pM of each cDNA library from 12 barcoded samples was pooled together and loaded onto 2 lanes of the flow cell at 20 pM concentration per lane. Each sample pool was loaded across two lanes of the flow cell to maximize the number of mapped reads (50 bp single read). Samples were processed using the Illumina TruSeq SBS Kit (v3) for 51 cycles of sequencing and for 7 cycles of the indexing read.

### mRNA data analysis

Individual sample .CEL files quantifying gene transcripts were uploaded into Expression Console (Thermo Fisher Scientific) and several quality control (QC) parameters were checked for each sample including poly-A RNA labelling controls, hybridization controls, positive versus negative area under the curve and signal intensity. Any outlier sample was excluded from further analysis. Qlucore Omics Explorer (Qlucore, Lund, Switzerland) was used to identify differentially expressed genes (DEGs) between successfully QC’d samples with long versus short disease duration (FC ≥ ± 1.20, *P*-value 0.05). Both Metascape (http://metascape.org)^20^ and Ingenuity Pathway Analysis (IPA) (QIAGEN Inc., https://digitalinsights.qiagen.com/IPA)^21^ were used to interrogate the data further, to identify common pathways and biological functions associated with disease duration. Additionally, IPA uses the *z*-score as both a measure of significance and a predictor of pathway activation, with a significant activated pathway *z*-score of >2, and significant inhibited pathway *z*-score of <2.^21^

### miRNA data analysis

Initial QC analysis of post-sequencing data was carried out using Illumina Sequencing Analysis Viewer to identify the Illumina Phred quality score (>Q30) and pass filter reads. Sequence data were converted from the generated image .bcl files to raw, demultiplexed sequencing data in the .fastq text file format using the Illumina developed bcl2fastq pipeline. Following QC, individual samples were uploaded into Oasis 2.0 an online small RNA-sequencing data analysis software package.^22,23^ The Illumina TruSeq 3′adapter (TGGAATTCTCGGGTGCCAAGG) sequence was removed from the.fastq file of each sample’s reads and the read size filtered (15–32 nt) with low abundance reads (<5 reads) discarded. All remaining reads were mapped to the Human genome [Oasis 2.0: GRCh38 (hg38)]. Differential miRNA expression using DESeq2 between patient samples with long disease duration and short disease duration were assessed. DESeq2 supports analyses between multiple groups containing multiple samples. IPA was used to analyse the diseases and biological functions of the differentially expressed miRNA associated with disease duration.

### Interacting mRNA and miRNA analysis

The DEG list generated in Qlucore and the differentially expressed miRNA list generated in Oasis 2.0 were uploaded into IPA and the ‘miRNA Target filter’ function was used to provide insights into the biological effects of the differentially expressed miRNAs. We focused on analysing opposing mRNA–miRNA interactions, i.e. up-regulated miRNAs targeting down-regulated mRNAs and vice versa. Of note, some identified miRNAs belong to the same family of miRNAs and have the same seed sequence and therefore are most likely to target the same genes. For completeness both differentially expressed miRNA (derived from Oasis 2.0) and the miRNA symbol (identified by IPA) are displayed in the results tables. We acknowledge that miRNA–mRNA interactions are complex and IPA algorithms use both experimentally validated miRNA–mRNA interactions from TarBase and miRecords as well as predicted miRNA–mRNA interactions from TargetScan from which we have drawn our study’s conclusions from.

### Statistical analysis

As described above, we used Qlucore Omics Explorer (Qlucore) to identify DEGs using a two-group comparison (*t*-test) and DESeq2 in OASIS 2.0 to identify differentially expressed miRNAs. A threshold level of (FC ≥ ± 1.20, *P*-value 0.05) between long versus short disease duration was set for both mRNA and miRNA expression. Both Metascape (http://metascape.org) and IPA were used to interrogate the data further. Principal components analysis was also completed in Qlucore Omics Explorer to address any confounding technical or clinical factors influencing mRNA/miRNA expression (Supplementary Fig. 2).

## Results

### Gene expression changes associated with disease duration

Following rigorous QC procedures, a final mRNA array dataset of 22 out of 23 short disease duration and 20 out of 22 long disease duration samples were taken forward for further analysis. Three outlier samples (1 short disease duration and 2 long disease duration) were removed from the initial 45 samples due to poor QC upon reviewing RNA/microarray results. All raw data CEL. files are freely available at Gene Expression Omnibus (accession number: GSE212134).

**Figure 2:**
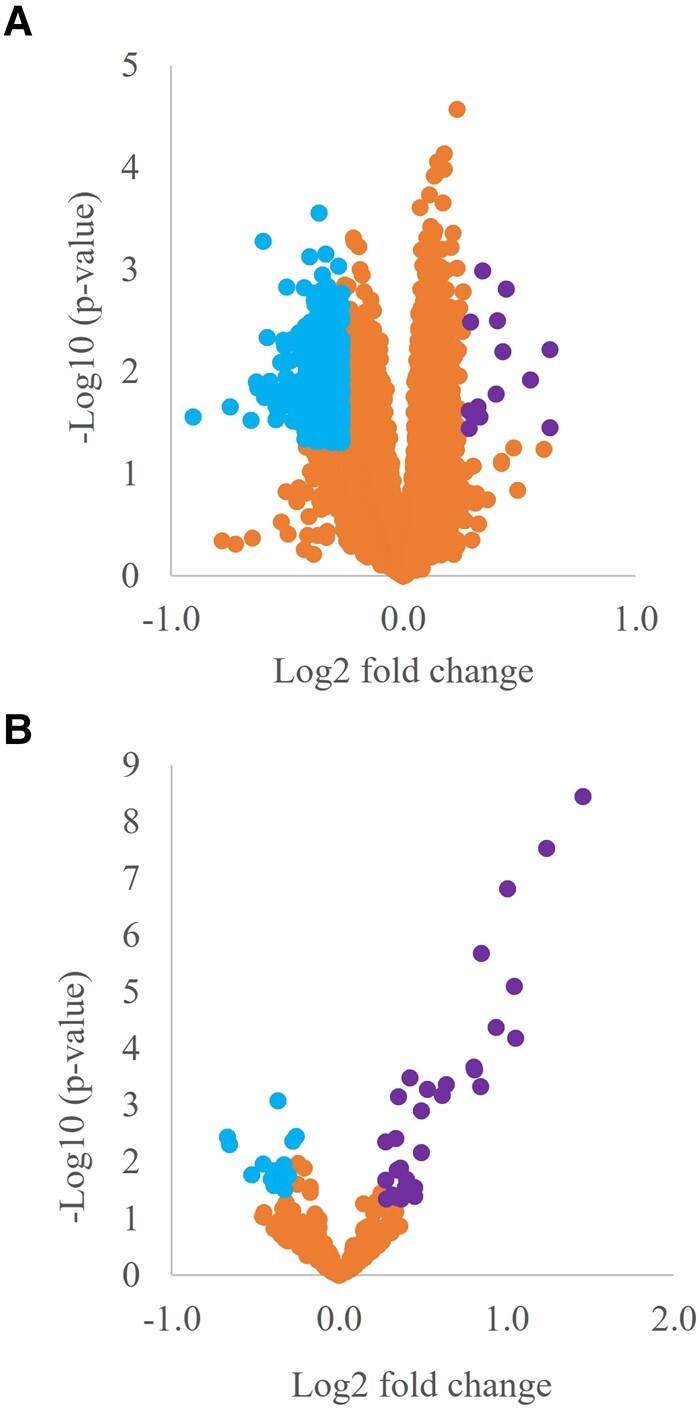
**Volcano plot representation of differential gene/miRNA expression.** The signal detection results show the fold change (fc) (log_2_fc, *x*-axis) and significance (−log_10_  *P*-value). for gene/miRNA expression in LCL’s from ‘long’ and ‘short’ disease duration. Differential gene expression (*n* = 20 long, *n* = 22 short) (**A**). Differential miRNA expression (*n* = 17 long, *n* = 22 short) (**B**). Spots to the right of the plot represent significantly up-regulated genes/miRNA, spots to the left of the plot represent significantly down-regulated genes/miRNA in long versus short disease duration.

Qlucore identified 1034 DEGs including 766 fully annotated RefSeq genes between the short and long disease duration LCLs (Supplementary Table 2). Only annotated RefSeq genes were used for downstream analyses. Overall, there was a higher number of significantly down-regulated genes in the longer disease duration samples when compared with the shorter disease duration samples (14 up-regulated, 752 down-regulated) (Fig. 2A). Multiple testing analysis revealed no significant genes.

### Dysregulation in key cellular processes and functions are associated with longer disease duration

**Figure 3:**
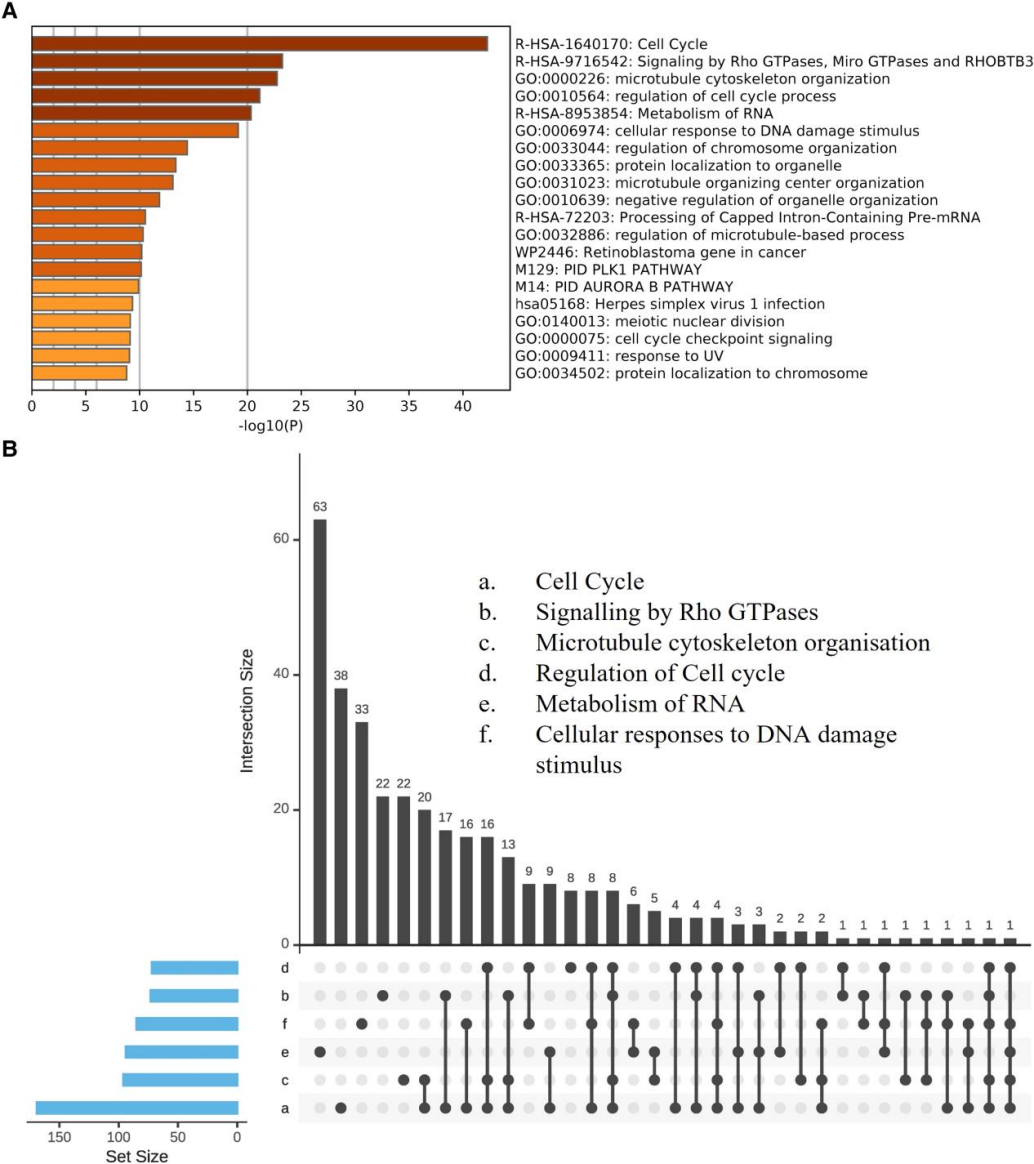
**mRNA Metascape enriched ontology clusters**. Top-20 significantly enriched clusters using the short versus long disease duration differentially expressed mRNA gene list. A significant cluster threshold is greater than −Log_10_  *P-*value = 1.3 (**A**). An upset plot comparing the number of overlapping genes expressed across the top-6 Metascape enriched ontology clusters [(i) cell cycle, (ii) signalling by Rho GTPases, (iii) microtubule cytoskeleton organization, (iv) regulation of cell cycle, (v) metabolism of RNA, (vi) cellular responses to DNA damage stimulus] (**B**). Sample size *n* = 20 long, *n* = 22 short. Key: UV, ultraviolet.

Analysis of enriched ontology clusters in Metascape, revealed clusters related to cell cycle: [cell cycle (Log *P* −42.6); regulation of cell cycle process (Log *P* −42.6); meiotic nuclear division (Log *P* −9.12) and cell cycle checkpoint signalling (Log *P* −9.10)] and cellular structure: [microtubule cytoskeleton organization (Log *P* −22.75); microtubule organizing centre organization (Log *P* −13.09); and regulation of microtubule-based process (Log *P* −10.31)], alongside clusters related to DNA damage [cellular response to DNA damage stimulus (Log *P* −19.14)] and metabolism of RNA (Log *P* −20.34; Fig. 3A and B, Supplementary Table 3). From the top-6 enriched clusters identified in Metascape, overlapping genes were found across the clusters, with most overlap between cell cycle, signalling by Rho GTPases, microtubule cytoskeleton organization and cellular responses to DNA damage stimulus. RNA metabolism showed the most distinctive gene expression profile from the top-6 enriched clusters (63/94) (Fig. 3B).

**Figure 4:**
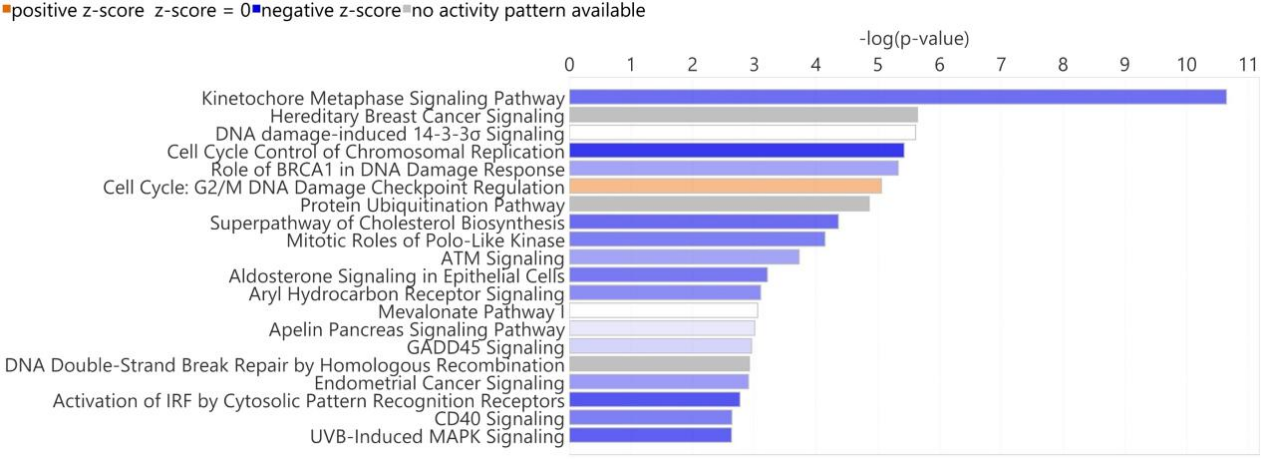
**mRNA ingenuity pathway analysis (IPA) canonical pathways analysis.** A bar chart displaying the top-20 significant diseases and biological function groups generated in IPA using the short versus long disease duration differentially expressed mRNA gene list. Blue bars—negative z-score indicating inhibition. Orange bars—positive z-score indication activation. Sample size *n* = 20 long, *n* = 22 short. Key: IRF, interferon-regulatory factor, UVB; ultraviolet B, MAPK; mitogen-activated protein kinase. This image was created with BioRender.com.

Findings were also corroborated in IPA using the canonical pathway analysis tool that identifies signalling or metabolic pathways which are significantly enriched and/or likely to be activated (positive *z*-score) or inhibited (negative *z*-score) in the dataset. Enriched inhibited pathways included those related to cell cycle; [kinetochore metaphase signalling pathway (Log *P* −10.6, *z*-score −2.40), cell cycle control of chromosomal replication (Log *P* −5.43, *z*-score −3.32), mitotic roles of polo-like kinase (Log *P* −4.15, *z*-score −2.12)], and DNA damage; [Role of BRCA1 in DNA Damage Response (Log *P* −5.33, *z*-score −1.51), G2/M DNA damage checkpoint regulation (Log *P* −5.06, *z*-score n/a), ATM Signalling (−3.73, *z*-score −1.51)] (Fig. 4; Supplementary Table 4).

**Figure 5:**
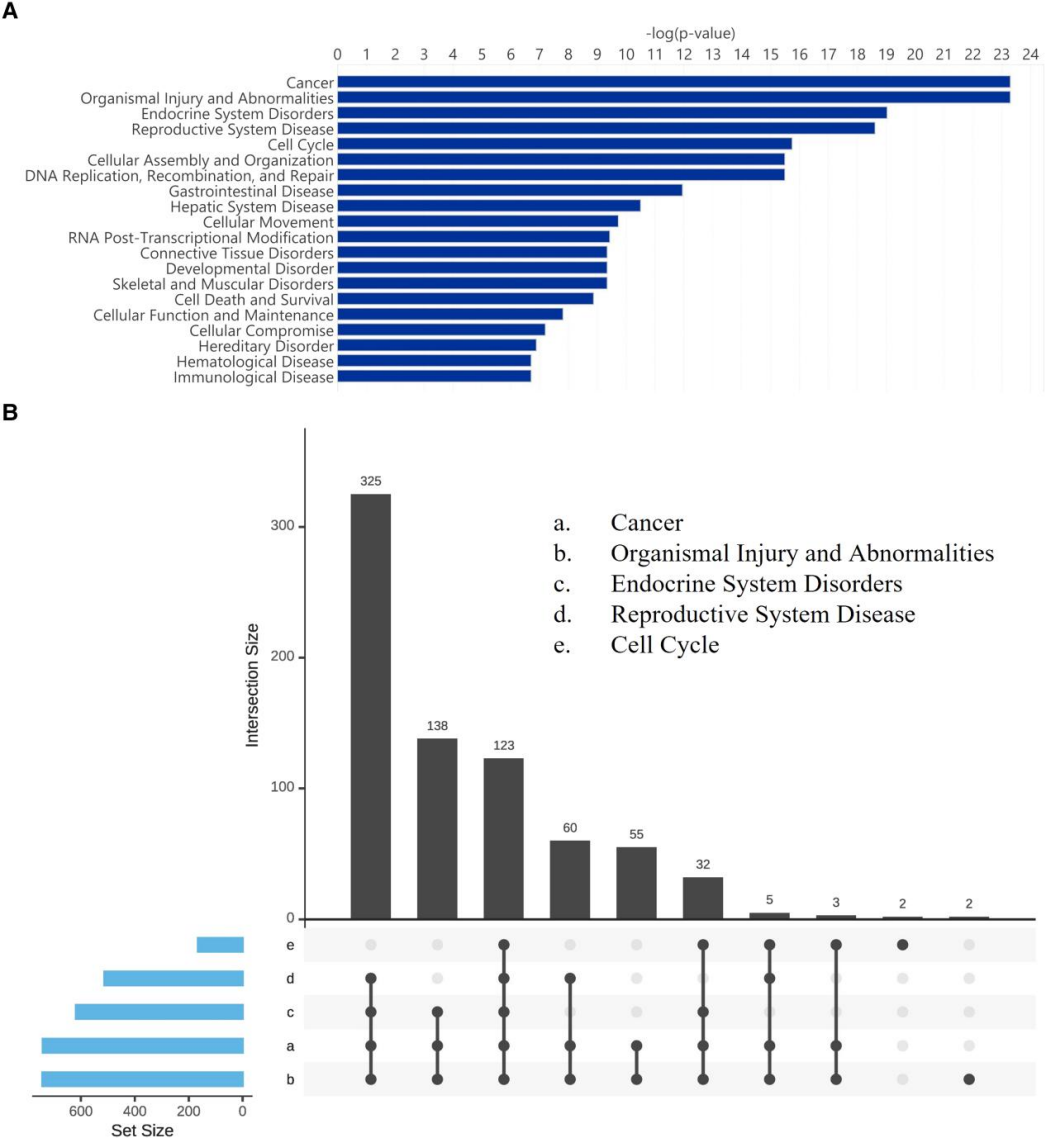
**mRNA ingenuity pathway analysis (IPA) diseases and functions analysis.** A bar chart displaying the top-20 significant diseases and biological function categories using the short versus long disease duration differentially expressed mRNA gene list (**A**). An upset plot comparing the number of overlapping genes expressed across the top-5 significant disease and biological function categories (a. Cancer, b. Organismal Injury and Abnormalities, c. Endocrine System Disorders, d. Reproductive System Disease, e. Cell Cycle) (**B**). Sample size *n* = 20 long, *n* = 22 short. This image was created with BioRender.com.

Additionally, IPA can cluster diseases and functions of gene expression changes into master categories that can be interrogated more closely (Fig. 5A). From the top five categories: cancer, organismal injury and abnormalities, endocrine system disorders, reproductive system disease and cell cycle an overlap of DEGs was identified (Fig. 5B). More specifically, corroborating findings previously identified in Metascape and IPA’s canonical pathway analysis, were changes related to cell cycle; (p 1.81E−16 to 8.2E−04), cellular structure; [cell assembly and organization (p 3.24E−16 to 8.3E−04), cellular movement (p 1.88E−10 to 1.06E−07) and cell morphology (p 2.85E−07 to 7.15E−04)]. Damage-related functions; [DNA replication recombination and repair (p 3.24E−16 to 7.52E−04), cell death and survival (p 1.35E−09 to 8.3E−04), cellular function and maintenance (p 1.54E−08 to 8.3E−04) and cellular compromise (p 6.33E−08 to 7.44E−04)] and RNA post-transcriptional modification; (p 3.72E−10 to 1.25E−08) (Supplementary Table 5).

In summary, using three different analysis tools, it is evident that in LCLs from patients with a longer disease duration, there is a decrease in expression of genes involved in the cell cycle, DNA damage and RNA metabolism. In contrast, these genes are increased in those patients with shorter survival, highlighting pathways which are already known to be implicated in ALS disease pathogenesis. These data suggest, however, that there is a variable impact and response across ALS patient cohorts, influencing patient survival in those living longer compared with those living shorter.

### miRNA changes associated with disease duration

To coincide with the samples analysed from the gene expression microarray study, following QC of sequencing data, a total of 22 out of 22 short disease duration and 17 out of 20 long disease duration samples were taken forward for further analysis. A total of 371 miRNAs were identified with 46 miRNAs differentially expressed between patients with long disease duration versus short disease duration (FC ≥ ± 1.20, *P*-value 0.05), 17 of which passed multiple testing, (*P*adj < 0.05) (Fig. 2B; Supplementary Table 6). Thirty-one of these differentially expressed miRNAs were up-regulated in those with a longer disease course, while the remaining 15 were down-regulated in patients living longer compared with those with a shorter disease duration (Table 1). All raw LCL miRNA data files are freely available at Gene Expression Omnibus (accession number: GSE212134).

**Table 1:** Differentially expressed miRNAs in LCL’s from long versus short disease duration (OASIS miRNA nomenclature)

miRNA ID	Sequence	Short disease duration (read count)	Long disease duration (read count)	Fold change (FC)	*P*-value
hsa-miR-6089	GGAGGCCGGGGUGGGGCGGGGCGG	25.55	106.14	2.74	3.60E−09
hsa-miR-4492	GGGGCUGGGCGCGCGCC	3.51	10.91	2.36	2.94E−08
hsa-miR-3960	GGCGGCGGCGGAGGCGGGGG	4.32	14.77	2.08	6.58E−05
hsa-miR-3195	CGCGCCGGGCCCGGGUU	46.36	121.50	2.07	8.10E−06
hsa-miR-6087	UGAGGCGGGGGGGCGAGC	21.65	49.55	2.01	1.52E−07
hsa-miR-3141	GAGGGCGGGUGGAGGAGGA	21.87	69.24	1.91	4.19E−05
hsa-miR-4532	CCCCGGGGAGCCCGGCG	259.20	507.53	1.80	2.08E−06
hsa-miR-4488	AGGGGGCGGGCUCCGGCG	20.45	46.27	1.79	4.64E−04
hsa-miR-3196	CGGGGCGGCAGGGGCCUC	15.82	33.54	1.75	2.38E−04
hsa-miR-4508	GCGGGGCUGGGCGCGCG	3.98	8.34	1.74	2.13E−04
hsa-miR-4497	CUCCGGGACGGCUGGGC	55.98	93.58	1.56	4.36E−04
hsa-miR-150-5p	UCUCCCAACCCUUGUACCAGUG	131.09	213.68	1.53	6.73E−04
hsa-let-7g-3p	CUGUACAGGCCACUGCCUUGC	4.59	6.85	1.44	5.22E−04
hsa-miR-3182	GCUUCUGUAGUGUAGUC	42.98	62.41	1.41	1.25E−03
hsa-miR-4449	CGUCCCGGGGCUGCGCGAGGCA	7.06	10.79	1.41	6.90E−03
hsa-miR-5100	UUCAGAUCCCAGCGGUGCCUCU	41.15	60.75	1.37	2.83E−02
hsa-miR-4485-3p	UAACGGCCGCGGUACCCUAA	2896.58	4273.97	1.36	4.00E−02
hsa-miR-7704	CGGGGUCGGCGGCGACGUG	805.72	1097.86	1.34	3.25E−04
hsa-miR-24-2-5p	UGCCUACUGAGCUGAAACACAG	13.42	18.42	1.32	2.07E−02
hsa-miR-616-5p	ACUCAAAACCCUUCAGUGACUU	4.31	6.00	1.31	2.79E−02
hsa-miR-1260b	AUCCCACCACUGCCACCAU	57.40	77.63	1.29	4.56E−02
hsa-miR-664a-3p	UAUUCAUUUAUCCCCAGCCUACA	22.64	31.14	1.29	1.28E−02
hsa-miR-4485-5p	ACCGCCUGCCCAGUGA	19.54	25.61	1.28	4.32E−02
hsa-miR-210-5p	AGCCCCUGCCCACCGCACACUG	93.53	120.80	1.28	7.13E−04
hsa-miR-505-3p	CGUCAACACUUGCUGGUUUCCU	22.42	28.20	1.27	4.23E−02
hsa-miR-3651	CAUAGCCCGGUCGCUGGUACAUGA	6.82	8.42	1.27	1.38E−02
hsa-miR-29c-3p	UAGCACCAUUUGAAAUCGGUUA	127.94	165.20	1.26	3.86E−03
hsa-miR-3912-3p	UAACGCAUAAUAUGGACAUGU	6.99	8.66	1.25	3.79E−02
hsa-miR-7641	UUGAUCUCGGAAGCUAAGC	243.72	300.66	1.21	4.50E−02
hsa-miR-671-3p	UCCGGUUCUCAGGGCUCCACC	67.52	83.18	1.21	4.38E−03
hsa-miR-4284	GGGCUCACAUCACCCCAU	704.79	862.13	1.21	2.12E−02
hsa-miR-330-3p	GCAAAGCACACGGCCUGCAGAGA	45.12	37.97	−1.20	3.52E−03
hsa-miR-181d-5p	AACAUUCAUUGUUGUCGGUGGGU	290.30	238.57	−1.21	4.32E−03
hsa-miR-4746-5p	CCGGUCCCAGGAGAACCUGCAGA	18.68	14.26	−1.23	1.74E−02
hsa-miR-3127-5p	AUCAGGGCUUGUGGAAUGGGAAG	6.56	5.00	−1.25	3.09E−02
hsa-miR-4791	UGGAUAUGAUGACUGAAA	88.36	68.87	−1.26	1.12E−02
hsa-miR-941	CACCCGGCUGUGUGCACAUGUGC	530.76	406.50	−1.29	8.33E−04
hsa-miR-501-3p	AAUGCACCCGGGCAAGGAUUCU	35.21	26.58	−1.29	1.74E−02
hsa-miR-8485	CACACACACACACACACGUAU	13.04	9.71	−1.30	1.82E−02
hsa-miR-3940-3p	CAGCCCGGAUCCCAGCCCACUU	8.35	6.34	−1.31	2.58E−02
hsa-miR-1254	AGCCUGGAAGCUGGAGCCUGCAGU	6.52	4.67	−1.31	1.44E−02
hsa-miR-1299	UUCUGGAAUUCUGUGUGAGGGA	67.91	48.98	−1.33	1.98E−02
hsa-miR-212-3p	UAACAGUCUCCAGUCACGGCC	21.39	14.78	−1.37	1.09E−02
hsa-miR-99b-5p	CACCCGUAGAACCGACCUUGCG	64.13	40.65	−1.44	1.67E−02
hsa-miR-143-3p	UGAGAUGAAGCACUGUAGCUC	866.19	473.33	−1.58	4.88E−03
hsa-miR-4326	UGUUCCUCUGUCUCCCAGAC	12.12	6.55	−1.59	3.61E−03

**Figure 6:**
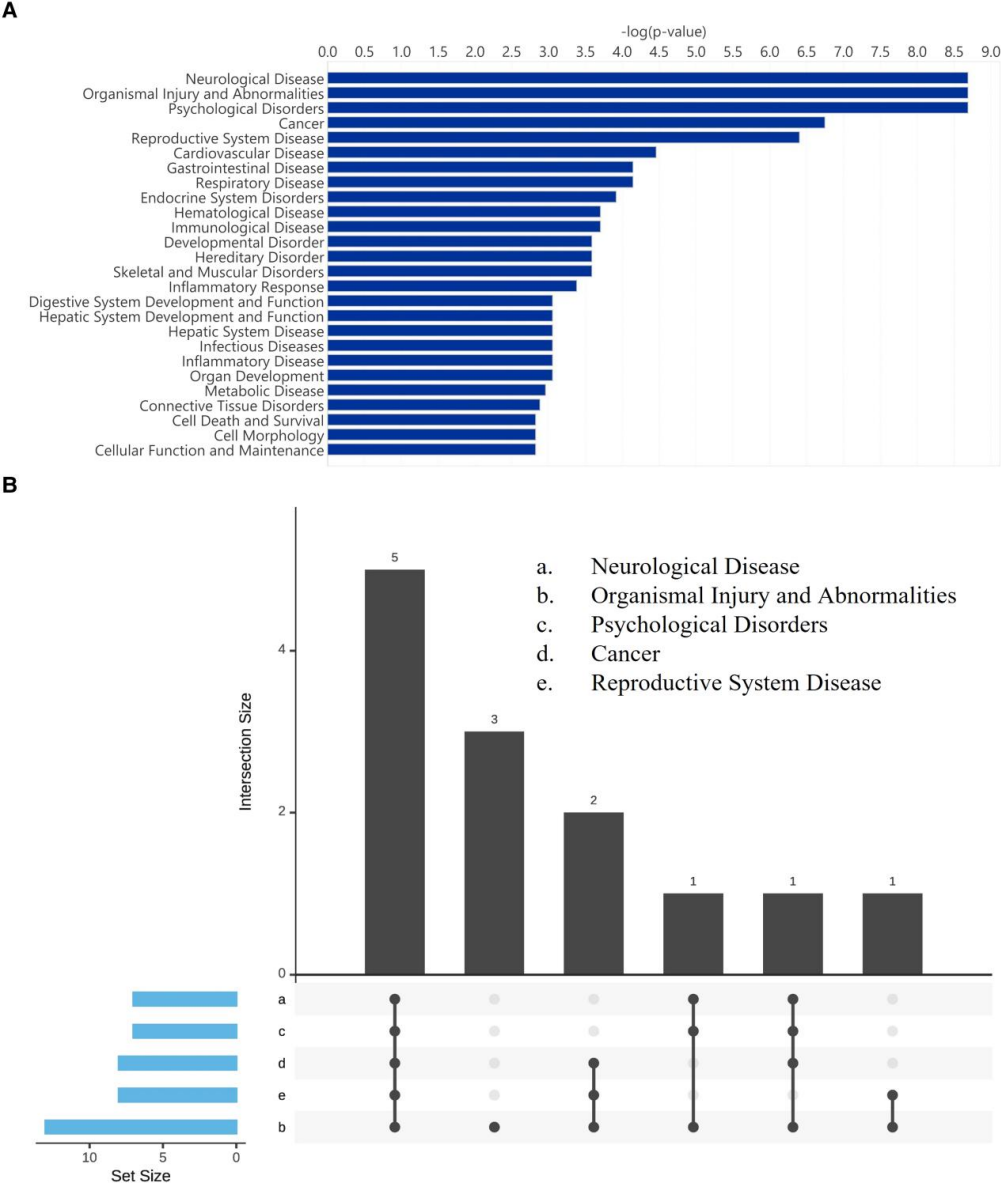
**miRNA IPA diseases and functions analysis.** A bar chart displaying the top-25 significant diseases and biological function groups using the short versus long disease duration differentially expressed miRNA gene list (**A**). An upset plot comparing the number of overlapping miRNA expressed across the top-5 significant disease and biological function categories [(i) neurological disease, (ii) organismal injury and abnormalities, (iii) psychological disorders, (iv) cancer, (v) reproductive system disease] (**B**). Sample size *n* = 17 long, *n* = 22 short. This image was created with BioRender.com.

Analysis of miRNA expression changes in IPA revealed neurological disease as the most significant disease category (Fig. 6A; Supplementary Table 7) (7 miRNAs, miR-99b-5p, miR-212-3p, miR-143-3p, miR-181d-5p, miR-616-5p, miR-29c-3p, miR-3196, p 2.05E−09 to 2.67E−02). Across the top-five disease categories including organismal injury and abnormalities, psychological disorders, cancer and reproductive system disease five of the seven differentially expressed miRNAs overlapped with the neurological disease category (Fig. 6B). Other functional categories identified in the IPA analysis sharing similar miRNAs with the neurological disease category included the cell death and survival (five miRNAs, miR-99b-5p, miR-143-3p, miR-150-5p, miR-181d-5p and miR-29c-3p) and cell morphology (miR-143-3p, p 1.5E−03) categories.

To further corroborate these findings, miRNA molecular and cellular function analysis in IPA identified cell death and survival as being the most significant group containing five enriched miRNAs, miR-143-3p, miR-181d-5p, miR-150-5p, miR-99b-5p and miR-29c-3p, p 4.68E−02 to 1.50E−03 (Supplementary Table 8). Other groups classified included cell morphology, cellular function and maintenance, cellular movement and cell cycle.

### miRNA–mRNA interactions confirm key changes in cellular processes and functions in ALS patients with a longer disease course

Interactions between miRNA and mRNA in the datasets predicted 31 miRNAs having at least 1 gene target (Table 2; Supplementary Table 9). Of the 31 miRNAs differentially expressed, 28 were up-regulated in longer disease patients compared with shorter disease patients, targeting corresponding opposing down-regulated mRNA gene targets, while three miRNAs (hsa-miR-143-3p, hsa-miR-3127-5p and hsa-miR-8485) were down-regulated in longer disease patients compared with shorter disease patients, targeting corresponding opposing up-regulated mRNA gene targets.

**Table 2:** miRNA–mRNA interactions

IPA miRNA nomenclature	OASIS miRNA nomenclature	No. gene targets
hsa-miR-29b-3p	hsa-miR-29c-3p	41
hsa-miR-505-3p	hsa-miR-505-3p	38
hsa-miR-762	hsa-miR-4492	35
Let-7a-2-3p	hsa-let-7g-3p	34
hsa-miR-616-5p	hsa-miR-616-5p	28
hsa-miR-1260a	hsa-miR-1260b	27
hsa-miR-3182	hsa-miR-3182	23
hsa-miR-1237-5p	hsa-miR-4488	21
hsa-miR-664-3p	hsa-miR-664a-3p	21
hsa-miR-7704	hsa-miR-7704	19
hsa-miR-24-1-5p	hsa-miR-24-2-5p	18
hsa-miR-3651	hsa-miR-3651	18
hsa-miR-5100	hsa-miR-5100	15
hsa-miR-3070-5p	hsa-miR-210-5p	14
hsa-miR-150-5p	hsa-miR-150-5p	11
hsa-miR-4284	hsa-miR-4284	11
hsa-miR-4485-5p	hsa-miR-4485-5p	11
hsa-miR-3912-3p	hsa-miR-3912-3p	10
hsa-miR-4532	hsa-miR-4532	10
hsa-miR-4485-3p	hsa-miR-4485-3p	7
hsa-miR-6089	hsa-miR-6089	7
hsa-miR-12197-3p	hsa-miR-4508	6
hsa-miR-3141	hsa-miR-3141	5
hsa-miR-3180-3p	hsa-miR-3196	5
hsa-miR-4449	hsa-miR-4449	5
hsa-miR-4497	hsa-miR-4497	3
hsa-miR-671-3p	hsa-miR-671-3p	3
hsa-miR-143-3p	hsa-miR-143-3p	2
hsa-miR-3127-5p	hsa-miR-3127-5p	1
hsa-miR-3960	hsa-miR-3960	1
hsa-miR-669c-3p	hsa-miR-8485	1

Both the DEG and miRNA identified in this study were supportive of each other with data generated from independent experiments. The gene target list from each of the top-5 interacting miRNAs: hsa-miR-29c-3p (41 gene targets), hsa-miR-505-3p (38 gene targets), hsa-miR-4492 (35 gene targets), hsa-let-7g-3p (34 gene targets), hsa-miR-616-5p (28 gene targets) were analysed in Metascape and enriched ontology clustering identified similar cluster groups as found in the original microarray analysis (Supplementary Fig. 3). Clusters related to cell cycle; (hsa-miR-505-3p, hsa-let-7g-3p—cell division, hsa-miR-29c-3p—negative regulation of cell cycle process, hsa-miR-616-5p—cell cycle, hsa-let-7g-3p—positive regulation of mitotic cell cycle, hsa-miR-4492—Auora B Pathway), cell structure; (hsa-miR-29c-3p—negative regulation of organelle organization, miR-505-3p, miR-616-5p—regulation of microtubule-based process/cytoskeleton organization, hsa-miR-4492—Signalling by Rho GTPases, hsa-let-7g-3p—nucleus organization), DNA repair; (miR-616-5p—DNA repair) and RNA processing (miR-4492—rRNA processing).

In summary, the majority of miRNAs extracted from LCLs in patients with a longer disease duration were up-regulated compared with patients with a shorter disease duration and involved pathways involved in cell morphology and cell death and survival. Interrogation of the predicted interactions between the top differentially expressed miRNA’s and their corresponding gene targets, cell cycle, DNA repair and RNA processing genes were identified as differentially expressed, supporting the findings demonstrated in the initial gene expression microarray study.

## Discussion

Increasing our understanding of ALS pathology is key to producing effective therapeutics and a better outcome for those living with the disease. Despite ALS having an average life expectancy of 3–4 years from symptom onset, the heterogeneity of the disease can lead to people succumbing more quickly within a year from diagnosis, while others live for several years beyond the average life expectancy. To gain better understanding of the potential causes behind the inter-individual differences in disease duration, the current study used a combined transcriptomic approach, using microarray and small RNA sequencing to investigate both mRNA and miRNA expression in LCLs from patients with longer compared with shorter disease courses. Differential expression of genes involved in the cell cycle, DNA damage and RNA processing were identified in those patients living longer compared with those with shorter disease trajectories.

We initially focused our study on the gene expression profile of ALS patients, reporting that most of the expressed genes were down-regulated in those living longer while miRNA expression appeared to be up-regulated. Interactions between the differentially expressed miRNA and mRNA supported the proposed pathways altered in the patient cohort providing essential information helping to understand the disease process.

It is well established that activation of cell cycle machinery in post-mitotic neurons with the expression of cell cycle proteins is apparent in neurons of patients with Alzheimer’s disease, stroke and ALS, leading to neuronal death.^24,25^ Cell cycle proteins expressed by neurons are needed to help regulate biological functions including the proliferation of synapses and dendritic cells. Changes in cell cycle regulators can impede signalling cascades vital for maintaining neuronal transmission and can in some cases lead to neuronal cell death.^26^ Evidence has shown that the activation of cyclin-dependent kinases (Cdks), including Cdk2, Cdk4 and Cdk6 in neurons by stress leads to cell cycle re-entry resulting in apoptosis^27^ while inhibition of Cdks induces neuroprotective properties.^28^ The reduced expression in the prolonged survivors of genes involved in the cell cycle, including the reduction in Cdk expression, specifically *Cdk*1 and *Cdk8* in the current study may suggest a possible protective mechanism contributing to a reduced pace of motor neuron injury in those patients living longer with ALS.

Cell cycle re-entry in neurodegenerative diseases is due to the contribution of several factors including environmental stresses, toxins and genetic mutations. Oxidative stress has been shown to be the main mechanism of cell cycle re-entry resulting in DNA damage in post-mitotic neurons leading to cell death associated with neurodegeneration.^25,29,30^ Enhancing DNA repair in motor neurons following injury has been shown to improve cell survival^31^ while suppressing the DNA damage response is neuroprotective.^25^ Evidently patients living longer with ALS in the current study demonstrated inhibition of DNA damage associated pathways, a potential avenue for future study.

Several lines of evidence indicate that alteration of RNA processing such as transcription, alternative splicing and miRNA biogenesis, are relevant pathogenetic factors and possible therapeutic targets for ALS.^32^ Particularly, ALS specific alterations in the *TARDBP* gene, and the TDP-43 inclusions seen in ∼97% of familial and sporadic ALS patients, have highlighted the disease-specific RNA processing changes observed in the disease.^33,34^ The present study demonstrates that those living longer have decreased expression of genes associated with RNA processing which may contribute to extended survival. This phenomenon on its own or combined with the other altered pathways identified in this study including DNA repair and cell cycle changes is worth further investigation. It is known that RNA binding is required for TDP-43 disease-associated toxicity, what is not known is whether different TDP-43 species have varying binding affinities that could cause them to be more, or less toxic. For example, p.F147L and p.F149L TDP-43 mutations that disrupt RNA binding have been shown to prevent TDP-43 mediated toxicity *in vivo.*^35^ Further investigation is warranted of those patients in the current study that survived the disease longer in relation to TDP-43 and other RNA-binding proteins.

While our study discusses transcriptomic changes associated with ALS in peripheral LCLs, if these findings can be recapitulated in neurons then this would have great impact, but caution must be taken not to over interpret the data. Nonetheless, previous work from our group has shown a dysregulation in enriched genes encoding RNA splicing proteins in both motor neuron and LCLs derived from ALS patients.^36^ Aside from ALS, many other neurodegenerative diseases characterized by the dysfunction or death of neurons in the brain, have shown transcriptional alterations associated with disease pathophysiology in peripheral tissues including fibroblasts, blood and peripheral blood mononuclear cells.^6,37,38^ Specific pathways identified from the current study have previously been linked to ALS through many different avenues, including work carried out on CNS tissue but also in the periphery. The present novel approach is the first to have identified alterations in biological pathways that are driving disease heterogeneity in patient survival supported at both the RNA and miRNA levels.

Many biomarker studies in ALS have focussed on identifying diagnostic markers, yet several studies have investigated potential prognostic markers to understand factors that accelerate or slow down the disease course.^39-44^ However, establishing a consensus in the biomarker field is difficult when various patient sample types are analysed such as from muscle, plasma, serum and CSF, with additional variation introduced through the use of different methods of analysis including quantitative polymerase chain reaction (qPCR), microarray gene expression profiling, sequencing, and mass spectrometry. Despite the study showing changes related to cell cycle, DNA damage and RNA processing following existing consensus in the ALS field additional, validation work is required. Given the lack of significant gene changes following correction for multiple testing, validating specific gene changes using alternative methodology such as qPCR in additional samples is warranted.

Recent work that investigated survival with respect to miRNA biomarkers identified high serum levels of miR-206, miR-133a and miR-151a-5p to be associated with a slower rate of clinical decline in ALS.^43^ Another study showed that increased miR-181 expression (miR-181a-5p and miR-181b-5p) in cell-free plasma was associated with a greater risk of death and when combined with measurement of NfL as a dual miRNA–protein biomarker provided a prognostic indicator for the disease course in ALS.^40^ There are four main mature products of miR-181 (miR-181a, -181b, -181c and -181d). The miR-181 identified in the present study was miR-181d-5p, which interestingly showed an opposing effect, with increased expression identified in LCLs from patients with longer survival. This highlights that care must be taken when interpreting and presenting miRNA expression levels from different sample sources and specific miRNAs regardless of their family origin.

The miRNA–mRNA interactions described in this study could lead to the identification of potential therapeutic targets through altering miRNA expression to manipulate downstream protein expression with the potential to slow down disease progression. For example, the increased expression of miR-129-5p identified in different SOD1-linked ALS models and peripheral blood cells of sporadic ALS patients compared with controls was linked to a reduction in target expression of *Hud*, at both the gene and protein levels, critical in neuronal development. Silencing miR-129-5p expression using a locked nucleic acid oligonucleotide inhibitor led to increased expression of *Hud*, and inhibition of miRNA-129-5p in the ALS SOD1^G93A^ mouse model improved the disease phenotype.^45^

## Conclusion

Transcriptional profiling of LCL’s in ALS patients revealed the differential expression of genes and miRNAs involved in the cell cycle, DNA damage and RNA processing in patients with a longer compared with a shorter disease course. Understanding these particular miRNA–mRNA interactions and the impacted biological pathways could help to distinguish potential therapeutic targets aimed at slowing the disease course of ALS.

## Supplementary Material

fcad331_Supplementary_Data

## Data Availability

The data that support the findings of this study are openly available in (Gene Expression Omnibus) at https://www.ncbi.nlm.nih.gov/geo/, accession number (GSE212134).
