# Supplementary material for: Establishing mRNA and microRNA interactions driving disease heterogeneity in amyotrophic lateral sclerosis patient survival
Source: Brain Commun. 2023 Dec 7;6(1):fcad331. doi: 10.1093/braincomms/fcad331 (PMC10754318; doi:10.1093/braincomms/fcad331)
Supplement: fcad331_Supplementary_Data [file fcad331_supplementary_data.zip › Supplementary_material.pdf]

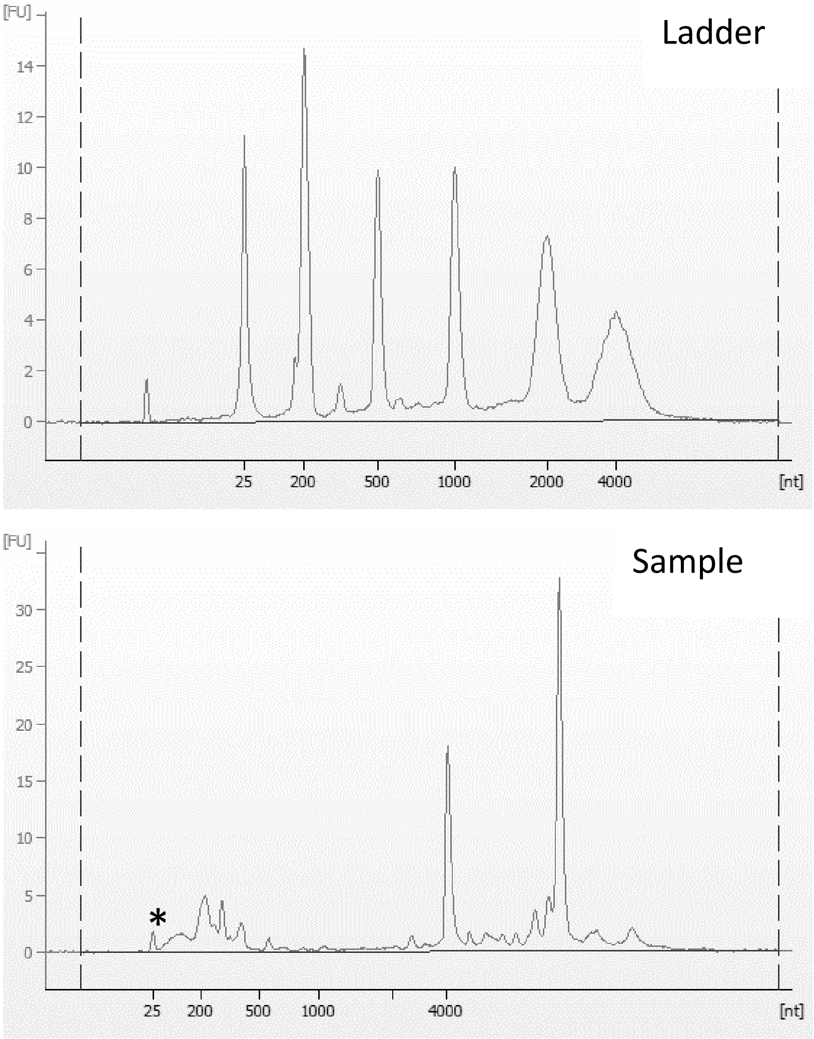

Supplementary Figure 1: miRNA content of extracted LCL sample. The presence of extracted miRNA can be seen using a RNA chip on a 2100 bioanalyser, *miRNA peak

mRNA

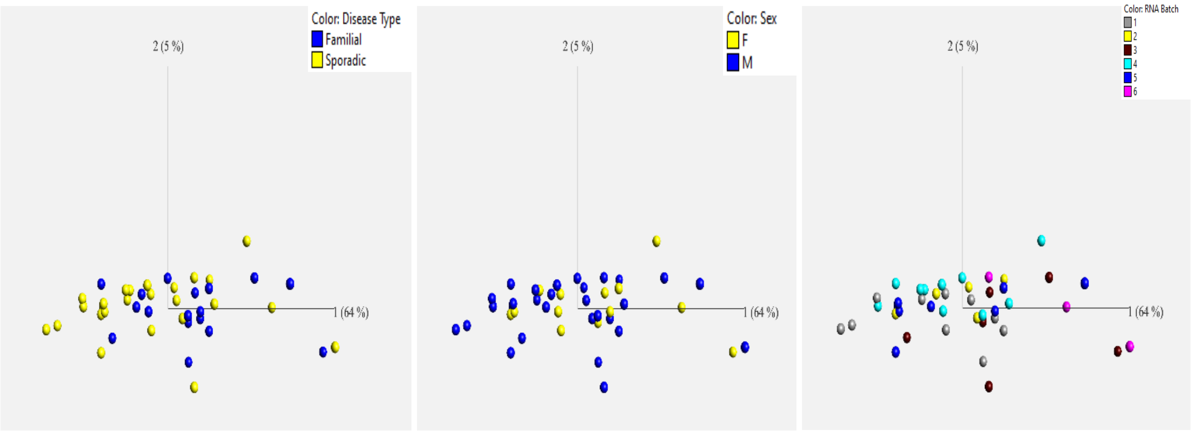

miRNA

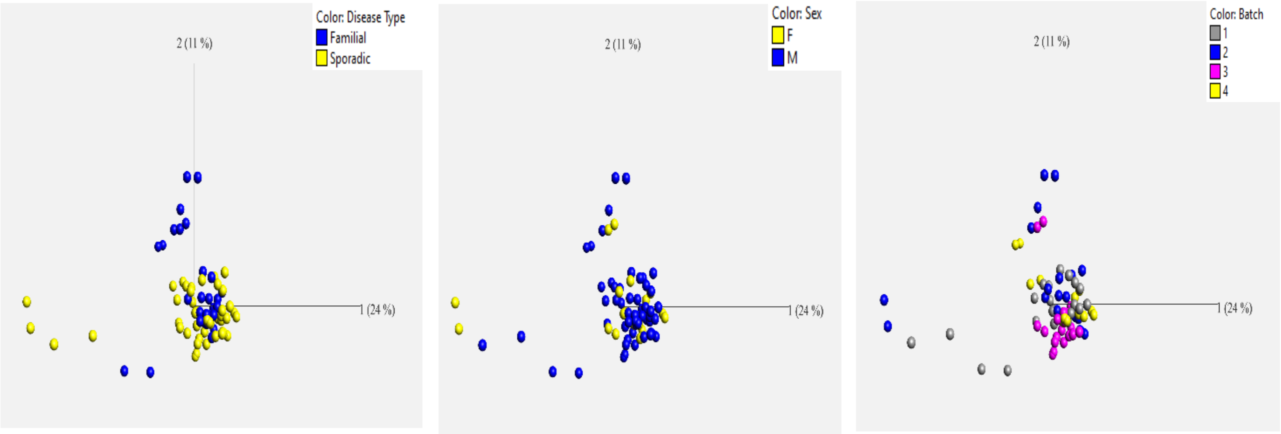

Supplementary Figure 2: PCA plots exploring confounding technical or clinical factors influencing mRNA/miRNA expression. Investigating, disease type (sporadic vs familial), sex (male vs female), and Batches of RNA.

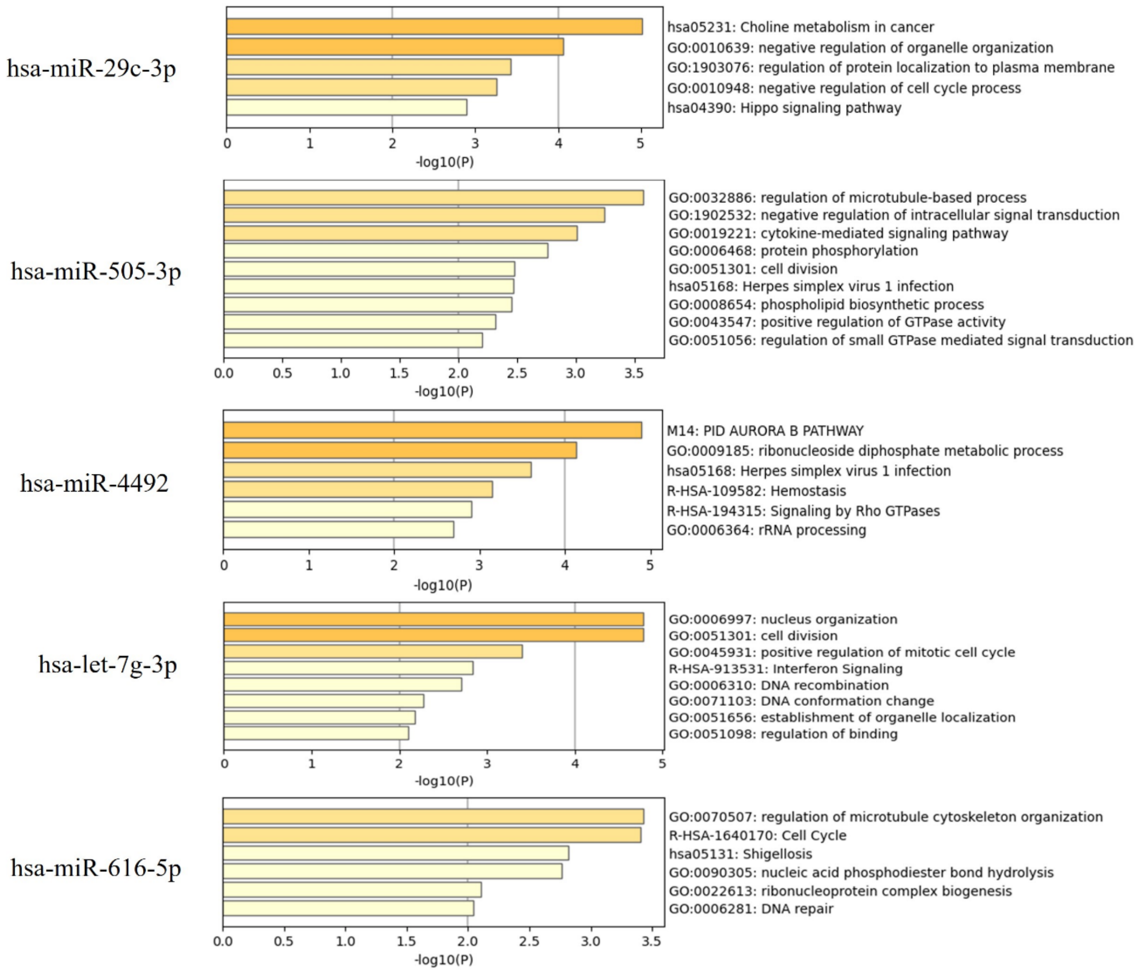

Supplementary Figure 3: Top 5 miRNA-mRNA Metascape Enriched Ontology Clusters. Top-5 miRNA-mRNA interactions and their corresponding enriched clusters. A significant threshold greater than -Log10 p value = 1.3

Supplementary Table 1 Full patient cohort clinical details.

**Short**						**Long**					
**Sample**	**Type**	**Sex**	**Age (yrs)**	**Genotype**	**Disease duration (yrs)**	**Sample**	**Type**	**Sex**	**Age (yrs)**	**Genotype**	**Disease duration (yrs)**
Short-1	Sporadic	F	63	Unknown	0.22	**Long-1**	Sporadic	M	66	Unknown	7.05
Short-2	Sporadic	M	50	*ANG*	0.17	**Long-2**	Sporadic	M	83	Unknown	6.38
Short-3	Familial	M	54	*C9ORF72*	0.54	Long-3	Familial	F	44	Unknown	6.55
Short-4	Sporadic	F	67	Unknown	0.34	**Long-4**	Sporadic	M	72	Unknown	6.01
Short-5	Sporadic	M	49	Unknown	0.17	**Long-5**	Familial	M	56	Unknown	6.01
Short-6	Familial	M	62	Unknown	0.40	**Long-6**	Sporadic	M	73	Unknown	6.54
Short-7	Sporadic	M	45	Unknown	0.47	**Long-7**	Familial	M	65	*C9ORF72*	7.26
Short-8	Sporadic	M	77	Unknown	0.29	**Long-8**	Familial	M	49	*FUS*	6.59
Short-9	Sporadic	F	77	Unknown	0.59	**Long-9**	Sporadic	F	68	Unknown	6.41
Short-10	Sporadic	M	72	*C9ORF72*	0.52	**Long-10**	Familial	M	58	*C9ORF72*	>6.00
Short-11	Familial	M	67	Unknown	0.78	**Long-11**	Sporadic	F	62	Unknown	6.67
Short-12	Sporadic	M	62	Unknown	0.55	**Long-12**	Familial	F	60	Unknown	>9.00
Short-13	Familial	M	33	Unknown	0.61	**Long-13**	Sporadic	F	47	*TARDBP*	9.62
Short-14	Sporadic	M	73	Unknown	0.66	**Long-14**	Sporadic	M	71	Unknown	7.38
Short-15	Familial	M	41	Unknown	0.88	**Long-15**	Familial	F	63	*C9ORF72*	6.92
Short-16	Sporadic	M	66	Unknown	0.98	**Long-16**	Familial	M	47	Unknown	>9.00
Short-17	Sporadic	M	64	*C9ORF72*	0.66	**Long-17**	Familial	M	62	*C9ORF72*	8.96
Short-18	Familial	F	62	Unknown	0.65	**Long-18**	Familial	M	46	*TARDBP*	>11.00
Short-19	Sporadic	M	52	Unknown	0.96	Long-19	Familial	F	41	*TARDBP*	18.34
Short-20	Familial	F	51	*C9ORF72*	0.97	Long-20	Sporadic	M	64	Unknown	9.81
Short-21	Sporadic	M	48	Unknown	0.99						
Short-22	Sporadic	F	66	Unknown	0.66						

mRNA (22 short vs 20 long), miRNA (22 short vs 17 long).
Key: F; female, M; male, yrs; years. miRNA long disease samples in bold.

Supplementary Table 3 mRNA Metascape top-20 Enriched Ontology Clusters

Description	LogP
Cell Cycle	-42.26
Signalling by Rho GTPases, Miro GTPases and RHOBTB3	-23.24
Microtubule cytoskeleton organization	-22.75
Regulation of cell cycle process	-21.14
Metabolism of RNA	-20.34
Cellular response to DNA damage stimulus	-19.14
Regulation of chromosome organization	-14.41
Protein localization to organelle	-13.34
Microtubule organising centre organisation	-13.09
Negative regulation of organelle organisation	-11.84
Processing of Capped Intron-Containing Pre-mRNA	-10.52
Regulation of microtubule-based process	-10.31
Retinoblastoma gene in cancer	-10.18
PID PLK1 PATHWAY	-10.14
PID AURORA B PATHWAY	-9.89
Herpes simplex virus 1 infection	-9.33
Meiotic nuclear division	-9.12
Cell cycle checkpoint signalling	-9.10
Response to UV	-9.05
Protein localization to chromosome	-8.79

Supplementary Table 4 mRNA IPA top-20 significant Canonical pathways

Ingenuity Canonical Pathways	-log(p-value)	z-score
Kinetochore Metaphase Signaling Pathway	10.6	-2.40
Hereditary Breast Cancer Signalling	5.64	n/a
DNA damage-induced 14-3-3σ Signaling	5.61	n/a
Cell Cycle Control of Chromosomal Replication	5.43	-3.32
Role of BRCA1 in DNA Damage Response	5.33	-1.51
Cell Cycle: G2/M DNA Damage Checkpoint Regulation	5.06	n/a
Protein Ubiquitination Pathway	4.86	n/a
Superpathway of Cholesterol Biosynthesis	4.36	-2.45
Mitotic Roles of Polo-Like Kinase	4.15	-2.12
ATM Signaling	3.73	-1.51
Aldosterone Signaling in Epithelial Cells	3.21	-2.24
Aryl Hydrocarbon Receptor Signalling	3.10	-1.90
Mevalonate Pathway I	3.05	n/a
Apelin Pancreas Signaling Pathway	3.01	-0.38
GADD45 Signaling	2.95	-0.71
DNA Double-Strand Break Repair by Homologous Recombination	2.92	n/a
Endometrial Cancer Signaling	2.91	-1.63
Activation of IRF by Cytosolic Pattern Recognition Receptors	2.77	-2.83
CD40 Signaling	2.64	-2.12

The top 20 significant canonical pathways generated in IPA using the short vs. long disease duration differentially expressed mRNA gene list. A negative z-score indicating inhibition, a positive z-score indicating activation.

Supplementary Table 5: Diseases and Biological Functions (mRNA) full dataset.

A complete list of genes, p-values and z-scores identified in the IPA generated subcategories of the disease and biological function categories; cell cycle, cellular structure; Damage related functions and RNA post-transcriptional modification.

Cell cycle

Categories	Diseases or Functions Annotation	p-value	Predicted Activation State	Activation z-score	Molecules	# Molecules
Cell Cycle	Mitosis	1.81E-16		-1.56	ANLN,ARPP19,ATR,BAZ1A,BORA,BRCA1,BRCA2,CCNB1,CCNB2,CDCA8,CDK1,CENPE,CENPF,CENPI,CKAP2,CKAP5,CSE1L,DLGAP5,DYNC1LI1,FBXO5,GNAI3,HAUS2,KIF11,KIF15,KIF2A,KIF2C,KNL1,MAD2L1,MASTL,MKI67,MLH1,NDC80,NEDD1,NOLC1,NUF2,NUSAP1,ORC2,PBRM1,PHF6,PKN2,PLK4,PPP1R12A,RBBP8,SASS6,SENP6,SGO1,SKA1,SKA3,SMC3,SPC25,STIL,TBL1XR1,TOP2A,TOP2B,TP53,TPX2,TTK,UCHL5	58
Cell Cycle,Cellular Assembly and Organization,DNA Replication, Recombination, and Repair	Segregation of chromosomes	3.24E-16		-0.46	BRCA1,CCNA2,CCNB1,CCNB2,CDK1,CENPE,CENPF,ECT2,GEN1,KIF11,KIF2C,MKI67,NCAPG,NCAPH,NDC80,NUF2,NUSAP1,PPP1R12A,SGO1,SKA1,SKA3,SMARCAD1,SMC2,SMC4,SMC6,SPC25,STAG2,TOP2A	28
Cell Cycle	Mitosis of cervical cancer cell lines	3.34E-15		-1.34	BAZ1A,BRCA2,CCNB1,CCNB2,CDCA8,CDK1,CENPE,CENPI,CKAP5,CSE1L,DLGAP5,FBXO5,GNAI3,KIF11,KNL1,MAD2L1,MASTL,NDC80,NUF2,PKN2,PLK4,SPC25,STIL,TBL1XR1,TOP2A,TOP2B,TPX2,TTK	28
Cell Cycle	M phase	7.39E-15		-1.99	AKAP9,ANLN,APC,CCNB1,CCP110,CD2AP,CDK1,CENPE,CETN2,CKAP2,CSPP1,DIAPH3,ECT2,FBXO5,GNAI3,KIF14,KIF20A,KIF20B,KIF23,MAD2L1,MASTL,MOB1A,NDC80,NUF2,NUSAP1,PIK3R4,PKN2,PRPF40A,RIOK2,SEPTIN11,SEPTIN7,SPDL1,SUSD3,TOP2A,TOP2B,TP53	36
Cell Cycle	Mitosis of tumor cell lines	1.25E-14		-1.09	ATR,BAZ1A,BRCA1,BRCA2,CCNB1,CCNB2,CDCA8,CDK1,CENPE,CENPI,CKAP5,CSE1L,DLGAP5,FBXO5,GNAI3,KIF11,KNL1,MAD2L1,MASTL,NDC80,NUF2,PKN2,PLK4,PPP1R12A,RBBP8,SPC25,STIL,TBL1XR1,TOP2A,TOP2B,TP53,TPX2,TTK	33
Cell Cycle	Cell cycle progression	1.87E-13	Decreased	-2.90	AKAP9,ANLN,APC,ARPP19,ATR,BAZ1A,BLM,BORA,BRCA1,BRCA2,CCNA2,CCNB1,CCNB2,CDCA8,CDK1,CDKN3,CENPE,CENPF,CENPI,CHUK,CKAP2,CKAP5,CSE1L,DBF4,DDX58,DLGAP5,DNAJB4,DTL,DYNC1LI1,EIF4E,FBXO5,GNAI3,GTF2H1,HAUS2,HERC5,IFI16,IFIH1,KIF11,KIF15,KIF2A,KIF2C,KNL1,MAD2L1,MAPK8,MAPK9,MASTL,MELK,METAP2,MKI67,MLH1,MMS22L,MX2,NDC80,NEDD1,NOLC1,NUF2,NUSAP1,ORC2,PBRM1,PHF6,PKN2,PLK4,PNPT1,PPP1R12A,RBBP8,RRM1,SAMHD1,SASS6,SENP6,SGO1,SKA1,SKA3,SLC38A9,SMC3,SPC25,STIL,TBL1XR1,TFCP2,TOP2A,TOP2B,TP53,TPX2,TRIM21,TSG101,TTK,UBE2D3,UCHL5,USP1,WDR12,WRN	90
Cell Cycle	Arrest in mitosis	8.93E-13			BAZ1A,CCNB1,CCNB2,CENPE,CENPI,CKAP2,CKAP5,CSE1L,FBXO5,KIF11,KNL1,MAD2L1,NDC80,NUF2,PLK4,SGO1,SPC25,TBL1XR1,TP53,TPX2,TTK	21
Cell Cycle	Arrest in mitosis of cervical cancer cell lines	2.95E-12			BAZ1A,CCNB1,CCNB2,CENPE,CENPI,CKAP5,CSE1L,FBXO5,KIF11,KNL1,MAD2L1,NDC80,NUF2,PLK4,SPC25,TBL1XR1,TPX2,TTK	18
Cell Cycle	Arrest in mitosis of tumor cell lines	8.21E-12			BAZ1A,CCNB1,CCNB2,CENPE,CENPI,CKAP5,CSE1L,FBXO5,KIF11,KNL1,MAD2L1,NDC80,NUF2,PLK4,SPC25,TBL1XR1,TP53,TPX2,TTK	19
Cell Cycle	M phase of tumor cell lines	3.11E-11	Decreased	-2.23	AKAP9,ANLN,CD2AP,DIAPH3,ECT2,FBXO5,GNAI3,KIF14,KIF20A,KIF20B,KIF23,MAD2L1,MASTL,MOB1A,NDC80,NUF2,PKN2,SEPTIN11,SEPTIN7,SPDL1,SUSD3,TOP2A,TOP2B,TP53	24
Cell Cycle	M phase of cervical cancer cell lines	1.42E-10	Decreased	-2.16	AKAP9,ANLN,CD2AP,DIAPH3,ECT2,GNAI3,KIF14,KIF20A,KIF20B,KIF23,MAD2L1,MASTL,NDC80,NUF2,PKN2,SEPTIN11,SEPTIN7,SPDL1,TOP2A,TOP2B	20
Cell Cycle,Cellular Movement	Cytokinesis	1.88E-10	Decreased	-2.67	AKAP9,ANLN,APC,CCNB1,CCP110,CD2AP,CETN2,CKAP2,CSPP1,DIAPH3,ECT2,GNAI3,KIF14,KIF20A,KIF20B,KIF23,MASTL,NUSAP1,PIK3R4,PKN2,PRPF40A,SEPTIN11,SEPTIN7,TOP2A,TP53	25
Cell Cycle	Cycling of centrosome	3.72E-10	Decreased	-2.40	AKAP9,BRCA2,CCP110,CDK1,CEP135,CETN2,CETN3,CHMP2B,CKAP5,GEN1,HAUS2,NDC80,NEDD1,PLK4,POC1B,PPP1R12A,SASS6,STIL,TP53,TTK	20
Cell Cycle	G2/M phase transition	4.85E-09			AKAP9,ALMS1,ARPP19,ATR,BLM,BORA,CCNA2,CCNB1,CCNB2,CCP110,CDK1,CEP135,CEP290,CETN2,CKAP5,HAUS2,MAD2L1,MAPRE1,MASTL,MELK,NEDD1,PLK4,PPP1R12A,TP53	24
Cell Cycle	Interphase	5.21E-08	Decreased	-2.36	AASDHPPT,AKAP9,ALMS1,APC,ARPP19,ATR,BLM,BORA,BRCA1,CCDC88A,CCNA2,CCNB1,CCNB2,CCNE2,CCP110,CDCA2,CDK1,CDKN3,CEP135,CEP290,CETN2,CKAP5,CKS2,COP1,CSE1L,CUL5,DBF4,DTL,E2F5,EIF2AK2,EIF4E,FBXO5,GEN1,HAUS2,KIF11,LMNB1,MAD2L1,MAPK8,MAPRE1,MASTL,MCM8,MELK,MLH1,MMS22L,MSH2,MTBP,MX2,NEDD1,ORC2,ORC3,ORC5,PLK4,PLSCR1,PNPT1,POLA1,PPP1R12A,PRIM2,RBBP8,REV3L,SP1,SRSF3,SUSD3,TOP2A,TP53,TRIM21,USP37	66
Cell Cycle,Cellular Movement	Cytokinesis of cervical cancer cell lines	6.50E-08	Decreased	-2.16	AKAP9,ANLN,CD2AP,DIAPH3,ECT2,GNAI3,KIF14,KIF20A,KIF20B,KIF23,MASTL,PKN2,SEPTIN11,SEPTIN7	14
Cell Cycle,Cellular Movement	Cytokinesis of tumor cell lines	1.06E-07	Decreased	-2.38	AKAP9,ANLN,CD2AP,DIAPH3,ECT2,GNAI3,KIF14,KIF20A,KIF20B,KIF23,MASTL,PKN2,SEPTIN11,SEPTIN7,TOP2A	15
Cell Cycle,Cellular Assembly and Organization,DNA Replication, Recombination, and Repair	Duplication of centriole	2.15E-07		-1.94	AKAP9,CCP110,CEP135,CETN2,NEDD1,PLK4,SASS6,STIL	8
Cell Cycle,Cell Morphology	Formation of spindle apparatus	4.15E-07		1.38	BRCA1,CKAP2,CKAP5,DYNC1LI1,HAUS2,KIF11,KIF2A,KIF2C,MAD2L1,NEDD1,NUF2,SASS6,SENP6,TPX2	14
Cell Cycle	G2 phase	4.85E-07	Decreased	-2.78	AKAP9,ALMS1,ARPP19,ATR,BLM,BORA,BRCA1,CCDC88A,CCNA2,CCNB1,CCNB2,CCP110,CDK1,CEP135,CEP290,CETN2,CKAP5,CSE1L,DTL,E2F5,HAUS2,KIF11,MAD2L1,MAPRE1,MASTL,MELK,MLH1,MMS22L,MSH2,NEDD1,PLK4,PPP1R12A,RBBP8,TOP2A,TP53	35
Cell Cycle	G2/M phase	7.48E-07	Decreased	-2.60	AKAP9,ALMS1,ARPP19,ATR,BLM,BORA,BRCA1,CCDC88A,CCNA2,CCNB1,CCNB2,CCP110,CDK1,CEP135,CEP290,CETN2,CKAP5,CSE1L,DTL,HAUS2,MAD2L1,MAPRE1,MASTL,MELK,MLH1,MSH2,NEDD1,PLK4,PPP1R12A,RBBP8,TP53	31
Cell Cycle,Cell Morphology	Formation of mitotic spindle	1.15E-06		1.40	BRCA1,CKAP2,CKAP5,DYNC1LI1,HAUS2,KIF11,KIF2A,KIF2C,NEDD1,NUF2,SASS6,SENP6,TPX2	13
Cell Cycle	Delay in mitosis	1.57E-06			ATR,BRCA1,CCNB1,CDK1,CENPE,DLGAP5,MAD2L1,TOP2A,TOP2B	9
Cell Cycle	Cell cycle progression of cervical cancer cell lines	1.73E-06		-1.00	ATR,BRCA2,CCNA2,CDK1,CSE1L,MAD2L1,MMS22L,PHF6,SAMHD1,SMC3,UCHL5	11
Cell Cycle,Cellular Assembly and Organization,DNA Replication, Recombination, and Repair	Replication of centriole	2.07E-06		-1.94	AKAP9,CCP110,CEP135,CETN2,NEDD1,PLK4,POC1B,SASS6,STIL	9

Supplementary Table 5: Diseases and Biological Functions (mRNA) full dataset.

A complete list of genes, p-values and z-scores identified in the IPA generated subcategories of the disease and biological function categories; cell cycle, cellular structure; Damage related functions and RNA post-transcriptional modification.

Cellular Assembly and Organisation

Categories	Diseases or Functions Annotation	p-value	Predicted Activation State	Activation z-score	Molecules	# Molecules
Cell Cycle,Cellular Assembly and Organization,DNA Replication, Recombination, and Repair	Segregation of chromosomes	3.24E-16		-0.46	BRCA1,CCNA2,CCNB1,CCNB2,CDK1,CENPE,CENPF,ECT2,GEN1,KIF11,KIF2C,MKI67,NCAPG,NCAPH,NDC80,NUF2,NUSAP1,PPP1R12A,SGO1,SKA1,SKA3,SMARCAD1,SMC2,SMC4,SMC6,SPC25,STAG2,TOP2A	28
Cellular Assembly and Organization,DNA Replication, Recombination, and Repair	Alignment of chromosomes	8.41E-10		-1.91	CCNA2,CENPE,DLGAP5,KIF14,KIF18A,KIF20A,KIF2C,NCAPG,SGO1,SMC4,TTK	11
Cellular Assembly and Organization,Cellular Function and Maintenance	Organization of cytoplasm	1.54E-08	Decreased	-2.32	ABCD3,ACTN1,AKAP9,AKTIP,APC,ASPM,ATL3,BLZF1,BORA,BTK,CARMIL1,CCDC88A,CCNB1,CD2AP,CDK1,CENPE,CEP20,CEP290,CEP350,CETN2,CHMP2B,CHUK,CKAP5,DAAM1,DIAPH2,DIAPH3,DLG1,DLGAP5,DNAJC13,DOCK5,DOCK7,ECT2,EIF4E,GABPA,GAS2L3,GCC2,GPR65,HMMR,HOOK1,IFT74,KIAA0586,KIF11,KIF18A,KIF20B,KIF2A,KIF2C,KIF3A,LARP4,LIMA1,LYSMD3,MAD2L1,MAPRE1,MKKS,MTBP,NDC80,NEDD1,OPA1,PIBF1,PIK3R1,PIP5K1A,PLK4,POC1B,PRKACB,PRPF40A,RAP2A,ROCK1,SASS6,SCLT1,SEPTIN7,SLC9A6,SPC25,STIL,STXBP3,TBCE,TGM2,TMEM135,TOR1AIP2,TP53,TPX2,TTK,ZFP69B,ZMYM4	82
Cellular Assembly and Organization	Formation of nucleus	1.18E-07		0.43	ATR,BLM,BRCA1,CCP110,CDK1,GEN1,KIF18A,LMNB1,NOLC1,NUP107,NUP153,RAP1GDS1,TFCP2,TP53	14
Cell Cycle,Cellular Assembly and Organization,DNA Replication, Recombination, and Repair	Duplication of centriole	2.15E-07		-1.94	AKAP9,CCP110,CEP135,CETN2,NEDD1,PLK4,SASS6,STIL	8
Cellular Assembly and Organization,Cellular Function and Maintenance	Organization of cytoskeleton	2.36E-07	Decreased	-2.32	ACTN1,AKAP9,APC,ASPM,BORA,BTK,CARMIL1,CCDC88A,CCNB1,CD2AP,CDK1,CENPE,CEP20,CEP290,CEP350,CETN2,CHUK,CKAP5,DAAM1,DIAPH2,DIAPH3,DLG1,DLGAP5,DOCK5,DOCK7,ECT2,EIF4E,GAS2L3,GCC2,GPR65,HMMR,IFT74,KIAA0586,KIF11,KIF18A,KIF20B,KIF2A,KIF2C,KIF3A,LARP4,LIMA1,MAD2L1,MAPRE1,MKKS,MTBP,NDC80,NEDD1,PIBF1,PIK3R1,PIP5K1A,PLK4,POC1B,PRKACB,PRPF40A,RAP2A,ROCK1,SASS6,SCLT1,SEPTIN7,SLC9A6,SPC25,STIL,STXBP3,TBCE,TGM2,TMEM135,TP53,TPX2,TTK,ZMYM4	70
Cellular Assembly and Organization,Cellular Function and Maintenance	Organization of mitotic spindle	2.70E-07			ASPM,BORA,CCNB1,CENPE,CKAP5,KIF11,KIF2A,NDC80,SPC25,STIL,TBCE,TPX2,TTK	13
Cellular Assembly and Organization,DNA Replication, Recombination, and Repair	Chromosomal congression of chromosomes	1.54E-06	Decreased	-2.24	CENPE,KIF14,KIF18A,KIF2C,NDC80,SGO2	6
Cell Cycle,Cellular Assembly and Organization,DNA Replication, Recombination, and Repair	Replication of centriole	2.07E-06		-1.94	AKAP9,CCP110,CEP135,CETN2,NEDD1,PLK4,POC1B,SASS6,STIL	9
Cell Cycle,Cellular Assembly and Organization,Cellular Function and Maintenance,DNA Replication, Recombination, and Repair	Duplication of centrosome	5.66E-06			BRCA2,CCP110,CHMP2B,GEN1,NDC80,SASS6,STIL,TP53,TTK	9
Cellular Assembly and Organization,Cellular Function and Maintenance	Microtubule dynamics	6.32E-06	Decreased	-2.37	AKAP9,APC,BTK,CARMIL1,CCDC88A,CD2AP,CDK1,CEP20,CEP290,CEP350,CETN2,CHUK,CKAP5,DAAM1,DIAPH2,DIAPH3,DLGAP5,DOCK5,DOCK7,ECT2,EIF4E,GAS2L3,GCC2,HMMR,IFT74,KIAA0586,KIF11,KIF18A,KIF20B,KIF2A,KIF2C,KIF3A,LIMA1,MAD2L1,MAPRE1,MKKS,MTBP,NEDD1,PIBF1,PIK3R1,PLK4,POC1B,PRKACB,RAP2A,ROCK1,SASS6,SCLT1,SEPTIN7,SLC9A6,STXBP3,TBCE,TMEM135,TP53,TPX2	54
Cell Morphology,Cellular Assembly and Organization	Morphology of nucleus	1.36E-05			APC,BRCA1,CENPE,GEN1,HAT1,KIF18A,LMNB1,MAPRE1,NCAPG,NOLC1,POT1,RAP1GDS1,SEPTIN7,SMC2	14
Cell Cycle,Cell Morphology,Cellular Assembly and Organization,DNA Replication, Recombination, and Repair	Morphology of chromosomes	1.57E-05			APC,CENPE,GEN1,HAT1,KIF18A,MAPRE1,NCAPG,SMC2	8
Cellular Assembly and Organization	Chromosomal congression of metaphase plate	3.63E-05			CENPE,CENPF,CENPQ,NDC80,SEH1L	5
Cellular Assembly and Organization	Attachment of kinetochores	3.64E-05			CDCA8,ECT2,KNL1,NDC80,NUF2,SGO1	6
Cellular Assembly and Organization	Tension of kinetochores	4.50E-05			CENPE,DLGAP5,NUF2	3
Cellular Assembly and Organization,Cellular Function and Maintenance	Organization of nuclear pores	4.50E-05			NUP133,SEH1L,TPR	3
Cellular Assembly and Organization	Quantity of chromosome components	5.73E-05			BRCA2,MAD2L1,MMS22L,SGO1,TP53	5
Cell Cycle,Cellular Assembly and Organization,DNA Replication, Recombination, and Repair	Segregation of sister chromatids	6.86E-05			CCNA2,NDC80,NUSAP1,PPP1R12A,SMC4,STAG2	6
Cellular Assembly and Organization,DNA Replication, Recombination, and Repair	Formation of nuclear foci	7.94E-05	Increased	2.20	BLM,BRCA1,BRCA2,CCNA2,CCNB1,CDK1,LMNB1,MMS22L,MSH2,POT1,RBBP8,REV3L,TP53,WRN	14
Cellular Assembly and Organization,DNA Replication, Recombination, and Repair	Alignment of sister chromatids	1.75E-04			NDC80,TOP2A,TOP2B	3
Cell Morphology,Cellular Assembly and Organization,Cellular Function and Maintenance	Formation of cilia	2.51E-04		-0.73	AKAP9,CCDC88A,CEP20,CEP290,CETN2,IFT74,KIAA0586,KIF2C,KIF3A,LIMA1,MKKS,PIBF1,PLK4,POC1B,PRKACB,SCLT1,TMEM135	17
Cellular Assembly and Organization,DNA Replication, Recombination, and Repair	Quantity of chromosomes	3.29E-04		1.00	CENPE,CENPF,KIF2C,MAD2L1,TP53	5
Cellular Assembly and Organization	Attachment of spindle fibers	4.31E-04			KNL1,NDC80,NUF2,SGO1	4
Cell Morphology,Cellular Assembly and Organization	Morphology of cytoskeleton	4.71E-04			CCP110,CKAP5,KIF11,KIF20A,KIF2C,NEDD1,TP53,TPX2,TRIP11	9
Cellular Assembly and Organization	Formation of micronuclei	5.61E-04			BLM,BRCA1,CCP110,GEN1,KIF18A	5
Cellular Assembly and Organization,Cellular Function and Maintenance	Density of microtubules	8.30E-04			APC,CKAP5,KIF2C	3

Supplementary Table 5: Diseases and Biological Functions (mRNA) full dataset.

A complete list of genes, p-values and z-scores identified in the IPA generated subcategories of the disease and biological function categories; cell cycle, cellular structure; Damage related functions and RNA post-transcriptional modification.

Cellular movement

Categories	Diseases or Functions Annotation	p-value	Predicted Activation State	Activation z-score	Molecules	# Molecules
Cell Cycle,Cellular Movement	Cytokinesis	1.88E-10	Decreased	-2.67	AKAP9,ANLN,APC,CCNB1,CCP110,CD2AP,CETN2,CKAP2,CSPP1,DIAPH3,ECT2,GNAI3,KIF14,KIF20A,KIF20B,KIF23,MASTL,NUSAP1,PIK3R4,PKN2,PRPF40A,SEPTIN11,SEPTIN7,TOP2A,TP53	25
Cell Cycle,Cellular Movement	Cytokinesis of cervical cancer cell lines	6.50E-08	Decreased	-2.16	AKAP9,ANLN,CD2AP,DIAPH3,ECT2,GNAI3,KIF14,KIF20A,KIF20B,KIF23,MASTL,PKN2,SEPTIN11,SEPTIN7	14
Cell Cycle,Cellular Movement	Cytokinesis of tumor cell lines	1.06E-07	Decreased	-2.38	AKAP9,ANLN,CD2AP,DIAPH3,ECT2,GNAI3,KIF14,KIF20A,KIF20B,KIF23,MASTL,PKN2,SEPTIN11,SEPTIN7,TOP2A	15

Supplementary Table 5: Diseases and Biological Functions (mRNA) full dataset.

A complete list of genes, p-values and z-scores identified in the IPA generated subcategories of the disease and biological function categories; cell cycle, cellular structure; Damage related functions and RNA post-transcriptional modification.

Cell morphology

Categories	Diseases or Functions Annotation	p-value	Predicted Activation State	Activation z-score	Molecules	# Molecules
Cell Morphology,Cellular Compromise	Multinucleation of cells	2.85E-07		0.98	BRCA2,DIAPH3,ECT2,GEN1,KIF14,KIF23,MAD2L1,PPP1R12A,SEPTIN11,SEPTIN7,TP53	11
Cell Cycle,Cell Morphology	Formation of spindle apparatus	4.15E-07		1.38	BRCA1,CKAP2,CKAP5,DYNC1LI1,HAUS2,KIF11,KIF2A,KIF2C,MAD2L1,NEDD1,NUF2,SASS6,SENP6,TPX2	14
Cell Cycle,Cell Morphology	Formation of mitotic spindle	1.15E-06		1.40	BRCA1,CKAP2,CKAP5,DYNC1LI1,HAUS2,KIF11,KIF2A,KIF2C,NEDD1,NUF2,SASS6,SENP6,TPX2	13
Cell Morphology,Cellular Compromise	Multinucleation of tumor cell lines	2.07E-06		1.02	DIAPH3,ECT2,GEN1,KIF14,KIF23,PPP1R12A,SEPTIN11,SEPTIN7,TP53	9
Cell Morphology,Cellular Compromise	Multinucleation of cervical cancer cell lines	3.65E-06		1.25	DIAPH3,GEN1,KIF14,KIF23,SEPTIN11,SEPTIN7,TP53	7
Cell Morphology,Cellular Compromise	Binucleation of cervical cancer cell lines	7.75E-06		0.00	DIAPH3,SEPTIN11,SEPTIN7,TP53	4
Cell Morphology,Cellular Compromise	Binucleation of tumor cell lines	1.23E-05		0.37	DIAPH3,ECT2,SEPTIN11,SEPTIN7,TP53	5
Cell Morphology,Cellular Assembly and Organization	Morphology of nucleus	1.36E-05			APC,BRCA1,CENPE,GEN1,HAT1,KIF18A,LMNB1,MAPRE1,NCAPG,NOLC1,POT1,RAP1GDS1,SEPTIN7,SMC2	14
Cell Cycle,Cell Morphology,Cellular Assembly and Organization,DNA Replication, Recombination, and Repair	Morphology of chromosomes	1.57E-05			APC,CENPE,GEN1,HAT1,KIF18A,MAPRE1,NCAPG,SMC2	8
Cell Morphology,Cellular Function and Maintenance,DNA Replication, Recombination, and Repair	Double-stranded DNA break repair of cells	9.13E-05	Decreased	-2.45	BLM,BRCA1,DEK,DTL,PBRM1,PHF6,RBBP8,REV3L,RIF1,TP53,UCHL5,USP1,WRN	13
Cell Morphology,Cellular Assembly and Organization,Cellular Function and Maintenance	Formation of cilia	2.51E-04		-0.73	AKAP9,CCDC88A,CEP20,CEP290,CETN2,IFT74,KIAA0586,KIF2C,KIF3A,LIMA1,MKKS,PIBF1,PLK4,POC1B,PRKACB,SCLT1,TMEM135	17
Cell Morphology,Cellular Function and Maintenance	Repair of cells	2.86E-04	Decreased	-2.80	BLM,BRCA1,BRCA2,DEK,DTL,PBRM1,PHF6,RBBP8,REV3L,RIF1,TP53,UCHL5,USP1,WRN	14
Cell Morphology	Modification of tumor cell lines	2.98E-04	Decreased	-2.78	BLM,BRCA1,BRCA2,DEK,PBRM1,PHF6,RBBP8,REV3L,RIF1,TGM2,TP53,UCHL5,USP1	13
Cell Morphology,Cellular Assembly and Organization	Morphology of cytoskeleton	4.71E-04			CCP110,CKAP5,KIF11,KIF20A,KIF2C,NEDD1,TP53,TPX2,TRIP11	9
Cell Morphology,Cellular Function and Maintenance	Repair of tumor cell lines	5.51E-04	Decreased	-2.62	BLM,BRCA1,BRCA2,DEK,PBRM1,PHF6,RBBP8,REV3L,RIF1,TP53,UCHL5,USP1	12
Cell Morphology,Cellular Function and Maintenance	Homologous recombination repair of tumor cell lines	5.71E-04		-1.70	BRCA1,BRCA2,RBBP8,RIF1,TP53,UCHL5	6
Cell Morphology,Cellular Function and Maintenance,DNA Replication, Recombination, and Repair	Double-stranded DNA break repair of tumor cell lines	5.92E-04	Decreased	-2.42	BLM,BRCA1,DEK,PBRM1,PHF6,RBBP8,REV3L,RIF1,UCHL5,USP1	10
Cell Morphology,Cellular Function and Maintenance	Homologous recombination repair of bone cancer cell lines	7.15E-04	Decreased	-2.22	BRCA1,BRCA2,RBBP8,RIF1,UCHL5	5

Supplementary Table 5: Diseases and Biological Functions (mRNA) full dataset.

A complete list of genes, p-values and z-scores identified in the IPA generated subcategories of the disease and biological function categories; cell cycle, cellular structure; Damage related functions and RNA post-transcriptional modification.

DNA replication, recombination and repair

Categories	Diseases or Functions Annotation	p-value	Predicted Activation State	Activation z-score	Molecules	# Molecules
Cell Cycle,Cellular Assembly and Organization,DNA Replication, Recombination, and Repair	Segregation of chromosomes	3.24E-16		-0.46	BRCA1,CCNA2,CCNB1,CCNB2,CDK1,CENPE,CENPF,ECT2,GEN1,KIF11,KIF2C,MKI67,NCAPG,NCAPH,NDC80,NUF2,NUSAP1,PPP1R12A,SGO1,SKA1,SKA3,SMARCAD1,SMC2,SMC4,SMC6,SPC25,STAG2,TOP2A	28
Cellular Assembly and Organization,DNA Replication, Recombination, and Repair	Alignment of chromosomes	8.41E-10		-1.91	CCNA2,CENPE,DLGAP5,KIF14,KIF18A,KIF20A,KIF2C,NCAPG,SGO1,SMC4,TTK	11
DNA Replication, Recombination, and Repair	DNA replication	1.25E-07		-1.89	ATR,BLM,BRCA1,CCNA2,CDK1,DBF4,DHX9,FAM111B,FBXO5,MCM8,MGME1,MSH2,ORC2,ORC3,ORC5,POLA1,PRIM2,PRIMPOL,RBBP8,REV3L,SMC3,TP53,UCHL5,USP37,WRN	25
Cell Cycle,Cellular Assembly and Organization,DNA Replication, Recombination, and Repair	Duplication of centriole	2.15E-07		-1.94	AKAP9,CCP110,CEP135,CETN2,NEDD1,PLK4,SASS6,STIL	8
DNA Replication, Recombination, and Repair	Repair of DNA	2.31E-07	Decreased	-4.12	ASF1A,ATR,BCLAF1,BLM,BRCA1,BRCA2,CDK1,CETN2,DDX1,DEK,DHX9,DTL,ERCC4,EXD2,FANCF,GTF2H1,HAT1,HPF1,MLH1,MSH2,PBRM1,PHF6,POLA1,POLK,POLR2B,RBBP8,REV3L,RIF1,RRM1,SLF1,TIGAR,TOP2A,TP53,UBE2A,UBE2V2,UCHL5,USP1,WRN,XRCC4	39
DNA Replication, Recombination, and Repair	Double-stranded DNA break repair	4.74E-07		-1.58	ATR,BLM,BRCA1,BRCA2,CDK1,DDX1,DEK,DTL,ERCC4,EXD2,HPF1,PBRM1,PHF6,POLA1,RBBP8,REV3L,RIF1,SLF1,TP53,UBE2V2,UCHL5,USP1,WRN,XRCC4	24
DNA Replication, Recombination, and Repair	DNA damage response of cells	1.37E-06			APC,ATR,BLM,BRCA1,CDK1,DTL,EXD2,GEN1,HPF1,MCM8,MMS22L,POLK,PRIMPOL,REV3L,RIF1,SAMHD1,SLF1,SMC6,STXBP4,TANK,TIGAR,TOP2A,TP53,USP28,WDR76,WRN	26
Cellular Assembly and Organization,DNA Replication, Recombination, and Repair	Chromosomal congression of chromosomes	1.54E-06	Decreased	-2.24	CENPE,KIF14,KIF18A,KIF2C,NDC80,SGO2	6
Cell Cycle,Cellular Assembly and Organization,DNA Replication, Recombination, and Repair	Replication of centriole	2.07E-06		-1.94	AKAP9,CCP110,CEP135,CETN2,NEDD1,PLK4,POC1B,SASS6,STIL	9
DNA Replication, Recombination, and Repair	Metabolism of DNA	3.21E-06	Decreased	-2.61	APOBEC3B,ATR,BLM,BRCA1,BRCA2,CCNA2,CDK1,DBF4,DHX9,EIF2AK2,FAM111B,FBXO5,HMGB2,KPNA1,MCM8,MGME1,MSH2,ORC2,ORC3,ORC5,POLA1,PRIM2,PRIMPOL,RBBP8,REV3L,SMC3,TP53,UCHL5,USP37,WRN	30
Cell Cycle,Cellular Assembly and Organization,Cellular Function and Maintenance,DNA Replication, Recombination, and Repair	Duplication of centrosome	5.66E-06			BRCA2,CCP110,CHMP2B,GEN1,NDC80,SASS6,STIL,TP53,TTK	9
DNA Replication, Recombination, and Repair	Condensation of chromosomes	7.54E-06			MAD2L1,NCAPG,NCAPH,NUSAP1,SMC2,SMC4,TOP2A	7
Cell Cycle,Cell Morphology,Cellular Assembly and Organization,DNA Replication, Recombination, and Repair	Morphology of chromosomes	1.57E-05			APC,CENPE,GEN1,HAT1,KIF18A,MAPRE1,NCAPG,SMC2	8
Cell Cycle,DNA Replication, Recombination, and Repair	Checkpoint control	2.12E-05		-1.35	ATR,CCNB1,CCNB2,CKS2,DBF4,MAD2L1,NDC80,RBBP8,TP53,ZWILCH	10
Cell Cycle,DNA Replication, Recombination, and Repair	Homologous recombination of DNA	3.36E-05		0.17	BLM,BRCA1,BRCA2,ENY2,MMS22L,TP53,WRN	7
DNA Replication, Recombination, and Repair	Condensation of mitotic chromosomes	5.73E-05			NCAPG,NCAPH,NUSAP1,SMC2,SMC4	5
Cell Cycle,Cellular Assembly and Organization,DNA Replication, Recombination, and Repair	Segregation of sister chromatids	6.86E-05			CCNA2,NDC80,NUSAP1,PPP1R12A,SMC4,STAG2	6
Cellular Assembly and Organization,DNA Replication, Recombination, and Repair	Formation of nuclear foci	7.94E-05	Increased	2.20	BLM,BRCA1,BRCA2,CCNA2,CCNB1,CDK1,LMNB1,MMS22L,MSH2,POT1,RBBP8,REV3L,TP53,WRN	14
Cell Morphology,Cellular Function and Maintenance,DNA Replication, Recombination, and Repair	Double-stranded DNA break repair of cells	9.13E-05	Decreased	-2.45	BLM,BRCA1,DEK,DTL,PBRM1,PHF6,RBBP8,REV3L,RIF1,TP53,UCHL5,USP1,WRN	13
Cellular Assembly and Organization,DNA Replication, Recombination, and Repair	Alignment of sister chromatids	1.75E-04			NDC80,TOP2A,TOP2B	3
Cell Cycle,DNA Replication, Recombination, and Repair	Processing of replication fork	3.12E-04			BLM,EXD2,GEN1,MMS22L,PRIMPOL,WRN	6
Cellular Assembly and Organization,DNA Replication, Recombination, and Repair	Quantity of chromosomes	3.29E-04		1.00	CENPE,CENPF,KIF2C,MAD2L1,TP53	5
Cell Morphology,Cellular Function and Maintenance,DNA Replication, Recombination, and Repair	Double-stranded DNA break repair of tumor cell lines	5.92E-04	Decreased	-2.42	BLM,BRCA1,DEK,PBRM1,PHF6,RBBP8,REV3L,RIF1,UCHL5,USP1	10
Cell Cycle,DNA Replication, Recombination, and Repair	DNA damage checkpoint	6.42E-04			ATR,CDC5L,CLOCK,RPS27L,TIPRL,TP53,USP28	7
Cellular Compromise,DNA Replication, Recombination, and Repair	Chromosomal instability	7.44E-04		1.25	BCLAF1,BRCA1,CKAP5,PSME4,RRM1,SMC3,TP53	7
DNA Replication, Recombination, and Repair	Chromosomal aberration	7.52E-04		1.73	ALMS1,BCLAF1,BRCA1,CKAP5,E2F5,ECT2,GABPA,KIF11,POLA1,PRIM2,PSME4,RRM1,RRM2,SAMD9,SAMD9L,SMC3,TLR10,TP53,USP25	19

Supplementary Table 5: Diseases and Biological Functions (mRNA) full dataset.

A complete list of genes, p-values and z-scores identified in the IPA generated subcategories of the disease and biological function categories; cell cycle, cellular structure; Damage related functions and RNA post-transcriptional modification.

Cell death and survival

Categories	Diseases or Functions Annotation	p-value	Predicted Activation State	Activation z-score	Molecules	# Molecules
Cell Death and Survival	Cell viability of tumor cell lines	1.35E-09	Decreased	-5.68	ADAM17,ADK,AGPS,ANLN,ASF1A,ATP5MK,ATR,BCLAF1,BLM,BNIP2,BRCA1,BRCA2,BTK,C1GALT1,CASK,CCNA2,CCNB1,CDCA8,CDK8,CKAP5,COMMD3-BMI1,COP1,CSE1L,DPP8,DUSP10,EIF2AK4,EIF2S1,EIF4E,EPB41L2,FAM111B,FANCB,FBXO5,FKBP5,HLTF,HSPA4,HSPH1,KHK,KIF11,KIF15,KIF18A,KNL1,LILRB4,LIMS1,LMNB1,LSM8,MAD2L1,MAPK8,MAPK9,MASTL,MELK,MLH1,MMS22L,MSH2,NDC80,NUF2,NUP155,PBK,PBRM1,PBX3,PIK3R1,PLK4,PPAT,PRKACB,PSMA3,PSMA4,PSME4,PTPN22,PTPN9,REV3L,RPS6KA6,RRM1,RRM2,RSF1,SKA1,SKA3,SMC4,SMC6,SOS1,SRSF3,STK3,TGM2,TOP2A,TP53,TPMT,TPX2,TRIM21,TRIM37,TRMT5,TTK,TUBA1B,UCHL5,UTP15,VBP1,VRK1,WRN,XRCC4,XRN1,ZNF100,ZNF429,ZNF43,ZNF91	101
Cell Death and Survival	Cell survival	1.47E-08	Decreased	-6.20	ADAM17,ADK,AGPS,ANLN,ANTXR2,ANXA5,APC,ASF1A,ATP5MK,ATR,BCLAF1,BLM,BNIP2,BRCA1,BRCA2,BTK,C1GALT1,CASK,CCNA2,CCNB1,CDCA8,CDK1,CDK8,CKAP5,COMMD3-BMI1,COP1,CSE1L,DPP8,DUSP10,EIF2AK4,EIF2S1,EIF4E,EMC2,EPB41L2,ERCC4,FAM111B,FANCB,FBXO5,FKBP5,HLTF,HMGCS1,HSPA4,HSPH1,IL6ST,KHK,KIF11,KIF15,KIF18A,KNL1,LILRB4,LIMS1,LMNB1,LSM8,MAD2L1,MAPK8,MAPK9,MASTL,MELK,METAP2,MGST1,MLH1,MMS22L,MSH2,NDC80,NUF2,NUP155,PBK,PBRM1,PBX3,PIK3R1,PLK4,PPAT,PRKACB,PSMA3,PSMA4,PSME4,PTPN22,PTPN9,REV3L,RIT1,RPS6KA6,RRM1,RRM2,RRN3,RSF1,S1PR1,SKA1,SKA3,SMC4,SMC6,SOS1,SQLE,SRSF3,STK3,TAF1B,TGM2,TOP2A,TP53,TPMT,TPX2,TRIM21,TRIM37,TRMT5,TTK,TUBA1B,UCHL5,UTP15,VBP1,VRK1,WRN,XRCC4,XRN1,ZNF100,ZNF429,ZNF43,ZNF91	116
Cell Death and Survival	Cell viability	3.81E-08	Decreased	-5.97	ADAM17,ADK,AGPS,ANLN,ANTXR2,ANXA5,APC,ASF1A,ATP5MK,ATR,BCLAF1,BLM,BNIP2,BRCA1,BRCA2,BTK,C1GALT1,CASK,CCNA2,CCNB1,CDCA8,CDK1,CDK8,CKAP5,COMMD3-BMI1,COP1,CSE1L,DPP8,DUSP10,EIF2AK4,EIF2S1,EIF4E,EMC2,EPB41L2,FAM111B,FANCB,FBXO5,FKBP5,HLTF,HMGCS1,HSPA4,HSPH1,KHK,KIF11,KIF15,KIF18A,KNL1,LILRB4,LIMS1,LMNB1,LSM8,MAD2L1,MAPK8,MAPK9,MASTL,MELK,METAP2,MGST1,MLH1,MMS22L,MSH2,NDC80,NUF2,NUP155,PBK,PBRM1,PBX3,PIK3R1,PLK4,PPAT,PRKACB,PSMA3,PSMA4,PSME4,PTPN22,PTPN9,REV3L,RPS6KA6,RRM1,RRM2,RSF1,S1PR1,SKA1,SKA3,SMC4,SMC6,SOS1,SQLE,SRSF3,STK3,TAF1B,TGM2,TOP2A,TP53,TPMT,TPX2,TRIM21,TRIM37,TRMT5,TTK,TUBA1B,UCHL5,UTP15,VBP1,VRK1,WRN,XRCC4,XRN1,ZNF100,ZNF429,ZNF43,ZNF91	112
Cell Death and Survival	Cell death of cervical cancer cell lines	3.61E-07	Increased	2.18	ANXA5,ARHGEF6,BAZ1A,BNIP2,BRCA1,BRCA2,CCNB1,CDCA2,CDK1,CDK8,CENPE,CENPF,CKAP5,DHX9,EEA1,EIF2AK2,EIF4E,FASTKD2,HSPB11,KIF11,KIF14,KIF15,KNL1,LIMS1,LYPLA1,MAD2L1,MAPK8,MMS22L,NDC80,NUF2,OPA1,PBK,PLK4,POT1,SKA3,SMC6,SPC25,STK3,TBL1XR1,TOP2A,TOP2B,TP53,TPX2,TTK	44
Cell Death and Survival	Cell viability of cervical cancer cell lines	7.49E-07	Decreased	-3.98	ADK,ASF1A,ATP5MK,ATR,BLM,BNIP2,BRCA1,BRCA2,CASK,CCNA2,CDK8,DUSP10,EIF2AK4,KHK,KIF15,MMS22L,PRKACB,PSME4,PTPN22,PTPN9,REV3L,RPS6KA6,SKA1,SKA3,STK3,TTK,VBP1,WRN,XRCC4	29
Cell Death and Survival	Apoptosis of cervical cancer cell lines	3.01E-06		1.83	ANXA5,ARHGEF6,BAZ1A,BNIP2,BRCA1,BRCA2,CCNB1,CDCA2,CDK1,CDK8,CENPE,CENPF,CKAP5,EEA1,EIF2AK2,FASTKD2,KIF11,KIF14,KNL1,LIMS1,LYPLA1,MAD2L1,MAPK8,NDC80,NUF2,OPA1,PBK,PLK4,POT1,SPC25,STK3,TBL1XR1,TP53,TPX2,TTK	35
Cell Death and Survival	Cell survival of tumor cell lines	1.18E-05		0.34	ATR,BTK,COMMD3-BMI1,FANCB,FBXO5,KIF11,KIF15,MAPK9,MLH1,MSH2,RRM1,SMC6,TOP2A,TP53,TRMT5,TUBA1B,UCHL5,XRN1	18
Cell Death and Survival	Cell viability of bone cancer cell lines	1.29E-05		-1.75	ATR,BLM,BRCA1,BRCA2,DPP8,FAM111B,HLTF,LMNB1,MAPK8,MAPK9,MMS22L,TP53,TTK,UCHL5,WRN	15
Cell Death and Survival	Cell death of tumor cell lines	5.47E-05	Increased	2.24	ABCE1,ADAM17,ANXA5,APC,ARHGEF6,ATAD2,ATR,BAZ1A,BCLAF1,BLM,BNIP2,BRCA1,BRCA2,BTK,C1GALT1,CAST,CCDC88A,CCNB1,CCP110,CCT2,CDCA2,CDCP1,CDK1,CDK8,CENPE,CENPF,CENPI,CHUK,CKAP2,CKAP2L,CKAP5,CKS2,COMMD3-BMI1,COP1,CSE1L,DDX58,DEK,DEPDC1,DHX9,DLG1,DNAJC15,DPP8,DTL,EEA1,EIF2AK2,EIF2AK4,EIF2S1,EIF4E,FAM111B,FANCF,FASTKD2,FBXO5,FKBP5,GPR65,HAT1,HMGCS1,HMMR,HSPA4,HSPB11,HSPH1,IDE,IFI16,IFIH1,IL6ST,KIF11,KIF14,KIF15,KNL1,LIMS1,LMNB1,LYPLA1,MAD2L1,MAPK8,MAPK9,MELK,MLH1,MMS22L,MSH2,MYO6,NCL,NDC80,NOB1,NQO1,NUF2,OPA1,OSBPL8,PBK,PBX3,PIK3R1,PLK4,PLSCR1,POLA1,POLK,POT1,PPAT,PPID,PRKACB,RAP1GDS1,RPAP3,RPS27L,RRM1,RRM2,SDHC,SKA3,SMC6,SMURF2,SP1,SPC25,STK3,TBL1XR1,TEX10,TGM2,TOP2A,TOP2B,TP53,TPX2,TRIM24,TRIM37,TSG101,TTK,UBA3,UCHL5,WRN,ZNF148,ZNF382	125

Cell Death and Survival	Apoptosis	1.05E-04		1.22	ABCE1,ADAM17,AKTIP,ANTXR2,ANXA5,APC,APOBEC3B,ARHGEF6,ATAD2,ATR,BAZ1A,BCLAF1,BLM,BNIP2,BRCA1,BRCA2,BTK,C1GALT1,CAST,CCNA2,CCNB1,CCR6,CCT2,CDCA2,CDCP1,CDK1,CDK8,CENPE,CENPF,CHUK,CKAP2,CKAP5,CKS2,COMMD3-BMI1,COP1,CSE1L,CUL5,DDX20,DDX58,DEK,DEPDC1,DGKE,DHX9,DLG1,DNAJC15,ECT2,EEA1,EIF2AK2,EIF2AK4,EIF4E,FAM111B,FANCF,FASTKD2,FBXO5,FKBP5,GPR65,HAT1,HMGB2,HMGN5,HMMR,HSPA4,IDE,IFI16,IFIH1,IFIT2,IFIT3,IFNAR1,IL6ST,KIF11,KIF14,KNL1,KPNA1,LIMS1,LMNB1,LYPLA1,MAD2L1,MAPK8,MAPK9,MCTS1,MELK,MLH1,MSH2,MYO6,NAA15,NCL,NDC80,NMI,NQO1,NUF2,OPA1,ORC2,OSBPL8,PBK,PBX3,PDCD10,PIK3R1,PKN2,PLK4,PLSCR1,PNPT1,POLA1,POT1,PPID,PRKACB,RBM7,ROCK1,RPS27L,RRM1,RRM2,S1PR1,SDHC,SDHD,SLC9A6,SMURF2,SOS1,SP1,SPC25,STK3,TBL1XR1,TFCP2,TGM2,TIGAR,TLR1,TOMM70,TOP2A,TP53,TPX2,TRIM24,TRIM37,TSG101,TTK,UBA3,UCHL5,UTP11,WRN,ZNF148,ZNF382	137
Cell Death and Survival	Cell viability of myeloma cell lines	2.31E-04	Decreased	-3.05	BTK,CKAP5,KIF11,KIF18A,MAPK9,NDC80,NUF2,PSMA3,PSMA4,RRM1,RSF1,TPMT,TRIM21	13
Cell Death and Survival,Cellular Compromise	Mitotic catastrophe of tumor cell lines	2.45E-04		1.05	CCP110,EIF4E,MAD2L1,SMC6,TP53	5
Cell Death and Survival	Necrosis	3.53E-04	Increased	2.06	ABCE1,ADAM17,AKTIP,ANTXR2,ANXA5,APC,APOBEC3B,ARHGEF6,ATAD2,ATR,BAZ1A,BCLAF1,BLM,BNIP2,BRCA1,BRCA2,BTK,C1GALT1,CAST,CCDC88A,CCNB1,CCP110,CCR6,CCT2,CDCA2,CDCP1,CDK1,CDK8,CENPE,CENPF,CENPI,CHUK,CKAP2,CKAP2L,CKAP5,CKS2,COMMD3-BMI1,COP1,CSE1L,DDX58,DEK,DEPDC1,DGKE,DHX9,DLG1,DNAJC15,DPP8,DTL,EEA1,EIF2AK2,EIF2AK4,EIF2S1,EIF4E,FAM111B,FANCF,FASTKD2,FBXO5,FKBP5,GPR65,HAT1,HMGCS1,HMMR,HSPA4,HSPB11,HSPH1,IDE,IFI16,IFIH1,IFNAR1,IL6ST,KIF11,KIF14,KIF15,KNL1,LIMS1,LMNB1,LYPLA1,MAD2L1,MAPK8,MAPK9,MCTS1,MELK,MLH1,MMS22L,MSH2,MYO6,NCL,NDC80,NMI,NOB1,NQO1,NUF2,OPA1,OSBPL8,PBK,PBX3,PDE3B,PIK3R1,PKN2,PLK4,PLSCR1,PNPT1,POLA1,POLK,POT1,PPAT,PPID,PRKACB,RAP1GDS1,RBM7,REV3L,ROCK1,RPAP3,RPS27L,RRM1,RRM2,S1PR1,SDHC,SDHD,SKA3,SLC9A6,SMC6,SMURF2,SP1,SPC25,SQLE,STK3,TAF1B,TBL1XR1,TEX10,TGM2,TLR1,TOP2A,TOP2B,TP53,TPX2,TRIM24,TRIM37,TSG101,TTK,UBA3,UCHL5,WRN,ZNF148,ZNF382	145
Cell Death and Survival	Apoptosis of tumor cell lines	6.37E-04		1.01	ABCE1,ADAM17,ANXA5,APC,ARHGEF6,ATAD2,ATR,BAZ1A,BCLAF1,BLM,BNIP2,BRCA1,BRCA2,BTK,C1GALT1,CAST,CCNB1,CCT2,CDCA2,CDCP1,CDK1,CDK8,CENPE,CENPF,CHUK,CKAP2,CKAP5,CKS2,COMMD3-BMI1,COP1,CSE1L,DDX58,DEPDC1,DHX9,DLG1,DNAJC15,EEA1,EIF2AK2,EIF2AK4,EIF4E,FAM111B,FANCF,FASTKD2,FBXO5,FKBP5,GPR65,HAT1,HMMR,HSPA4,IDE,IFI16,KIF11,KIF14,KNL1,LIMS1,LMNB1,LYPLA1,MAD2L1,MAPK8,MAPK9,MELK,MLH1,MSH2,MYO6,NCL,NDC80,NQO1,NUF2,OPA1,OSBPL8,PBK,PBX3,PLK4,POLA1,POT1,PPID,PRKACB,RPS27L,RRM1,RRM2,SDHC,SMURF2,SP1,SPC25,STK3,TBL1XR1,TGM2,TOP2A,TP53,TPX2,TRIM24,TRIM37,TSG101,TTK,UCHL5,WRN,ZNF148,ZNF382	98
Cell Death and Survival	Survival of eye cell lines	8.30E-04			BRCA1,MGST1,STK3	3

Supplementary Table 5: Diseases and Biological Functions (mRNA) full dataset.

A complete list of genes, p-values and z-scores identified in the IPA generated subcategories of the disease and biological function categories; cell cycle, cellular structure; Damage related functions and RNA post-transcriptional modification.

Cellular function and maintainence

Categories	Diseases or Functions Annotation	p-value	Predicted Activation State	Activation z-score	Molecules	# Molecules
Cellular Assembly and Organization,Cellular Function and Maintenance	Organization of cytoplasm	1.54E-08	Decreased	-2.32	ABCD3,ACTN1,AKAP9,AKTIP,APC,ASPM,ATL3,BLZF1,BORA,BTK,CARMIL1,CCDC88A,CCNB1,CD2AP,CDK1,CENPE,CEP20,CEP290,CEP350,CETN2,CHMP2B,CHUK,CKAP5,DAAM1,DIAPH2,DIAPH3,DLG1,DLGAP5,DNAJC13,DOCK5,DOCK7,ECT2,EIF4E,GABPA,GAS2L3,GCC2,GPR65,HMMR,HOOK1,IFT74,KIAA0586,KIF11,KIF18A,KIF20B,KIF2A,KIF2C,KIF3A,LARP4,LIMA1,LYSMD3,MAD2L1,MAPRE1,MKKS,MTBP,NDC80,NEDD1,OPA1,PIBF1,PIK3R1,PIP5K1A,PLK4,POC1B,PRKACB,PRPF40A,RAP2A,ROCK1,SASS6,SCLT1,SEPTIN7,SLC9A6,SPC25,STIL,STXBP3,TBCE,TGM2,TMEM135,TOR1AIP2,TP53,TPX2,TTK,ZFP69B,ZMYM4	82
Cellular Assembly and Organization,Cellular Function and Maintenance	Organization of cytoskeleton	2.36E-07	Decreased	-2.32	ACTN1,AKAP9,APC,ASPM,BORA,BTK,CARMIL1,CCDC88A,CCNB1,CD2AP,CDK1,CENPE,CEP20,CEP290,CEP350,CETN2,CHUK,CKAP5,DAAM1,DIAPH2,DIAPH3,DLG1,DLGAP5,DOCK5,DOCK7,ECT2,EIF4E,GAS2L3,GCC2,GPR65,HMMR,IFT74,KIAA0586,KIF11,KIF18A,KIF20B,KIF2A,KIF2C,KIF3A,LARP4,LIMA1,MAD2L1,MAPRE1,MKKS,MTBP,NDC80,NEDD1,PIBF1,PIK3R1,PIP5K1A,PLK4,POC1B,PRKACB,PRPF40A,RAP2A,ROCK1,SASS6,SCLT1,SEPTIN7,SLC9A6,SPC25,STIL,STXBP3,TBCE,TGM2,TMEM135,TP53,TPX2,TTK,ZMYM4	70
Cellular Assembly and Organization,Cellular Function and Maintenance	Organization of mitotic spindle	2.70E-07			ASPM,BORA,CCNB1,CENPE,CKAP5,KIF11,KIF2A,NDC80,SPC25,STIL,TBCE,TPX2,TTK	13
Cell Cycle,Cellular Assembly and Organization,Cellular Function and Maintenance,DNA Replication, Recombination, and Repair	Duplication of centrosome	5.66E-06			BRCA2,CCP110,CHMP2B,GEN1,NDC80,SASS6,STIL,TP53,TTK	9
Cellular Assembly and Organization,Cellular Function and Maintenance	Microtubule dynamics	6.32E-06	Decreased	-2.37	AKAP9,APC,BTK,CARMIL1,CCDC88A,CD2AP,CDK1,CEP20,CEP290,CEP350,CETN2,CHUK,CKAP5,DAAM1,DIAPH2,DIAPH3,DLGAP5,DOCK5,DOCK7,ECT2,EIF4E,GAS2L3,GCC2,HMMR,IFT74,KIAA0586,KIF11,KIF18A,KIF20B,KIF2A,KIF2C,KIF3A,LIMA1,MAD2L1,MAPRE1,MKKS,MTBP,NEDD1,PIBF1,PIK3R1,PLK4,POC1B,PRKACB,RAP2A,ROCK1,SASS6,SCLT1,SEPTIN7,SLC9A6,STXBP3,TBCE,TMEM135,TP53,TPX2	54
Cellular Assembly and Organization,Cellular Function and Maintenance	Organization of nuclear pores	4.50E-05			NUP133,SEH1L,TPR	3
Cell Morphology,Cellular Function and Maintenance,DNA Replication, Recombination, and Repair	Double-stranded DNA break repair of cells	9.13E-05	Decreased	-2.45	BLM,BRCA1,DEK,DTL,PBRM1,PHF6,RBBP8,REV3L,RIF1,TP53,UCHL5,USP1,WRN	13
Cell Morphology,Cellular Assembly and Organization,Cellular Function and Maintenance	Formation of cilia	2.51E-04		-0.73	AKAP9,CCDC88A,CEP20,CEP290,CETN2,IFT74,KIAA0586,KIF2C,KIF3A,LIMA1,MKKS,PIBF1,PLK4,POC1B,PRKACB,SCLT1,TMEM135	17
Cell Morphology,Cellular Function and Maintenance	Repair of cells	2.86E-04	Decreased	-2.80	BLM,BRCA1,BRCA2,DEK,DTL,PBRM1,PHF6,RBBP8,REV3L,RIF1,TP53,UCHL5,USP1,WRN	14
Cell Morphology,Cellular Function and Maintenance	Repair of tumor cell lines	5.51E-04	Decreased	-2.62	BLM,BRCA1,BRCA2,DEK,PBRM1,PHF6,RBBP8,REV3L,RIF1,TP53,UCHL5,USP1	12
Cell Morphology,Cellular Function and Maintenance	Homologous recombination repair of tumor cell lines	5.71E-04		-1.70	BRCA1,BRCA2,RBBP8,RIF1,TP53,UCHL5	6
Cell Morphology,Cellular Function and Maintenance,DNA Replication, Recombination, and Repair	Double-stranded DNA break repair of tumor cell lines	5.92E-04	Decreased	-2.42	BLM,BRCA1,DEK,PBRM1,PHF6,RBBP8,REV3L,RIF1,UCHL5,USP1	10
Cell Morphology,Cellular Function and Maintenance	Homologous recombination repair of bone cancer cell lines	7.15E-04	Decreased	-2.22	BRCA1,BRCA2,RBBP8,RIF1,UCHL5	5
Cellular Assembly and Organization,Cellular Function and Maintenance	Density of microtubules	8.30E-04			APC,CKAP5,KIF2C	3

Supplementary Table 5: Diseases and Biological Functions (mRNA) full dataset.

A complete list of genes, p-values and z-scores identified in the IPA generated subcategories of the disease and biological function categories; cell cycle, cellular structure; Damage related functions and RNA post-transcriptional modification.

Cellular compromise

Categories	Diseases or Functions Annotation	p-value	Predicted Activation State	Activation z-score	Molecules	# Molecules
Cellular Compromise	Nucleation of cells	6.33E-08		1.53	BRCA1,BRCA2,DIAPH3,ECT2,GEN1,KIF14,KIF23,MAD2L1,PPP1R12A,SEPTIN11,SEPTIN7,TP53	12
Cell Morphology,Cellular Compromise	Multinucleation of cells	2.85E-07		0.98	BRCA2,DIAPH3,ECT2,GEN1,KIF14,KIF23,MAD2L1,PPP1R12A,SEPTIN11,SEPTIN7,TP53	11
Cellular Compromise	Nucleation of tumor cell lines	3.39E-07		1.25	BRCA1,DIAPH3,ECT2,GEN1,KIF14,KIF23,PPP1R12A,SEPTIN11,SEPTIN7,TP53	10
Cell Morphology,Cellular Compromise	Multinucleation of tumor cell lines	2.07E-06		1.02	DIAPH3,ECT2,GEN1,KIF14,KIF23,PPP1R12A,SEPTIN11,SEPTIN7,TP53	9
Cell Morphology,Cellular Compromise	Multinucleation of cervical cancer cell lines	3.65E-06		1.25	DIAPH3,GEN1,KIF14,KIF23,SEPTIN11,SEPTIN7,TP53	7
Cell Morphology,Cellular Compromise	Binucleation of cervical cancer cell lines	7.75E-06		0.00	DIAPH3,SEPTIN11,SEPTIN7,TP53	4
Cell Morphology,Cellular Compromise	Binucleation of tumor cell lines	1.23E-05		0.37	DIAPH3,ECT2,SEPTIN11,SEPTIN7,TP53	5
Cell Death and Survival,Cellular Compromise	Mitotic catastrophe of tumor cell lines	2.45E-04		1.05	CCP110,EIF4E,MAD2L1,SMC6,TP53	5
Cellular Compromise	Dysfunction of tumor cell lines	3.12E-04		0.53	CCP110,EIF4E,MAD2L1,SMC6,TP53,TRIM37	6
Cellular Compromise,DNA Replication, Recombination, and Repair	Chromosomal instability	7.44E-04		1.25	BCLAF1,BRCA1,CKAP5,PSME4,RRM1,SMC3,TP53	7

Supplementary Table 5: Diseases and Biological Functions (mRNA) full dataset.

A complete list of genes, p-values and z-scores identified in the IPA generated subcategories of the disease and biological function categories; cell cycle, cellular structure; Damage related functions and RNA post-transcriptional modification.

Post-trancriptional modification

Categories	Diseases or Functions Annotation	p-value	Predicted Activation State	Activation z-score	Molecules	# Molecules
RNA Post-Transcriptional Modification	Processing of rRNA	3.72E-10			DDX52,DIMT1,ERI1,EXOSC8,FCF1,KRR1,NOB1,NOL11,NOLC1,NOP58,RRP36,TEX10,UTP11,UTP14A,UTP15,UTP20,UTP6,WDR12,WDR3,WDR75	20
RNA Post-Transcriptional Modification	Processing of RNA	1.25E-08		-1.93	CDC5L,CPSF2,CPSF3,CSTF3,DDX20,DDX46,DDX52,DHX9,DIMT1,ERI1,EXOSC8,FASTKD2,FCF1,GCFC2,GTF2H1,KRR1,LSM5,LSM8,NCBP1,NOB1,NOL11,NOLC1,NOP58,POLR2B,PRPF40A,RRP36,RSRC1,SCAF11,SF3B6,SNRNP48,SRSF3,TEX10,UPF3B,UTP11,UTP14A,UTP15,UTP20,UTP6,WBP4,WDR12,WDR3,WDR75,ZNF326	43

Supplementary Table 7: Diseases and Biological Functions (miRNA) full data set

A complete list of miRNA and p-values identified in the different subcategories of the disease and biological function in IPA

Category	p-value	# molecules (miRNA)	OASIS miRNA nomenclature	miRNA symbols [IPA miRNA nomenclature]
Neurological Disease	2.05E-09-2.67E-02	7	miR-99b-5p, miR-212-3p, miR-143-3p, miR-181d-5p, miR-616-5p, miR29c-3p, miR-3196	miR-100-5p (and other miRNAs w/seed ACCCGUA),miR-132-3p (and other miRNAs w/seed AACAGUC),miR-143-3p (and other miRNAs w/seed GAGAUGA),miR-181a-5p (and other miRNAs w/seed ACAUUCA),miR-292b-5p (and other miRNAs w/seed CUCAAAA),miR-29b-3p (and other miRNAs w/seed AGCACCA),miR-3180-3p (and other miRNAs w/seed GGGGCGG)
Organismal Injury and Abnormalities	2.05E-09-4.92E-02	13	miR-99b-5p, miR-212-3p, miR-143-3p, miR-150-5p, miR-181d-5p, miR-24-2-5p, miR-616-5p, miR29c-3p, miR-3196, miR-330-3p, miR-501-3p, miR-505-3p, miR-941	miR-100-5p (and other miRNAs w/seed ACCCGUA),miR-132-3p (and other miRNAs w/seed AACAGUC),miR-143-3p (and other miRNAs w/seed GAGAUGA),miR-150-5p (and other miRNAs w/seed CUCCCAA),miR-181a-5p (and other miRNAs w/seed ACAUUCA),miR-24-1-5p (and other miRNAs w/seed GCCUACU),miR-292b-5p (and other miRNAs w/seed CUCAAAA),miR-29b-3p (and other miRNAs w/seed AGCACCA),miR-3180-3p (and other miRNAs w/seed GGGGCGG),miR-330-3p (and other miRNAs w/seed CAAAGCA),miR-501-3p (and other miRNAs w/seed AUGCACC),miR-505-3p (miRNAs w/seed GUCAACA),miR-941 (miRNAs w/seed ACCCGGC)
Psychological Disorders	2.05E-09-2.67E-02	7	miR-99b-5p, miR-212-3p, miR-143-3p, miR-181d-5p, miR-616-5p, miR29c-3p, miR-3196	miR-100-5p (and other miRNAs w/seed ACCCGUA),miR-132-3p (and other miRNAs w/seed AACAGUC),miR-143-3p (and other miRNAs w/seed GAGAUGA),miR-181a-5p (and other miRNAs w/seed ACAUUCA),miR-292b-5p (and other miRNAs w/seed CUCAAAA),miR-29b-3p (and other miRNAs w/seed AGCACCA),miR-3180-3p (and other miRNAs w/seed GGGGCGG)
Cancer	1.79E-07-4.92E-02	8	miR-99b-5p, miR-212-3p, miR-143-3p, miR-150-5p, miR-181d-5p, miR-24-2-5p, miR-616-5p, miR29c-3p,	miR-100-5p (and other miRNAs w/seed ACCCGUA),miR-132-3p (and other miRNAs w/seed AACAGUC),miR-143-3p (and other miRNAs w/seed GAGAUGA),miR-150-5p (and other miRNAs w/seed CUCCCAA),miR-181a-5p (and other miRNAs w/seed ACAUUCA),miR-24-1-5p (and other miRNAs w/seed GCCUACU),miR-292b-5p (and other miRNAs w/seed CUCAAAA),miR-29b-3p (and other miRNAs w/seed AGCACCA)
Reproductive System Disease	3.96E-07-3.3E-02	8	miR-99b-5p, miR-212-3p, miR-143-3p, miR-150-5p, miR-181d-5p, miR-24-2-5p, miR29c-3p, miR-505-3p	miR-100-5p (and other miRNAs w/seed ACCCGUA),miR-132-3p (and other miRNAs w/seed AACAGUC),miR-143-3p (and other miRNAs w/seed GAGAUGA),miR-150-5p (and other miRNAs w/seed CUCCCAA),miR-181a-5p (and other miRNAs w/seed ACAUUCA),miR-24-1-5p (and other miRNAs w/seed GCCUACU),miR-29b-3p (and other miRNAs w/seed AGCACCA),miR-505-3p (miRNAs w/seed GUCAACA)
Cardiovascular Disease	3.48E-05-1.73E-02	3	miR-150-5p, miR-181d-5p, miR29c-3p	miR-150-5p (and other miRNAs w/seed CUCCCAA),miR-181a-5p (and other miRNAs w/seed ACAUUCA),miR-29b-3p (and other miRNAs w/seed AGCACCA)
Gastrointestinal Disease	7.16E-05-4.86E-02	8	miR-99b-5p, miR-212-3p, miR-143-3p, miR-150-5p, miR-181d-5p, miR29c-3p, miR-330-3p, miR-941	miR-100-5p (and other miRNAs w/seed ACCCGUA),miR-132-3p (and other miRNAs w/seed AACAGUC),miR-143-3p (and other miRNAs w/seed GAGAUGA),miR-150-5p (and other miRNAs w/seed CUCCCAA),miR-181a-5p (and other miRNAs w/seed ACAUUCA),miR-29b-3p (and other miRNAs w/seed AGCACCA),miR-330-3p (and other miRNAs w/seed CAAAGCA),miR-941 (miRNAs w/seed ACCCGGC)
Respiratory Disease	7.16E-05-4.56E-02	3	miR-99b-5p, miR-143-3p, miR29c-3p	miR-100-5p (and other miRNAs w/seed ACCCGUA),miR-143-3p (and other miRNAs w/seed GAGAUGA),miR-29b-3p (and other miRNAs w/seed AGCACCA)
Endocrine System Disorders	1.21E-04-4.86E-02	6	miR-99b-5p, miR-212-3p, miR-143-3p, miR-150-5p, miR-181d-5p, miR29c-3p	miR-100-5p (and other miRNAs w/seed ACCCGUA),miR-132-3p (and other miRNAs w/seed AACAGUC),miR-143-3p (and other miRNAs w/seed GAGAUGA),miR-150-5p (and other miRNAs w/seed CUCCCAA),miR-181a-5p (and other miRNAs w/seed ACAUUCA),miR-29b-3p (and other miRNAs w/seed AGCACCA)
Hematological Disease	1.98E-04-3.54E-02	7	miR-99b-5p, miR-212-3p, miR-143-3p, miR-150-5p, miR-181d-5p, miR-616-5p, miR29c-3p	miR-100-5p (and other miRNAs w/seed ACCCGUA),miR-132-3p (and other miRNAs w/seed AACAGUC),miR-143-3p (and other miRNAs w/seed GAGAUGA),miR-150-5p (and other miRNAs w/seed CUCCCAA),miR-181a-5p (and other miRNAs w/seed ACAUUCA),miR-292b-5p (and other miRNAs w/seed CUCAAAA),miR-29b-3p (and other miRNAs w/seed AGCACCA)
Immunological Disease	1.98E-04-3.54E-02	6	miR-212-3p, miR-143-3p, miR-150-5p, miR-181d-5p, miR-616-5p, miR29c-3p	miR-132-3p (and other miRNAs w/seed AACAGUC),miR-143-3p (and other miRNAs w/seed GAGAUGA),miR-150-5p (and other miRNAs w/seed CUCCCAA),miR-181a-5p (and other miRNAs w/seed ACAUUCA),miR-292b-5p (and other miRNAs w/seed CUCAAAA),miR-29b-3p (and other miRNAs w/seed AGCACCA)
Developmental Disorder	2.59E-04-2.59E-04	2	miR-99b-5p, miR-501-3p	miR-100-5p (and other miRNAs w/seed ACCCGUA),miR-501-3p (and other miRNAs w/seed AUGCACC)
Hereditary Disorder	2.59E-04-3.47E-04	3	miR-99b-5p, miR-29c-3p, miR-501-3p	miR-100-5p (and other miRNAs w/seed ACCCGUA),miR-29b-3p (and other miRNAs w/seed AGCACCA),miR-501-3p (and other miRNAs w/seed AUGCACC)
Skeletal and Muscular Disorders	2.59E-04-4.92E-02	5	miR-99b-5p, miR-212-3p, miR-181d-5p, miR-29c-3p, miR-501-3p	miR-100-5p (and other miRNAs w/seed ACCCGUA),miR-132-3p (and other miRNAs w/seed AACAGUC),miR-181a-5p (and other miRNAs w/seed ACAUUCA),miR-29b-3p (and other miRNAs w/seed AGCACCA),miR-501-3p (and other miRNAs w/seed AUGCACC)
Inflammatory Response	4.16E-04-4.56E-02	5	miR-99b-5p, miR-143-3p, miR-150-5p, miR-330-3p, miR-941	miR-100-5p (and other miRNAs w/seed ACCCGUA),miR-143-3p (and other miRNAs w/seed GAGAUGA),miR-150-5p (and other miRNAs w/seed CUCCCAA),miR-330-3p (and other miRNAs w/seed CAAAGCA),miR-941 (miRNAs w/seed ACCCGGC)
Digestive System Development and Function	8.84E-04-8.84E-04	2	miR-99b-5p, miR-143-3p,	miR-100-5p (and other miRNAs w/seed ACCCGUA),miR-143-3p (and other miRNAs w/seed GAGAUGA)
Hepatic System Development and Function	8.84E-04-8.84E-04	2	miR-99b-5p, miR-143-3p,	miR-100-5p (and other miRNAs w/seed ACCCGUA),miR-143-3p (and other miRNAs w/seed GAGAUGA)
Hepatic System Disease	8.84E-04-1.52E-02	2	miR-99b-5p, miR-143-3p,	miR-100-5p (and other miRNAs w/seed ACCCGUA),miR-143-3p (and other miRNAs w/seed GAGAUGA)
Infectious Diseases	8.84E-04-8.84E-04	2	miR-99b-5p, miR-143-3p,	miR-100-5p (and other miRNAs w/seed ACCCGUA),miR-143-3p (and other miRNAs w/seed GAGAUGA)

Inflammatory Disease	8.84E-04-4.56E-02	7	miR-99b-5p, miR-143-3p, miR-150-5p, miR-181d-5p, miR-29c-3p, miR-330-3p, miR-941	miR-100-5p (and other miRNAs w/seed ACCCGUA),miR-143-3p (and other miRNAs w/seed GAGAUGA),miR-150-5p (and other miRNAs w/seed CUCCCAA),miR-181a-5p (and other miRNAs w/seed ACAUUCA),miR-29b-3p (and other miRNAs w/seed AGCACCA),miR-330-3p (and other miRNAs w/seed CAAAGCA),miR-941 (miRNAs w/seed ACCCGGC)
Organ Development	8.84E-04-8.84E-04	2	miR-99b-5p, miR-143-3p	miR-100-5p (and other miRNAs w/seed ACCCGUA),miR-143-3p (and other miRNAs w/seed GAGAUGA)
Metabolic Disease	1.1E-03-2.67E-02	6	miR-99b-5p, miR-212-3p, miR-143-3p, miR-150-5p, miR-181d-5p, miR-29c-3p	miR-100-5p (and other miRNAs w/seed ACCCGUA),miR-132-3p (and other miRNAs w/seed AACAGUC),miR-143-3p (and other miRNAs w/seed GAGAUGA),miR-150-5p (and other miRNAs w/seed CUCCCAA),miR-181a-5p (and other miRNAs w/seed ACAUUCA),miR-29b-3p (and other miRNAs w/seed AGCACCA)
Connective Tissue Disorders	1.31E-03-4.92E-02	3	miR-143-3p, miR-181d-5p, miR-29c-3p	miR-143-3p (and other miRNAs w/seed GAGAUGA),miR-181a-5p (and other miRNAs w/seed ACAUUCA),miR-29b-3p (and other miRNAs w/seed AGCACCA)
Cell Death and Survival	1.5E-03-4.68E-02	5	miR-99b-5p, miR-143-3p, miR-150-5p, miR-181d-5p, miR-29c-3p	miR-100-5p (and other miRNAs w/seed ACCCGUA),miR-143-3p (and other miRNAs w/seed GAGAUGA),miR-150-5p (and other miRNAs w/seed CUCCCAA),miR-181a-5p (and other miRNAs w/seed ACAUUCA),miR-29b-3p (and other miRNAs w/seed AGCACCA)
Cell Morphology	1.5E-03-1.5E-03	1	miR-143-3p	miR-143-3p (and other miRNAs w/seed GAGAUGA)

Supplementary Table 8 Top-5 miRNA molecular and cellular functions

Name	p-value range	# Molecules	miRNA
Cell Death and Survival	4.68E-02 - 1.50E-03	5	miR-143-3p, miR-181d-5p **(miR-181a-5p)**, miR-150-5p, miR-99b-5p **(miR-100-5p)**, miR-29c-3p **(miR-29b-3p)**
Cell Morphology	1.50E-03 - 1.50E-03	1	miR-143-3p
Cellular Function and Maintenance	6.75E-03 - 1.50E-03	2	miR-143-3p, miR-29c-3p **(miR-29b-3p)**
Cellular Movement	4.71E-02 - 3.00E-03	3	miR-143-3p, miR-181d-5p **(miR-181a-5p)**, miR-29c-3p **(miR-29b-3p)**
Cell Cycle	8.99E-03 - 3.75E-03	2	miR-212-3p **(miR-132-3p)**, miR-99b-5p **(miR-100-5p)**

Oasis miRNA nomenclature (non-bold)**, IPA miRNA nomenclature (bold)**

Supplementary Table 9 miRNA-mRNA targets

ID (OASIS)	Symbol (IPA)	Expr Fold Change	Source	Confidence	ID	Symbol	Expr p-value	Expr Fold Change
hsa-let-7g-3p	let-7a-2-3p (and other miRNAs w/seed UGUACAG)	1.44	TargetScan Human	Moderate (predicted)	ACTR10	ACTR10	9.61E-03	-1.22
hsa-let-7g-3p	let-7a-2-3p (and other miRNAs w/seed UGUACAG)	1.44	TargetScan Human	Moderate (predicted)	ALDH9A1	ALDH9A1	2.51E-02	-1.22
hsa-let-7g-3p	let-7a-2-3p (and other miRNAs w/seed UGUACAG)	1.44	TargetScan Human	Moderate (predicted)	USMG5	ATP5MK	3.24E-02	-1.21
hsa-let-7g-3p	let-7a-2-3p (and other miRNAs w/seed UGUACAG)	1.44	TargetScan Human	High (predicted)	BAZ1A	BAZ1A	8.11E-03	-1.23
hsa-let-7g-3p	let-7a-2-3p (and other miRNAs w/seed UGUACAG)	1.44	TargetScan Human	Moderate (predicted)	C1orf112	C1orf112	3.51E-02	-1.22
hsa-let-7g-3p	let-7a-2-3p (and other miRNAs w/seed UGUACAG)	1.44	TargetScan Human	Moderate (predicted)	CCDC125	CCDC125	6.95E-03	-1.21
hsa-let-7g-3p	let-7a-2-3p (and other miRNAs w/seed UGUACAG)	1.44	TargetScan Human	Moderate (predicted)	CCDC186	CCDC186	1.29E-02	-1.27
hsa-let-7g-3p	let-7a-2-3p (and other miRNAs w/seed UGUACAG)	1.44	TargetScan Human	Moderate (predicted)	CCNB1	CCNB1	2.33E-02	-1.24
hsa-let-7g-3p	let-7a-2-3p (and other miRNAs w/seed UGUACAG)	1.44	TargetScan Human	Moderate (predicted)	CDK8	CDK8	2.81E-03	-1.29
hsa-let-7g-3p	let-7a-2-3p (and other miRNAs w/seed UGUACAG)	1.44	TargetScan Human	Moderate (predicted)	CKS2	CKS2	1.36E-02	-1.39
hsa-let-7g-3p	let-7a-2-3p (and other miRNAs w/seed UGUACAG)	1.44	TargetScan Human	Moderate (predicted)	CPNE8	CPNE8	1.13E-02	-1.26
hsa-let-7g-3p	let-7a-2-3p (and other miRNAs w/seed UGUACAG)	1.44	TargetScan Human	Moderate (predicted)	DHX36	DHX36	1.44E-02	-1.23
hsa-let-7g-3p	let-7a-2-3p (and other miRNAs w/seed UGUACAG)	1.44	TargetScan Human	Moderate (predicted)	DYNLT3	DYNLT3	4.97E-02	-1.24
hsa-let-7g-3p	let-7a-2-3p (and other miRNAs w/seed UGUACAG)	1.44	TargetScan Human	Moderate (predicted)	EIF4E	EIF4E	4.78E-02	-1.30
hsa-let-7g-3p	let-7a-2-3p (and other miRNAs w/seed UGUACAG)	1.44	TargetScan Human	Moderate (predicted)	HMGB2	HMGB2	1.51E-02	-1.22
hsa-let-7g-3p	let-7a-2-3p (and other miRNAs w/seed UGUACAG)	1.44	TargetScan Human	Moderate (predicted)	IFIT2	IFIT2	3.91E-02	-1.30
hsa-let-7g-3p	let-7a-2-3p (and other miRNAs w/seed UGUACAG)	1.44	TargetScan Human	Moderate (predicted)	KCND2	KCND2	3.18E-02	-1.27
hsa-let-7g-3p	let-7a-2-3p (and other miRNAs w/seed UGUACAG)	1.44	TargetScan Human	Moderate (predicted)	MAK16	MAK16	1.21E-02	-1.25
hsa-let-7g-3p	let-7a-2-3p (and other miRNAs w/seed UGUACAG)	1.44	TargetScan Human	Moderate (predicted)	MND1	MND1	4.18E-02	-1.27
hsa-let-7g-3p	let-7a-2-3p (and other miRNAs w/seed UGUACAG)	1.44	TargetScan Human	Moderate (predicted)	NUP133	NUP133	9.71E-03	-1.25
hsa-let-7g-3p	let-7a-2-3p (and other miRNAs w/seed UGUACAG)	1.44	TargetScan Human	Moderate (predicted)	PBX3	PBX3	2.21E-02	-1.21
hsa-let-7g-3p	let-7a-2-3p (and other miRNAs w/seed UGUACAG)	1.44	TargetScan Human	Moderate (predicted)	PHF6	PHF6	6.76E-03	-1.27
hsa-let-7g-3p	let-7a-2-3p (and other miRNAs w/seed UGUACAG)	1.44	TargetScan Human	Moderate (predicted)	REEP3	REEP3	2.93E-02	-1.28
hsa-let-7g-3p	let-7a-2-3p (and other miRNAs w/seed UGUACAG)	1.44	TargetScan Human	Moderate (predicted)	REV3L	REV3L	1.94E-02	-1.21
hsa-let-7g-3p	let-7a-2-3p (and other miRNAs w/seed UGUACAG)	1.44	TargetScan Human	Moderate (predicted)	ACN9	SDHAF3	2.85E-02	-1.24
hsa-let-7g-3p	let-7a-2-3p (and other miRNAs w/seed UGUACAG)	1.44	TargetScan Human	Moderate (predicted)	SMC3	SMC3	2.96E-02	-1.22
hsa-let-7g-3p	let-7a-2-3p (and other miRNAs w/seed UGUACAG)	1.44	TargetScan Human	High (predicted)	TPMT	TPMT	2.54E-02	-1.45
hsa-let-7g-3p	let-7a-2-3p (and other miRNAs w/seed UGUACAG)	1.44	TargetScan Human	Moderate (predicted)	TRAPPC13	TRAPPC13	1.80E-02	-1.22
hsa-let-7g-3p	let-7a-2-3p (and other miRNAs w/seed UGUACAG)	1.44	TargetScan Human	Moderate (predicted)	TSNAX	TSNAX	3.23E-02	-1.23
hsa-let-7g-3p	let-7a-2-3p (and other miRNAs w/seed UGUACAG)	1.44	TargetScan Human	Moderate (predicted)	VRK1	VRK1	2.90E-02	-1.23
hsa-let-7g-3p	let-7a-2-3p (and other miRNAs w/seed UGUACAG)	1.44	TargetScan Human	Moderate (predicted)	ZNF100	ZNF100	3.92E-02	-1.23
hsa-let-7g-3p	let-7a-2-3p (and other miRNAs w/seed UGUACAG)	1.44	TargetScan Human	High (predicted)	ZNF143	ZNF143	1.12E-03	-1.27
hsa-let-7g-3p	let-7a-2-3p (and other miRNAs w/seed UGUACAG)	1.44	TargetScan Human	High (predicted)	ZNF627	ZNF627	3.71E-02	-1.21
hsa-let-7g-3p	let-7a-2-3p (and other miRNAs w/seed UGUACAG)	1.44	TargetScan Human	Moderate (predicted)	ZNF675	ZNF675	3.08E-02	-1.36
hsa-miR-4508	miR-12197-3p (and other miRNAs w/seed CGGGGCU)	1.74	TargetScan Human	Moderate (predicted)	C11orf73	HIKESHI	2.98E-02	-1.21
hsa-miR-4508	miR-12197-3p (and other miRNAs w/seed CGGGGCU)	1.74	TargetScan Human	Moderate (predicted)	MAPK8	MAPK8	4.82E-02	-1.22
hsa-miR-4508	miR-12197-3p (and other miRNAs w/seed CGGGGCU)	1.74	TargetScan Human	Moderate (predicted)	MRS2	MRS2	2.00E-02	-1.20
hsa-miR-4508	miR-12197-3p (and other miRNAs w/seed CGGGGCU)	1.74	TargetScan Human	Moderate (predicted)	RALGAPA2	RALGAPA2	4.18E-02	-1.22
hsa-miR-4508	miR-12197-3p (and other miRNAs w/seed CGGGGCU)	1.74	TargetScan Human	Moderate (predicted)	SAMHD1	SAMHD1	1.55E-02	-1.35
hsa-miR-4508	miR-12197-3p (and other miRNAs w/seed CGGGGCU)	1.74	TargetScan Human	Moderate (predicted)	UTP6	UTP6	6.72E-03	-1.21
hsa-miR-4488	miR-1237-5p (and other miRNAs w/seed GGGGGCG)	1.80	TargetScan Human	High (predicted)	LRRC16A	CARMIL1	2.56E-02	-1.33
hsa-miR-4488	miR-1237-5p (and other miRNAs w/seed GGGGGCG)	1.80	TargetScan Human	Moderate (predicted)	CLDN12	CLDN12	3.27E-02	-1.22
hsa-miR-4488	miR-1237-5p (and other miRNAs w/seed GGGGGCG)	1.80	TargetScan Human	Moderate (predicted)	COTL1	COTL1	4.54E-02	-1.21
hsa-miR-4488	miR-1237-5p (and other miRNAs w/seed GGGGGCG)	1.80	TargetScan Human	Moderate (predicted)	CTNNA1	CTNNA1	3.16E-03	-1.20
hsa-miR-4488	miR-1237-5p (and other miRNAs w/seed GGGGGCG)	1.80	TargetScan Human	Moderate (predicted)	FAM98A	FAM98A	1.91E-02	-1.22
hsa-miR-4488	miR-1237-5p (and other miRNAs w/seed GGGGGCG)	1.80	TargetScan Human	Moderate (predicted)	HAUS2	HAUS2	1.03E-02	-1.21
hsa-miR-4488	miR-1237-5p (and other miRNAs w/seed GGGGGCG)	1.80	TargetScan Human	Moderate (predicted)	KPNA6	KPNA6	3.28E-02	-1.20
hsa-miR-4488	miR-1237-5p (and other miRNAs w/seed GGGGGCG)	1.80	TargetScan Human	Moderate (predicted)	LIMS1	LIMS1	6.81E-03	-1.34
hsa-miR-4488	miR-1237-5p (and other miRNAs w/seed GGGGGCG)	1.80	TargetScan Human	Moderate (predicted)	LSG1	LSG1	3.12E-02	-1.21
hsa-miR-4488	miR-1237-5p (and other miRNAs w/seed GGGGGCG)	1.80	TargetScan Human	Moderate (predicted)	MGME1	MGME1	9.30E-04	-1.21
hsa-miR-4488	miR-1237-5p (and other miRNAs w/seed GGGGGCG)	1.80	TargetScan Human	Moderate (predicted)	MOCS2	MOCS2	3.61E-02	-1.22
hsa-miR-4488	miR-1237-5p (and other miRNAs w/seed GGGGGCG)	1.80	TargetScan Human	Moderate (predicted)	MYO6	MYO6	1.62E-02	-1.30
hsa-miR-4488	miR-1237-5p (and other miRNAs w/seed GGGGGCG)	1.80	TargetScan Human	Moderate (predicted)	PBX2	PBX2	3.61E-02	-1.23
hsa-miR-4488	miR-1237-5p (and other miRNAs w/seed GGGGGCG)	1.80	TargetScan Human	Moderate (predicted)	POLR3F	POLR3F	4.48E-03	-1.23
hsa-miR-4488	miR-1237-5p (and other miRNAs w/seed GGGGGCG)	1.80	TargetScan Human	Moderate (predicted)	RBM7	RBM7	2.83E-02	-1.33
hsa-miR-4488	miR-1237-5p (and other miRNAs w/seed GGGGGCG)	1.80	TargetScan Human	Moderate (predicted)	RNF125	RNF125	3.34E-02	-1.30
hsa-miR-4488	miR-1237-5p (and other miRNAs w/seed GGGGGCG)	1.80	TargetScan Human	Moderate (predicted)	RRM2	RRM2	4.67E-02	-1.23
hsa-miR-4488	miR-1237-5p (and other miRNAs w/seed GGGGGCG)	1.80	TargetScan Human	Moderate (predicted)	RRP36	RRP36	8.66E-03	-1.21
hsa-miR-4488	miR-1237-5p (and other miRNAs w/seed GGGGGCG)	1.80	TargetScan Human	High (predicted)	TGM2	TGM2	1.07E-02	-1.25
hsa-miR-4488	miR-1237-5p (and other miRNAs w/seed GGGGGCG)	1.80	TargetScan Human	Moderate (predicted)	ZFP90	ZFP90	3.01E-02	-1.20
hsa-miR-4488	miR-1237-5p (and other miRNAs w/seed GGGGGCG)	1.80	TargetScan Human	Moderate (predicted)	ZNF641	ZNF641	8.80E-03	-1.22
hsa-miR-1260b	miR-1260a (and other miRNAs w/seed UCCCACC)	1.29	TargetScan Human	Moderate (predicted)	BZW1	BZW1	1.22E-02	-1.41
hsa-miR-1260b	miR-1260a (and other miRNAs w/seed UCCCACC)	1.29	TargetScan Human	Moderate (predicted)	CCR6	CCR6	3.13E-02	-1.37
hsa-miR-1260b	miR-1260a (and other miRNAs w/seed UCCCACC)	1.29	TargetScan Human	Moderate (predicted)	CNKSR2	CNKSR2	1.10E-02	-1.30
hsa-miR-1260b	miR-1260a (and other miRNAs w/seed UCCCACC)	1.29	TargetScan Human	Moderate (predicted)	DEK	DEK	1.42E-02	-1.26
hsa-miR-1260b	miR-1260a (and other miRNAs w/seed UCCCACC)	1.29	TargetScan Human	High (predicted)	DHX29	DHX29	2.90E-02	-1.22
hsa-miR-1260b	miR-1260a (and other miRNAs w/seed UCCCACC)	1.29	TargetScan Human	Moderate (predicted)	FAM111B	FAM111B	7.74E-03	-1.35
hsa-miR-1260b	miR-1260a (and other miRNAs w/seed UCCCACC)	1.29	TargetScan Human	Moderate (predicted)	GNPNAT1	GNPNAT1	8.00E-03	-1.25
hsa-miR-1260b	miR-1260a (and other miRNAs w/seed UCCCACC)	1.29	TargetScan Human	High (predicted)	HAUS2	HAUS2	1.03E-02	-1.21
hsa-miR-1260b	miR-1260a (and other miRNAs w/seed UCCCACC)	1.29	TargetScan Human	Moderate (predicted)	IL6ST	IL6ST	4.14E-02	-1.22
hsa-miR-1260b	miR-1260a (and other miRNAs w/seed UCCCACC)	1.29	TargetScan Human	Moderate (predicted)	KIF3A	KIF3A	3.17E-02	-1.26
hsa-miR-1260b	miR-1260a (and other miRNAs w/seed UCCCACC)	1.29	TargetScan Human	Moderate (predicted)	KPNA6	KPNA6	3.28E-02	-1.20
hsa-miR-1260b	miR-1260a (and other miRNAs w/seed UCCCACC)	1.29	TargetScan Human	Moderate (predicted)	LIN7C	LIN7C	1.55E-02	-1.21
hsa-miR-1260b	miR-1260a (and other miRNAs w/seed UCCCACC)	1.29	TargetScan Human	Moderate (predicted)	MOB1A	MOB1A	2.46E-02	-1.21
hsa-miR-1260b	miR-1260a (and other miRNAs w/seed UCCCACC)	1.29	TargetScan Human	High (predicted)	PLEKHF2	PLEKHF2	2.57E-02	-1.24
hsa-miR-1260b	miR-1260a (and other miRNAs w/seed UCCCACC)	1.29	TargetScan Human	Moderate (predicted)	PSMA4	PSMA4	2.34E-02	-1.27
hsa-miR-1260b	miR-1260a (and other miRNAs w/seed UCCCACC)	1.29	TargetScan Human	Moderate (predicted)	RAB9A	RAB9A	7.97E-03	-1.22
hsa-miR-1260b	miR-1260a (and other miRNAs w/seed UCCCACC)	1.29	TargetScan Human	Moderate (predicted)	RHOBTB3	RHOBTB3	3.12E-02	-1.24
hsa-miR-1260b	miR-1260a (and other miRNAs w/seed UCCCACC)	1.29	TargetScan Human	Moderate (predicted)	RPL31	RPL31	4.47E-02	-1.28
hsa-miR-1260b	miR-1260a (and other miRNAs w/seed UCCCACC)	1.29	TargetScan Human	High (predicted)	RRM2	RRM2	4.67E-02	-1.23
hsa-miR-1260b	miR-1260a (and other miRNAs w/seed UCCCACC)	1.29	TargetScan Human	Moderate (predicted)	SKAP2	SKAP2	8.20E-03	-1.24
hsa-miR-1260b	miR-1260a (and other miRNAs w/seed UCCCACC)	1.29	TargetScan Human	Moderate (predicted)	SP1	SP1	2.88E-03	-1.20
hsa-miR-1260b	miR-1260a (and other miRNAs w/seed UCCCACC)	1.29	TargetScan Human	Moderate (predicted)	ZFP69B	ZFP69B	1.69E-02	-1.21
hsa-miR-1260b	miR-1260a (and other miRNAs w/seed UCCCACC)	1.29	TargetScan Human	High (predicted)	ZNF146	ZNF146	4.67E-02	-1.21
hsa-miR-1260b	miR-1260a (and other miRNAs w/seed UCCCACC)	1.29	TargetScan Human	High (predicted)	ZNF561	ZNF561	1.47E-03	-1.25
hsa-miR-1260b	miR-1260a (and other miRNAs w/seed UCCCACC)	1.29	TargetScan Human	High (predicted)	ZNF569	ZNF569	2.28E-03	-1.20
hsa-miR-1260b	miR-1260a (and other miRNAs w/seed UCCCACC)	1.29	TargetScan Human	Moderate (predicted)	ZNF615	ZNF615	1.69E-03	-1.31
hsa-miR-1260b	miR-1260a (and other miRNAs w/seed UCCCACC)	1.29	TargetScan Human	High (predicted)	ZNF627	ZNF627	3.71E-02	-1.21
hsa-miR-143-3p	miR-143-3p (and other miRNAs w/seed GAGAUGA)	-1.58	TargetScan Human	Moderate (predicted)	FOXD4L6	FOXD4L3/FOXD4L6	1.65E-02	1.32
hsa-miR-143-3p	miR-143-3p (and other miRNAs w/seed GAGAUGA)	-1.58	TargetScan Human	Moderate (predicted)	SPINT3	SPINT3	3.15E-03	1.33
hsa-miR-150-5p	miR-150-5p (and other miRNAs w/seed CUCCCAA)	1.53	TargetScan Human	Moderate (predicted)	CAST	CAST	2.79E-04	-1.29
hsa-miR-150-5p	miR-150-5p (and other miRNAs w/seed CUCCCAA)	1.53	TargetScan Human	Moderate (predicted)	FOPNL	CEP20	4.87E-02	-1.20
hsa-miR-150-5p	miR-150-5p (and other miRNAs w/seed CUCCCAA)	1.53	TargetScan Human	Moderate (predicted)	DUSP10	DUSP10	1.32E-02	-1.25
hsa-miR-150-5p	miR-150-5p (and other miRNAs w/seed CUCCCAA)	1.53	TargetScan Human	Moderate (predicted)	DYNLT3	DYNLT3	4.97E-02	-1.24
hsa-miR-150-5p	miR-150-5p (and other miRNAs w/seed CUCCCAA)	1.53	TargetScan Human	Moderate (predicted)	GMFB	GMFB	3.70E-02	-1.21
hsa-miR-150-5p	miR-150-5p (and other miRNAs w/seed CUCCCAA)	1.53	TargetScan Human	Moderate (predicted)	IFIT5	IFIT5	1.48E-02	-1.25
hsa-miR-150-5p	miR-150-5p (and other miRNAs w/seed CUCCCAA)	1.53	TargetScan Human	High (predicted)	NAF1	NAF1	1.43E-02	-1.24
hsa-miR-150-5p	miR-150-5p (and other miRNAs w/seed CUCCCAA)	1.53	TargetScan Human	Moderate (predicted)	SLC10A5	SLC10A5	1.11E-02	-1.23
hsa-miR-150-5p	miR-150-5p (and other miRNAs w/seed CUCCCAA)	1.53	TargetScan Human	Moderate (predicted)	SMC3	SMC3	2.96E-02	-1.22
hsa-miR-150-5p	miR-150-5p (and other miRNAs w/seed CUCCCAA)	1.53	TargetScan Human	Moderate (predicted)	TP53	TP53	1.54E-02	-1.21
hsa-miR-150-5p	miR-150-5p (and other miRNAs w/seed CUCCCAA)	1.53	TargetScan Human	Moderate (predicted)	TRAPPC2	TRAPPC2	2.36E-02	-1.37
hsa-miR-24-2-5p	miR-24-1-5p (and other miRNAs w/seed GCCUACU)	1.32	TargetScan Human	High (predicted)	BTK	BTK	9.36E-03	-1.20
hsa-miR-24-2-5p	miR-24-1-5p (and other miRNAs w/seed GCCUACU)	1.32	TargetScan Human	High (predicted)	C2orf49	C2orf49	8.35E-03	-1.21
hsa-miR-24-2-5p	miR-24-1-5p (and other miRNAs w/seed GCCUACU)	1.32	TargetScan Human	Moderate (predicted)	C5orf51	C5orf51	4.12E-03	-1.29
hsa-miR-24-2-5p	miR-24-1-5p (and other miRNAs w/seed GCCUACU)	1.32	TargetScan Human	Moderate (predicted)	CCDC186	CCDC186	1.29E-02	-1.27
hsa-miR-24-2-5p	miR-24-1-5p (and other miRNAs w/seed GCCUACU)	1.32	TargetScan Human	High (predicted)	COQ10B	COQ10B	2.06E-02	-1.21
hsa-miR-24-2-5p	miR-24-1-5p (and other miRNAs w/seed GCCUACU)	1.32	TargetScan Human	Moderate (predicted)	CUL5	CUL5	2.73E-02	-1.21
hsa-miR-24-2-5p	miR-24-1-5p (and other miRNAs w/seed GCCUACU)	1.32	TargetScan Human	Moderate (predicted)	DDX21	DDX21	1.17E-02	-1.21
hsa-miR-24-2-5p	miR-24-1-5p (and other miRNAs w/seed GCCUACU)	1.32	TargetScan Human	Moderate (predicted)	GAS2L3	GAS2L3	3.20E-02	-1.30
hsa-miR-24-2-5p	miR-24-1-5p (and other miRNAs w/seed GCCUACU)	1.32	TargetScan Human	High (predicted)	ICE2	ICE2	1.75E-02	-1.20
hsa-miR-24-2-5p	miR-24-1-5p (and other miRNAs w/seed GCCUACU)	1.32	TargetScan Human	Moderate (predicted)	IDI1	IDI1	1.62E-02	-1.22
hsa-miR-24-2-5p	miR-24-1-5p (and other miRNAs w/seed GCCUACU)	1.32	TargetScan Human	High (predicted)	IL6ST	IL6ST	4.14E-02	-1.22
hsa-miR-24-2-5p	miR-24-1-5p (and other miRNAs w/seed GCCUACU)	1.32	TargetScan Human	Moderate (predicted)	SERBP1	SERBP1	7.08E-04	-1.26
hsa-miR-24-2-5p	miR-24-1-5p (and other miRNAs w/seed GCCUACU)	1.32	TargetScan Human	Moderate (predicted)	SLC4A7	SLC4A7	2.30E-02	-1.22
hsa-miR-24-2-5p	miR-24-1-5p (and other miRNAs w/seed GCCUACU)	1.32	TargetScan Human	Moderate (predicted)	SLMAP	SLMAP	5.97E-03	-1.22
hsa-miR-24-2-5p	miR-24-1-5p (and other miRNAs w/seed GCCUACU)	1.32	TargetScan Human	Moderate (predicted)	TRAPPC13	TRAPPC13	1.80E-02	-1.22
hsa-miR-24-2-5p	miR-24-1-5p (and other miRNAs w/seed GCCUACU)	1.32	TargetScan Human	Moderate (predicted)	VPS26A	VPS26A	4.30E-02	-1.21
hsa-miR-24-2-5p	miR-24-1-5p (and other miRNAs w/seed GCCUACU)	1.32	TargetScan Human	Moderate (predicted)	ZMYM4	ZMYM4	6.39E-03	-1.26
hsa-miR-24-2-5p	miR-24-1-5p (and other miRNAs w/seed GCCUACU)	1.32	TargetScan Human	Moderate (predicted)	ZNF148	ZNF148	2.53E-02	-1.21
hsa-miR-616-5p	miR-292b-5p (and other miRNAs w/seed CUCAAAA)	1.31	TargetScan Human	Moderate (predicted)	ARPP19	ARPP19	8.99E-03	-1.24
hsa-miR-616-5p	miR-292b-5p (and other miRNAs w/seed CUCAAAA)	1.31	TargetScan Human	Moderate (predicted)	CAPZA2	CAPZA2	1.65E-02	-1.22
hsa-miR-616-5p	miR-292b-5p (and other miRNAs w/seed CUCAAAA)	1.31	TargetScan Human	Moderate (predicted)	CETN2	CETN2	2.28E-02	-1.20
hsa-miR-616-5p	miR-292b-5p (and other miRNAs w/seed CUCAAAA)	1.31	TargetScan Human	Moderate (predicted)	COMMD8	COMMD8	4.13E-02	-1.21
hsa-miR-616-5p	miR-292b-5p (and other miRNAs w/seed CUCAAAA)	1.31	TargetScan Human	Moderate (predicted)	DNAAF2	DNAAF2	1.20E-02	-1.23
hsa-miR-616-5p	miR-292b-5p (and other miRNAs w/seed CUCAAAA)	1.31	TargetScan Human	Moderate (predicted)	GIMAP2	GIMAP2	1.30E-02	-1.29
hsa-miR-616-5p	miR-292b-5p (and other miRNAs w/seed CUCAAAA)	1.31	TargetScan Human	Moderate (predicted)	HLTF	HLTF	1.11E-02	-1.27
hsa-miR-616-5p	miR-292b-5p (and other miRNAs w/seed CUCAAAA)	1.31	TargetScan Human	Moderate (predicted)	KRR1	KRR1	1.58E-02	-1.23
hsa-miR-616-5p	miR-292b-5p (and other miRNAs w/seed CUCAAAA)	1.31	TargetScan Human	Moderate (predicted)	LYRM7	LYRM7	4.89E-03	-1.43

hsa-miR-616-5p	miR-292b-5p (and other miRNAs w/seed CUCAAAA)	1.31	TargetScan Human	Moderate (predicted)	MOB1A	MOB1A	2.46E-02	-1.21
hsa-miR-616-5p	miR-292b-5p (and other miRNAs w/seed CUCAAAA)	1.31	TargetScan Human	Moderate (predicted)	MRPL50	MRPL50	3.88E-03	-1.24
hsa-miR-616-5p	miR-292b-5p (and other miRNAs w/seed CUCAAAA)	1.31	TargetScan Human	Moderate (predicted)	PLA1A	PLA1A	3.79E-02	-1.29
hsa-miR-616-5p	miR-292b-5p (and other miRNAs w/seed CUCAAAA)	1.31	TargetScan Human	Moderate (predicted)	PLSCR1	PLSCR1	8.31E-03	-1.32
hsa-miR-616-5p	miR-292b-5p (and other miRNAs w/seed CUCAAAA)	1.31	TargetScan Human	High (predicted)	RECQL	RECQL	8.56E-03	-1.25
hsa-miR-616-5p	miR-292b-5p (and other miRNAs w/seed CUCAAAA)	1.31	TargetScan Human	Moderate (predicted)	ROCK1	ROCK1	1.87E-02	-1.26
hsa-miR-616-5p	miR-292b-5p (and other miRNAs w/seed CUCAAAA)	1.31	TargetScan Human	Moderate (predicted)	RSF1	RSF1	2.87E-02	-1.23
hsa-miR-616-5p	miR-292b-5p (and other miRNAs w/seed CUCAAAA)	1.31	TargetScan Human	Moderate (predicted)	SCAF11	SCAF11	9.28E-03	-1.21
hsa-miR-616-5p	miR-292b-5p (and other miRNAs w/seed CUCAAAA)	1.31	TargetScan Human	Moderate (predicted)	SENP6	SENP6	7.96E-03	-1.25
hsa-miR-616-5p	miR-292b-5p (and other miRNAs w/seed CUCAAAA)	1.31	TargetScan Human	Moderate (predicted)	SKA1	SKA1	4.51E-02	-1.30
hsa-miR-616-5p	miR-292b-5p (and other miRNAs w/seed CUCAAAA)	1.31	TargetScan Human	Moderate (predicted)	SQLE	SQLE	2.31E-02	-1.21
hsa-miR-616-5p	miR-292b-5p (and other miRNAs w/seed CUCAAAA)	1.31	TargetScan Human	Moderate (predicted)	TIFA	TIFA	1.15E-02	-1.25
hsa-miR-616-5p	miR-292b-5p (and other miRNAs w/seed CUCAAAA)	1.31	TargetScan Human	Moderate (predicted)	TPMT	TPMT	2.54E-02	-1.45
hsa-miR-616-5p	miR-292b-5p (and other miRNAs w/seed CUCAAAA)	1.31	TargetScan Human	High (predicted)	TRAPPC2	TRAPPC2	2.36E-02	-1.37
hsa-miR-616-5p	miR-292b-5p (and other miRNAs w/seed CUCAAAA)	1.31	TargetScan Human	High (predicted)	TSNAX	TSNAX	3.23E-02	-1.23
hsa-miR-616-5p	miR-292b-5p (and other miRNAs w/seed CUCAAAA)	1.31	TargetScan Human	Moderate (predicted)	UBE2V2	UBE2V2	4.99E-03	-1.25
hsa-miR-616-5p	miR-292b-5p (and other miRNAs w/seed CUCAAAA)	1.31	TargetScan Human	Moderate (predicted)	UTP20	UTP20	1.65E-02	-1.24
hsa-miR-616-5p	miR-292b-5p (and other miRNAs w/seed CUCAAAA)	1.31	TargetScan Human	High (predicted)	ZFP69B	ZFP69B	1.69E-02	-1.21
hsa-miR-616-5p	miR-292b-5p (and other miRNAs w/seed CUCAAAA)	1.31	TargetScan Human	Moderate (predicted)	ZNF678	ZNF678	8.69E-03	-1.23
hsa-miR-29c-3p	miR-29b-3p (and other miRNAs w/seed AGCACCA)	1.27	TargetScan Human	Moderate (predicted)	ABCE1	ABCE1	1.63E-02	-1.28
hsa-miR-29c-3p	miR-29b-3p (and other miRNAs w/seed AGCACCA)	1.27	TargetScan Human	Moderate (predicted)	ACTR10	ACTR10	9.61E-03	-1.22
hsa-miR-29c-3p	miR-29b-3p (and other miRNAs w/seed AGCACCA)	1.27	TargetScan Human	Moderate (predicted)	ANXA5	ANXA5	1.99E-02	-1.26
hsa-miR-29c-3p	miR-29b-3p (and other miRNAs w/seed AGCACCA)	1.27	TargetScan Human	High (predicted)	ATAD2B	ATAD2B	2.42E-02	-1.27
hsa-miR-29c-3p	miR-29b-3p (and other miRNAs w/seed AGCACCA)	1.27	TargetScan Human	Moderate (predicted)	CEP97	CEP97	2.66E-02	-1.23
hsa-miR-29c-3p	miR-29b-3p (and other miRNAs w/seed AGCACCA)	1.27	TargetScan Human	Moderate (predicted)	DLG1	DLG1	2.06E-02	-1.21
hsa-miR-29c-3p	miR-29b-3p (and other miRNAs w/seed AGCACCA)	1.27	TargetScan Human	Moderate (predicted)	DPP8	DPP8	2.32E-02	-1.20
hsa-miR-29c-3p	miR-29b-3p (and other miRNAs w/seed AGCACCA)	1.27	TargetScan Human	Moderate (predicted)	EIF3J	EIF3J	1.22E-02	-1.24
hsa-miR-29c-3p	miR-29b-3p (and other miRNAs w/seed AGCACCA)	1.27	TargetScan Human	Moderate (predicted)	FZD5	FZD5	2.39E-02	-1.23
hsa-miR-29c-3p	miR-29b-3p (and other miRNAs w/seed AGCACCA)	1.27	Ingenuity Expert Findings	Experimentally Observed	GMFB	GMFB	3.70E-02	-1.21
hsa-miR-29c-3p	miR-29b-3p (and other miRNAs w/seed AGCACCA)	1.27	TargetScan Human	Moderate (predicted)	HMGCS1	HMGCS1	5.39E-03	-1.31
hsa-miR-29c-3p	miR-29b-3p (and other miRNAs w/seed AGCACCA)	1.27	TargetScan Human	Moderate (predicted)	LYPLA1	LYPLA1	2.94E-02	-1.46
hsa-miR-29c-3p	miR-29b-3p (and other miRNAs w/seed AGCACCA)	1.27	TargetScan Human	Moderate (predicted)	LYRM7	LYRM7	4.89E-03	-1.43
hsa-miR-29c-3p	miR-29b-3p (and other miRNAs w/seed AGCACCA)	1.27	TargetScan Human	Moderate (predicted)	METAP2	METAP2	2.09E-02	-1.21
hsa-miR-29c-3p	miR-29b-3p (and other miRNAs w/seed AGCACCA)	1.27	TargetScan Human	High (predicted)	MFAP3	MFAP3	4.12E-02	-1.22
hsa-miR-29c-3p	miR-29b-3p (and other miRNAs w/seed AGCACCA)	1.27	TargetScan Human	Moderate (predicted)	MOB1A	MOB1A	2.46E-02	-1.21
hsa-miR-29c-3p	miR-29b-3p (and other miRNAs w/seed AGCACCA)	1.27	TargetScan Human	High (predicted)	NIPSNAP3A	NIPSNAP3A	1.51E-03	-1.34
hsa-miR-29c-3p	miR-29b-3p (and other miRNAs w/seed AGCACCA)	1.27	TargetScan Human	High (predicted)	NMI	NMI	1.95E-02	-1.34
hsa-miR-29c-3p	miR-29b-3p (and other miRNAs w/seed AGCACCA)	1.27	TargetScan Human	Moderate (predicted)	PAPOLG	PAPOLG	1.72E-02	-1.22
hsa-miR-29c-3p	miR-29b-3p (and other miRNAs w/seed AGCACCA)	1.27	Ingenuity Expert Findings,TargetScan Human,miRecords	Experimentally Observed,Moderate (predicted)	PIK3R1	PIK3R1	2.60E-02	-1.21
hsa-miR-29c-3p	miR-29b-3p (and other miRNAs w/seed AGCACCA)	1.27	TargetScan Human	Moderate (predicted)	PIK3R3	PIK3R3	5.19E-03	-1.28
hsa-miR-29c-3p	miR-29b-3p (and other miRNAs w/seed AGCACCA)	1.27	TargetScan Human	Moderate (predicted)	PLEKHF2	PLEKHF2	2.57E-02	-1.24
hsa-miR-29c-3p	miR-29b-3p (and other miRNAs w/seed AGCACCA)	1.27	TargetScan Human	Moderate (predicted)	PPIP5K2	PPIP5K2	1.90E-02	-1.27
hsa-miR-29c-3p	miR-29b-3p (and other miRNAs w/seed AGCACCA)	1.27	TargetScan Human	Moderate (predicted)	PRR14L	PRR14L	1.38E-02	-1.22
hsa-miR-29c-3p	miR-29b-3p (and other miRNAs w/seed AGCACCA)	1.27	TargetScan Human	Moderate (predicted)	RAP1GDS1	RAP1GDS1	1.33E-02	-1.29
hsa-miR-29c-3p	miR-29b-3p (and other miRNAs w/seed AGCACCA)	1.27	TargetScan Human	High (predicted)	REV3L	REV3L	1.94E-02	-1.21
hsa-miR-29c-3p	miR-29b-3p (and other miRNAs w/seed AGCACCA)	1.27	TargetScan Human	High (predicted)	RGS1	RGS1	3.67E-02	-1.23
hsa-miR-29c-3p	miR-29b-3p (and other miRNAs w/seed AGCACCA)	1.27	TargetScan Human	Moderate (predicted)	RIT1	RIT1	1.44E-02	-1.21
hsa-miR-29c-3p	miR-29b-3p (and other miRNAs w/seed AGCACCA)	1.27	TargetScan Human	High (predicted)	SCML1	SCML1	3.03E-02	-1.33
hsa-miR-29c-3p	miR-29b-3p (and other miRNAs w/seed AGCACCA)	1.27	TargetScan Human	Moderate (predicted)	SERBP1	SERBP1	7.08E-04	-1.26
hsa-miR-29c-3p	miR-29b-3p (and other miRNAs w/seed AGCACCA)	1.27	miRecords	Experimentally Observed	SP1	SP1	2.88E-03	-1.20
hsa-miR-29c-3p	miR-29b-3p (and other miRNAs w/seed AGCACCA)	1.27	TargetScan Human	Moderate (predicted)	SPDL1	SPDL1	1.80E-02	-1.32
hsa-miR-29c-3p	miR-29b-3p (and other miRNAs w/seed AGCACCA)	1.27	TargetScan Human	Moderate (predicted)	STAG2	STAG2	1.89E-02	-1.20
hsa-miR-29c-3p	miR-29b-3p (and other miRNAs w/seed AGCACCA)	1.27	TargetScan Human	Moderate (predicted)	TIPRL	TIPRL	5.30E-03	-1.23
hsa-miR-29c-3p	miR-29b-3p (and other miRNAs w/seed AGCACCA)	1.27	TargetScan Human	Moderate (predicted)	TRAF5	TRAF5	1.82E-02	-1.22
hsa-miR-29c-3p	miR-29b-3p (and other miRNAs w/seed AGCACCA)	1.27	TargetScan Human	Moderate (predicted)	TRIM37	TRIM37	5.80E-03	-1.20
hsa-miR-29c-3p	miR-29b-3p (and other miRNAs w/seed AGCACCA)	1.27	TargetScan Human	Moderate (predicted)	UBA3	UBA3	2.50E-02	-1.21
hsa-miR-29c-3p	miR-29b-3p (and other miRNAs w/seed AGCACCA)	1.27	TargetScan Human	Moderate (predicted)	VANGL1	VANGL1	1.26E-02	-1.49
hsa-miR-29c-3p	miR-29b-3p (and other miRNAs w/seed AGCACCA)	1.27	TargetScan Human	Moderate (predicted)	VTA1	VTA1	1.61E-02	-1.24
hsa-miR-29c-3p	miR-29b-3p (and other miRNAs w/seed AGCACCA)	1.27	TargetScan Human	Moderate (predicted)	ZNF519	ZNF519	1.69E-03	-1.20
hsa-miR-29c-3p	miR-29b-3p (and other miRNAs w/seed AGCACCA)	1.27	TargetScan Human	Moderate (predicted)	ZNF614	ZNF614	1.85E-02	-1.21
hsa-miR-210-5p	miR-3070-5p (and other miRNAs w/seed GCCCCUG)	1.28	TargetScan Human	Moderate (predicted)	BNIP2	BNIP2	1.70E-02	-1.22
hsa-miR-210-5p	miR-3070-5p (and other miRNAs w/seed GCCCCUG)	1.28	TargetScan Human	Moderate (predicted)	C2orf49	C2orf49	8.35E-03	-1.21
hsa-miR-210-5p	miR-3070-5p (and other miRNAs w/seed GCCCCUG)	1.28	TargetScan Human	Moderate (predicted)	COTL1	COTL1	4.54E-02	-1.21
hsa-miR-210-5p	miR-3070-5p (and other miRNAs w/seed GCCCCUG)	1.28	TargetScan Human	Moderate (predicted)	EIF2S1	EIF2S1	2.69E-02	-1.21
hsa-miR-210-5p	miR-3070-5p (and other miRNAs w/seed GCCCCUG)	1.28	TargetScan Human	Moderate (predicted)	ERGIC2	ERGIC2	2.99E-02	-1.21
hsa-miR-210-5p	miR-3070-5p (and other miRNAs w/seed GCCCCUG)	1.28	TargetScan Human	Moderate (predicted)	FAM217B	FAM217B	3.45E-03	-1.26
hsa-miR-210-5p	miR-3070-5p (and other miRNAs w/seed GCCCCUG)	1.28	TargetScan Human	Moderate (predicted)	IKBIP	IKBIP	1.12E-02	-1.25
hsa-miR-210-5p	miR-3070-5p (and other miRNAs w/seed GCCCCUG)	1.28	TargetScan Human	Moderate (predicted)	OSBPL11	OSBPL11	2.82E-03	-1.26
hsa-miR-210-5p	miR-3070-5p (and other miRNAs w/seed GCCCCUG)	1.28	TargetScan Human	Moderate (predicted)	PIK3R3	PIK3R3	5.19E-03	-1.28
hsa-miR-210-5p	miR-3070-5p (and other miRNAs w/seed GCCCCUG)	1.28	TargetScan Human	Moderate (predicted)	RBBP9	RBBP9	9.79E-03	-1.23
hsa-miR-210-5p	miR-3070-5p (and other miRNAs w/seed GCCCCUG)	1.28	TargetScan Human	Moderate (predicted)	SKA1	SKA1	4.51E-02	-1.30
hsa-miR-210-5p	miR-3070-5p (and other miRNAs w/seed GCCCCUG)	1.28	TargetScan Human	Moderate (predicted)	TMEM135	TMEM135	7.02E-03	-1.23
hsa-miR-210-5p	miR-3070-5p (and other miRNAs w/seed GCCCCUG)	1.28	TargetScan Human	Moderate (predicted)	ZFP69B	ZFP69B	1.69E-02	-1.21
hsa-miR-210-5p	miR-3070-5p (and other miRNAs w/seed GCCCCUG)	1.28	TargetScan Human	Moderate (predicted)	ZNF641	ZNF641	8.80E-03	-1.22
hsa-miR-3127-5p	miR-3127-5p (miRNAs w/seed UCAGGGC)	-1.25	TargetScan Human	Moderate (predicted)	SPRR2D	SPRR2D	6.41E-03	1.35
hsa-miR-3141	miR-3141 (miRNAs w/seed AGGGCGG)	1.91	TargetScan Human	Moderate (predicted)	ANO10	ANO10	1.53E-02	-1.30
hsa-miR-3141	miR-3141 (miRNAs w/seed AGGGCGG)	1.91	TargetScan Human	Moderate (predicted)	CEPT1	CEPT1	6.78E-03	-1.27
hsa-miR-3141	miR-3141 (miRNAs w/seed AGGGCGG)	1.91	TargetScan Human	Moderate (predicted)	GRAMD3	GRAMD2B	6.29E-03	-1.36
hsa-miR-3141	miR-3141 (miRNAs w/seed AGGGCGG)	1.91	TargetScan Human	Moderate (predicted)	LILRB4	LILRB4	3.57E-02	-1.23
hsa-miR-3141	miR-3141 (miRNAs w/seed AGGGCGG)	1.91	TargetScan Human	Moderate (predicted)	RAP2A	RAP2A	3.39E-03	-1.23
hsa-miR-3196	miR-3180-3p (and other miRNAs w/seed GGGGCGG)	1.75	TargetScan Human	Moderate (predicted)	KPNA6	KPNA6	3.28E-02	-1.20
hsa-miR-3196	miR-3180-3p (and other miRNAs w/seed GGGGCGG)	1.75	TargetScan Human	Moderate (predicted)	MOCS2	MOCS2	3.61E-02	-1.22
hsa-miR-3196	miR-3180-3p (and other miRNAs w/seed GGGGCGG)	1.75	TargetScan Human	Moderate (predicted)	PBX2	PBX2	3.61E-02	-1.23
hsa-miR-3196	miR-3180-3p (and other miRNAs w/seed GGGGCGG)	1.75	TargetScan Human	Moderate (predicted)	SKA3	SKA3	2.44E-02	-1.24
hsa-miR-3196	miR-3180-3p (and other miRNAs w/seed GGGGCGG)	1.75	TargetScan Human	Moderate (predicted)	WARS2	WARS2	8.04E-03	-1.23
hsa-miR-3182	miR-3182 (miRNAs w/seed CUUCUGU)	1.41	TargetScan Human	High (predicted)	ANP32E	ANP32E	1.66E-02	-1.26
hsa-miR-3182	miR-3182 (miRNAs w/seed CUUCUGU)	1.41	TargetScan Human	Moderate (predicted)	CD2AP	CD2AP	1.20E-02	-1.27
hsa-miR-3182	miR-3182 (miRNAs w/seed CUUCUGU)	1.41	TargetScan Human	Moderate (predicted)	CHD1	CHD1	2.41E-02	-1.22
hsa-miR-3182	miR-3182 (miRNAs w/seed CUUCUGU)	1.41	TargetScan Human	Moderate (predicted)	CIR1	CIR1	1.77E-02	-1.27
hsa-miR-3182	miR-3182 (miRNAs w/seed CUUCUGU)	1.41	TargetScan Human	Moderate (predicted)	FAM49B	CYRIB	3.07E-02	-1.21
hsa-miR-3182	miR-3182 (miRNAs w/seed CUUCUGU)	1.41	TargetScan Human	Moderate (predicted)	DDIAS	DDIAS	4.77E-02	-1.22
hsa-miR-3182	miR-3182 (miRNAs w/seed CUUCUGU)	1.41	TargetScan Human	High (predicted)	EEF1E1	EEF1E1	2.32E-02	-1.25
hsa-miR-3182	miR-3182 (miRNAs w/seed CUUCUGU)	1.41	TargetScan Human	High (predicted)	GABPA	GABPA	1.43E-02	-1.22
hsa-miR-3182	miR-3182 (miRNAs w/seed CUUCUGU)	1.41	TargetScan Human	Moderate (predicted)	GCFC2	GCFC2	1.49E-02	-1.26
hsa-miR-3182	miR-3182 (miRNAs w/seed CUUCUGU)	1.41	TargetScan Human	Moderate (predicted)	HAUS2	HAUS2	1.03E-02	-1.21
hsa-miR-3182	miR-3182 (miRNAs w/seed CUUCUGU)	1.41	TargetScan Human	Moderate (predicted)	NDFIP2	NDFIP2	2.72E-02	-1.21
hsa-miR-3182	miR-3182 (miRNAs w/seed CUUCUGU)	1.41	TargetScan Human	High (predicted)	POC1B	POC1B	1.71E-02	-1.24
hsa-miR-3182	miR-3182 (miRNAs w/seed CUUCUGU)	1.41	TargetScan Human	Moderate (predicted)	PPP1R12A	PPP1R12A	2.74E-03	-1.22
hsa-miR-3182	miR-3182 (miRNAs w/seed CUUCUGU)	1.41	TargetScan Human	Moderate (predicted)	PRKACB	PRKACB	4.51E-02	-1.24
hsa-miR-3182	miR-3182 (miRNAs w/seed CUUCUGU)	1.41	TargetScan Human	Moderate (predicted)	RBM7	RBM7	2.83E-02	-1.33
hsa-miR-3182	miR-3182 (miRNAs w/seed CUUCUGU)	1.41	TargetScan Human	Moderate (predicted)	RRM2	RRM2	4.67E-02	-1.23
hsa-miR-3182	miR-3182 (miRNAs w/seed CUUCUGU)	1.41	TargetScan Human	Moderate (predicted)	SENP6	SENP6	7.96E-03	-1.25
hsa-miR-3182	miR-3182 (miRNAs w/seed CUUCUGU)	1.41	TargetScan Human	Moderate (predicted)	TANK	TANK	8.55E-03	-1.25
hsa-miR-3182	miR-3182 (miRNAs w/seed CUUCUGU)	1.41	TargetScan Human	Moderate (predicted)	FAM208A	TASOR	1.50E-02	-1.28
hsa-miR-3182	miR-3182 (miRNAs w/seed CUUCUGU)	1.41	TargetScan Human	Moderate (predicted)	TBL1XR1	TBL1XR1	4.12E-03	-1.29
hsa-miR-3182	miR-3182 (miRNAs w/seed CUUCUGU)	1.41	TargetScan Human	High (predicted)	CCDC132	VPS50	2.81E-02	-1.21
hsa-miR-3182	miR-3182 (miRNAs w/seed CUUCUGU)	1.41	TargetScan Human	High (predicted)	WDR3	WDR3	2.42E-02	-1.20
hsa-miR-3182	miR-3182 (miRNAs w/seed CUUCUGU)	1.41	TargetScan Human	Moderate (predicted)	ZDHHC20	ZDHHC20	4.36E-02	-1.21
hsa-miR-3651	miR-3651 (miRNAs w/seed AUAGCCC)	1.27	TargetScan Human	Moderate (predicted)	ADAM17	ADAM17	1.84E-02	-1.21
hsa-miR-3651	miR-3651 (miRNAs w/seed AUAGCCC)	1.27	TargetScan Human	High (predicted)	CCP110	CCP110	2.90E-02	-1.25
hsa-miR-3651	miR-3651 (miRNAs w/seed AUAGCCC)	1.27	TargetScan Human	High (predicted)	CDC5L	CDC5L	7.11E-03	-1.20
hsa-miR-3651	miR-3651 (miRNAs w/seed AUAGCCC)	1.27	TargetScan Human	High (predicted)	CDK8	CDK8	2.81E-03	-1.29
hsa-miR-3651	miR-3651 (miRNAs w/seed AUAGCCC)	1.27	TargetScan Human	Moderate (predicted)	COMMD3-BMI1	COMMD3-BMI1	2.28E-02	-1.22
hsa-miR-3651	miR-3651 (miRNAs w/seed AUAGCCC)	1.27	TargetScan Human	Moderate (predicted)	DDX46	DDX46	1.30E-02	-1.23
hsa-miR-3651	miR-3651 (miRNAs w/seed AUAGCCC)	1.27	TargetScan Human	High (predicted)	GNAI3	GNAI3	1.07E-02	-1.24
hsa-miR-3651	miR-3651 (miRNAs w/seed AUAGCCC)	1.27	TargetScan Human	High (predicted)	MSH2	MSH2	1.61E-02	-1.28
hsa-miR-3651	miR-3651 (miRNAs w/seed AUAGCCC)	1.27	TargetScan Human	Moderate (predicted)	MTBP	MTBP	2.47E-02	-1.25
hsa-miR-3651	miR-3651 (miRNAs w/seed AUAGCCC)	1.27	TargetScan Human	Moderate (predicted)	PBK	PBK	3.36E-02	-1.29
hsa-miR-3651	miR-3651 (miRNAs w/seed AUAGCCC)	1.27	TargetScan Human	Moderate (predicted)	PLA2G4C	PLA2G4C	8.53E-03	-1.25
hsa-miR-3651	miR-3651 (miRNAs w/seed AUAGCCC)	1.27	TargetScan Human	High (predicted)	PLEKHF2	PLEKHF2	2.57E-02	-1.24
hsa-miR-3651	miR-3651 (miRNAs w/seed AUAGCCC)	1.27	TargetScan Human	High (predicted)	RIT1	RIT1	1.44E-02	-1.21
hsa-miR-3651	miR-3651 (miRNAs w/seed AUAGCCC)	1.27	TargetScan Human	Moderate (predicted)	SPAG1	SPAG1	1.03E-02	-1.26
hsa-miR-3651	miR-3651 (miRNAs w/seed AUAGCCC)	1.27	TargetScan Human	Moderate (predicted)	TMEM135	TMEM135	7.02E-03	-1.23
hsa-miR-3651	miR-3651 (miRNAs w/seed AUAGCCC)	1.27	TargetScan Human	Moderate (predicted)	USP1	USP1	3.36E-02	-1.27
hsa-miR-3651	miR-3651 (miRNAs w/seed AUAGCCC)	1.27	TargetScan Human	Moderate (predicted)	VBP1	VBP1	3.01E-02	-1.22
hsa-miR-3651	miR-3651 (miRNAs w/seed AUAGCCC)	1.27	TargetScan Human	Moderate (predicted)	ZC2HC1A	ZC2HC1A	5.02E-03	-1.30
hsa-miR-3912-3p	miR-3912-3p (miRNAs w/seed AACGCAU)	1.25	TargetScan Human	Moderate (predicted)	BCLAF1	BCLAF1	2.03E-02	-1.22
hsa-miR-3912-3p	miR-3912-3p (miRNAs w/seed AACGCAU)	1.25	TargetScan Human	Moderate (predicted)	CAPZA2	CAPZA2	1.65E-02	-1.22
hsa-miR-3912-3p	miR-3912-3p (miRNAs w/seed AACGCAU)	1.25	TargetScan Human	Moderate (predicted)	HSPA4	HSPA4	1.34E-02	-1.28
hsa-miR-3912-3p	miR-3912-3p (miRNAs w/seed AACGCAU)	1.25	TargetScan Human	Moderate (predicted)	LYSMD3	LYSMD3	4.06E-02	-1.21
hsa-miR-3912-3p	miR-3912-3p (miRNAs w/seed AACGCAU)	1.25	TargetScan Human	Moderate (predicted)	PLEKHF2	PLEKHF2	2.57E-02	-1.24

hsa-miR-3912-3p	miR-3912-3p (miRNAs w/seed AACGCAU)	1.25	TargetScan Human	Moderate (predicted)	RSF1	RSF1	2.87E-02	-1.23
hsa-miR-3912-3p	miR-3912-3p (miRNAs w/seed AACGCAU)	1.25	TargetScan Human	Moderate (predicted)	SACS	SACS	3.73E-02	-1.20
hsa-miR-3912-3p	miR-3912-3p (miRNAs w/seed AACGCAU)	1.25	TargetScan Human	Moderate (predicted)	SERBP1	SERBP1	7.08E-04	-1.26
hsa-miR-3912-3p	miR-3912-3p (miRNAs w/seed AACGCAU)	1.25	TargetScan Human	High (predicted)	TRMT10C	TRMT10C	1.72E-02	-1.26
hsa-miR-3912-3p	miR-3912-3p (miRNAs w/seed AACGCAU)	1.25	TargetScan Human	Moderate (predicted)	ZNF721	ZNF721	1.20E-02	-1.23
hsa-miR-3960	miR-3960 (and other miRNAs w/seed GCGGCGG)	2.08	TargetScan Human	Moderate (predicted)	COTL1	COTL1	4.54E-02	-1.21
hsa-miR-4284	miR-4284 (miRNAs w/seed GGCUCAC)	1.21	TargetScan Human	Moderate (predicted)	ANO10	ANO10	1.53E-02	-1.30
hsa-miR-4284	miR-4284 (miRNAs w/seed GGCUCAC)	1.21	TargetScan Human	Moderate (predicted)	PPID	PPID	7.44E-04	-1.32
hsa-miR-4284	miR-4284 (miRNAs w/seed GGCUCAC)	1.21	TargetScan Human	Moderate (predicted)	PRIM2	PRIM2	1.12E-02	-1.31
hsa-miR-4284	miR-4284 (miRNAs w/seed GGCUCAC)	1.21	TargetScan Human	Moderate (predicted)	RNF125	RNF125	3.34E-02	-1.30
hsa-miR-4284	miR-4284 (miRNAs w/seed GGCUCAC)	1.21	TargetScan Human	Moderate (predicted)	RRM2	RRM2	4.67E-02	-1.23
hsa-miR-4284	miR-4284 (miRNAs w/seed GGCUCAC)	1.21	TargetScan Human	Moderate (predicted)	RRP36	RRP36	8.66E-03	-1.21
hsa-miR-4284	miR-4284 (miRNAs w/seed GGCUCAC)	1.21	TargetScan Human	High (predicted)	SGOL1	SGO1	2.11E-02	-1.25
hsa-miR-4284	miR-4284 (miRNAs w/seed GGCUCAC)	1.21	TargetScan Human	Moderate (predicted)	SLAMF6	SLAMF6	1.72E-02	-1.24
hsa-miR-4284	miR-4284 (miRNAs w/seed GGCUCAC)	1.21	TargetScan Human	Moderate (predicted)	TP53	TP53	1.54E-02	-1.21
hsa-miR-4284	miR-4284 (miRNAs w/seed GGCUCAC)	1.21	TargetScan Human	Moderate (predicted)	TRAPPC2	TRAPPC2	2.36E-02	-1.37
hsa-miR-4284	miR-4284 (miRNAs w/seed GGCUCAC)	1.21	TargetScan Human	Moderate (predicted)	UGDH	UGDH	3.92E-02	-1.25
hsa-miR-4449	miR-4449 (miRNAs w/seed GUCCCGG)	1.41	TargetScan Human	High (predicted)	CDKN3	CDKN3	4.45E-02	-1.25
hsa-miR-4449	miR-4449 (miRNAs w/seed GUCCCGG)	1.41	TargetScan Human	Moderate (predicted)	MFAP3	MFAP3	4.12E-02	-1.22
hsa-miR-4449	miR-4449 (miRNAs w/seed GUCCCGG)	1.41	TargetScan Human	High (predicted)	NCAPH	NCAPH	4.15E-02	-1.24
hsa-miR-4449	miR-4449 (miRNAs w/seed GUCCCGG)	1.41	TargetScan Human	Moderate (predicted)	NCF4	NCF4	1.97E-02	-1.28
hsa-miR-4449	miR-4449 (miRNAs w/seed GUCCCGG)	1.41	TargetScan Human	Moderate (predicted)	RRM2	RRM2	4.67E-02	-1.23
hsa-miR-4485-3p	miR-4485-3p (miRNAs w/seed AACGGCC)	1.36	TargetScan Human	Moderate (predicted)	GMFB	GMFB	3.70E-02	-1.21
hsa-miR-4485-3p	miR-4485-3p (miRNAs w/seed AACGGCC)	1.36	TargetScan Human	Moderate (predicted)	NMD3	NMD3	4.09E-03	-1.23
hsa-miR-4485-3p	miR-4485-3p (miRNAs w/seed AACGGCC)	1.36	TargetScan Human	Moderate (predicted)	PHF6	PHF6	6.76E-03	-1.27
hsa-miR-4485-3p	miR-4485-3p (miRNAs w/seed AACGGCC)	1.36	TargetScan Human	Moderate (predicted)	SREK1IP1	SREK1IP1	8.62E-03	-1.24
hsa-miR-4485-3p	miR-4485-3p (miRNAs w/seed AACGGCC)	1.36	TargetScan Human	Moderate (predicted)	VANGL1	VANGL1	1.26E-02	-1.49
hsa-miR-4485-3p	miR-4485-3p (miRNAs w/seed AACGGCC)	1.36	TargetScan Human	Moderate (predicted)	XRN1	XRN1	1.84E-02	-1.21
hsa-miR-4485-3p	miR-4485-3p (miRNAs w/seed AACGGCC)	1.36	TargetScan Human	Moderate (predicted)	ZFP69B	ZFP69B	1.69E-02	-1.21
hsa-miR-4485-5p	miR-4485-5p (miRNAs w/seed CCGCCUG)	1.28	TargetScan Human	Moderate (predicted)	FAM217B	FAM217B	3.45E-03	-1.26
hsa-miR-4485-5p	miR-4485-5p (miRNAs w/seed CCGCCUG)	1.28	TargetScan Human	Moderate (predicted)	FAM98A	FAM98A	1.91E-02	-1.22
hsa-miR-4485-5p	miR-4485-5p (miRNAs w/seed CCGCCUG)	1.28	TargetScan Human	Moderate (predicted)	HOOK1	HOOK1	1.23E-02	-1.36
hsa-miR-4485-5p	miR-4485-5p (miRNAs w/seed CCGCCUG)	1.28	TargetScan Human	Moderate (predicted)	NOB1	NOB1	3.33E-02	-1.23
hsa-miR-4485-5p	miR-4485-5p (miRNAs w/seed CCGCCUG)	1.28	TargetScan Human	Moderate (predicted)	POLR3F	POLR3F	4.48E-03	-1.23
hsa-miR-4485-5p	miR-4485-5p (miRNAs w/seed CCGCCUG)	1.28	TargetScan Human	Moderate (predicted)	RNF125	RNF125	3.34E-02	-1.30
hsa-miR-4485-5p	miR-4485-5p (miRNAs w/seed CCGCCUG)	1.28	TargetScan Human	Moderate (predicted)	RRM2	RRM2	4.67E-02	-1.23
hsa-miR-4485-5p	miR-4485-5p (miRNAs w/seed CCGCCUG)	1.28	TargetScan Human	High (predicted)	RRP36	RRP36	8.66E-03	-1.21
hsa-miR-4485-5p	miR-4485-5p (miRNAs w/seed CCGCCUG)	1.28	TargetScan Human	Moderate (predicted)	SGOL1	SGO1	2.11E-02	-1.25
hsa-miR-4485-5p	miR-4485-5p (miRNAs w/seed CCGCCUG)	1.28	TargetScan Human	Moderate (predicted)	UTP6	UTP6	6.72E-03	-1.21
hsa-miR-4485-5p	miR-4485-5p (miRNAs w/seed CCGCCUG)	1.28	TargetScan Human	High (predicted)	ZNF557	ZNF557	1.35E-02	-1.26
hsa-miR-4497	miR-4497 (miRNAs w/seed UCCGGGA)	1.56	TargetScan Human	Moderate (predicted)	APPBP2	APPBP2	2.57E-03	-1.24
hsa-miR-4497	miR-4497 (miRNAs w/seed UCCGGGA)	1.56	TargetScan Human	Moderate (predicted)	FOPNL	CEP20	4.87E-02	-1.20
hsa-miR-4497	miR-4497 (miRNAs w/seed UCCGGGA)	1.56	TargetScan Human	Moderate (predicted)	PIK3R1	PIK3R1	2.60E-02	-1.21
hsa-miR-4532	miR-4532 (miRNAs w/seed CCCGGGG)	1.80	TargetScan Human	Moderate (predicted)	COQ10B	COQ10B	2.06E-02	-1.21
hsa-miR-4532	miR-4532 (miRNAs w/seed CCCGGGG)	1.80	TargetScan Human	High (predicted)	COTL1	COTL1	4.54E-02	-1.21
hsa-miR-4532	miR-4532 (miRNAs w/seed CCCGGGG)	1.80	TargetScan Human	Moderate (predicted)	CPT1A	CPT1A	6.74E-03	-1.24
hsa-miR-4532	miR-4532 (miRNAs w/seed CCCGGGG)	1.80	TargetScan Human	Moderate (predicted)	MFAP3	MFAP3	4.12E-02	-1.22
hsa-miR-4532	miR-4532 (miRNAs w/seed CCCGGGG)	1.80	TargetScan Human	Moderate (predicted)	RRM2	RRM2	4.67E-02	-1.23
hsa-miR-4532	miR-4532 (miRNAs w/seed CCCGGGG)	1.80	TargetScan Human	High (predicted)	SP1	SP1	2.88E-03	-1.20
hsa-miR-4532	miR-4532 (miRNAs w/seed CCCGGGG)	1.80	TargetScan Human	Moderate (predicted)	SYNE2	SYNE2	3.43E-02	-1.23
hsa-miR-4532	miR-4532 (miRNAs w/seed CCCGGGG)	1.80	TargetScan Human	Moderate (predicted)	TLR10	TLR10	1.86E-02	-1.25
hsa-miR-4532	miR-4532 (miRNAs w/seed CCCGGGG)	1.80	TargetScan Human	Moderate (predicted)	ZNF143	ZNF143	1.12E-03	-1.27
hsa-miR-4532	miR-4532 (miRNAs w/seed CCCGGGG)	1.80	TargetScan Human	Moderate (predicted)	ZNF430	ZNF430	3.43E-02	-1.24
hsa-miR-505-3p	miR-505-3p (miRNAs w/seed GUCAACA)	1.27	TargetScan Human	Moderate (predicted)	ARHGAP19	ARHGAP19	4.22E-03	-1.22
hsa-miR-505-3p	miR-505-3p (miRNAs w/seed GUCAACA)	1.27	TargetScan Human	Moderate (predicted)	CCDC125	CCDC125	6.95E-03	-1.21
hsa-miR-505-3p	miR-505-3p (miRNAs w/seed GUCAACA)	1.27	TargetScan Human	High (predicted)	CCR6	CCR6	3.13E-02	-1.37
hsa-miR-505-3p	miR-505-3p (miRNAs w/seed GUCAACA)	1.27	TargetScan Human	Moderate (predicted)	CEP97	CEP97	2.66E-02	-1.23
hsa-miR-505-3p	miR-505-3p (miRNAs w/seed GUCAACA)	1.27	TargetScan Human	Moderate (predicted)	CEPT1	CEPT1	6.78E-03	-1.27
hsa-miR-505-3p	miR-505-3p (miRNAs w/seed GUCAACA)	1.27	TargetScan Human	Moderate (predicted)	DDIAS	DDIAS	4.77E-02	-1.22
hsa-miR-505-3p	miR-505-3p (miRNAs w/seed GUCAACA)	1.27	TargetScan Human	Moderate (predicted)	E2F5	E2F5	3.77E-03	-1.27
hsa-miR-505-3p	miR-505-3p (miRNAs w/seed GUCAACA)	1.27	TargetScan Human	Moderate (predicted)	FCF1	FCF1	9.68E-03	-1.31
hsa-miR-505-3p	miR-505-3p (miRNAs w/seed GUCAACA)	1.27	TargetScan Human	Moderate (predicted)	FKTN	FKTN	2.83E-02	-1.24
hsa-miR-505-3p	miR-505-3p (miRNAs w/seed GUCAACA)	1.27	TargetScan Human	Moderate (predicted)	GMFB	GMFB	3.70E-02	-1.21
hsa-miR-505-3p	miR-505-3p (miRNAs w/seed GUCAACA)	1.27	TargetScan Human	High (predicted)	GPR174	GPR174	3.25E-02	-1.24
hsa-miR-505-3p	miR-505-3p (miRNAs w/seed GUCAACA)	1.27	TargetScan Human	Moderate (predicted)	HOOK1	HOOK1	1.23E-02	-1.36
hsa-miR-505-3p	miR-505-3p (miRNAs w/seed GUCAACA)	1.27	TargetScan Human	Moderate (predicted)	IDI1	IDI1	1.62E-02	-1.22
hsa-miR-505-3p	miR-505-3p (miRNAs w/seed GUCAACA)	1.27	TargetScan Human	Moderate (predicted)	IL6ST	IL6ST	4.14E-02	-1.22
hsa-miR-505-3p	miR-505-3p (miRNAs w/seed GUCAACA)	1.27	TargetScan Human	High (predicted)	LIMS1	LIMS1	6.81E-03	-1.34
hsa-miR-505-3p	miR-505-3p (miRNAs w/seed GUCAACA)	1.27	TargetScan Human	Moderate (predicted)	LIN7C	LIN7C	1.55E-02	-1.21
hsa-miR-505-3p	miR-505-3p (miRNAs w/seed GUCAACA)	1.27	TargetScan Human	High (predicted)	LRRCC1	LRRCC1	2.18E-02	-1.29
hsa-miR-505-3p	miR-505-3p (miRNAs w/seed GUCAACA)	1.27	TargetScan Human	Moderate (predicted)	MED17	MED17	3.92E-03	-1.22
hsa-miR-505-3p	miR-505-3p (miRNAs w/seed GUCAACA)	1.27	TargetScan Human	Moderate (predicted)	MELK	MELK	2.94E-02	-1.22
hsa-miR-505-3p	miR-505-3p (miRNAs w/seed GUCAACA)	1.27	TargetScan Human	Moderate (predicted)	MIS18BP1	MIS18BP1	8.24E-03	-1.28
hsa-miR-505-3p	miR-505-3p (miRNAs w/seed GUCAACA)	1.27	TargetScan Human	High (predicted)	MSANTD4	MSANTD4	1.53E-02	-1.21
hsa-miR-505-3p	miR-505-3p (miRNAs w/seed GUCAACA)	1.27	TargetScan Human	High (predicted)	PBX3	PBX3	2.21E-02	-1.21
hsa-miR-505-3p	miR-505-3p (miRNAs w/seed GUCAACA)	1.27	TargetScan Human	Moderate (predicted)	PIP5K1A	PIP5K1A	4.91E-02	-1.20
hsa-miR-505-3p	miR-505-3p (miRNAs w/seed GUCAACA)	1.27	TargetScan Human	Moderate (predicted)	PLK4	PLK4	1.06E-02	-1.30
hsa-miR-505-3p	miR-505-3p (miRNAs w/seed GUCAACA)	1.27	TargetScan Human	Moderate (predicted)	RIOK1	RIOK1	1.03E-02	-1.20
hsa-miR-505-3p	miR-505-3p (miRNAs w/seed GUCAACA)	1.27	TargetScan Human	Moderate (predicted)	RPS6KA6	RPS6KA6	4.78E-02	-1.25
hsa-miR-505-3p	miR-505-3p (miRNAs w/seed GUCAACA)	1.27	TargetScan Human	Moderate (predicted)	SCAF11	SCAF11	9.28E-03	-1.21
hsa-miR-505-3p	miR-505-3p (miRNAs w/seed GUCAACA)	1.27	TargetScan Human	Moderate (predicted)	SEPT7	SEPTIN7	4.53E-03	-1.32
hsa-miR-505-3p	miR-505-3p (miRNAs w/seed GUCAACA)	1.27	TargetScan Human	Moderate (predicted)	SKA3	SKA3	2.44E-02	-1.24
hsa-miR-505-3p	miR-505-3p (miRNAs w/seed GUCAACA)	1.27	TargetScan Human	High (predicted)	SYAP1	SYAP1	2.29E-02	-1.26
hsa-miR-505-3p	miR-505-3p (miRNAs w/seed GUCAACA)	1.27	TargetScan Human	Moderate (predicted)	TIGD2	TIGD2	1.15E-02	-1.23
hsa-miR-505-3p	miR-505-3p (miRNAs w/seed GUCAACA)	1.27	TargetScan Human	Moderate (predicted)	UPF3B	UPF3B	2.54E-02	-1.20
hsa-miR-505-3p	miR-505-3p (miRNAs w/seed GUCAACA)	1.27	TargetScan Human	Moderate (predicted)	VBP1	VBP1	3.01E-02	-1.22
hsa-miR-505-3p	miR-505-3p (miRNAs w/seed GUCAACA)	1.27	TargetScan Human	Moderate (predicted)	ZNF33B	ZNF33B	3.20E-02	-1.24
hsa-miR-505-3p	miR-505-3p (miRNAs w/seed GUCAACA)	1.27	TargetScan Human	Moderate (predicted)	ZNF525	ZNF525	3.84E-02	-1.26
hsa-miR-505-3p	miR-505-3p (miRNAs w/seed GUCAACA)	1.27	TargetScan Human	Moderate (predicted)	ZNF614	ZNF614	1.85E-02	-1.21
hsa-miR-505-3p	miR-505-3p (miRNAs w/seed GUCAACA)	1.27	TargetScan Human	High (predicted)	ZNF675	ZNF675	3.08E-02	-1.36
hsa-miR-505-3p	miR-505-3p (miRNAs w/seed GUCAACA)	1.27	TargetScan Human	Moderate (predicted)	ZNF91	ZNF91	2.19E-02	-1.46
hsa-miR-5100	miR-5100 (miRNAs w/seed UCAGAUC)	1.37	TargetScan Human	Moderate (predicted)	CCDC186	CCDC186	1.29E-02	-1.27
hsa-miR-5100	miR-5100 (miRNAs w/seed UCAGAUC)	1.37	TargetScan Human	Moderate (predicted)	CCDC91	CCDC91	1.49E-02	-1.21
hsa-miR-5100	miR-5100 (miRNAs w/seed UCAGAUC)	1.37	TargetScan Human	Moderate (predicted)	CHRNA5	CHRNA5	4.02E-02	-1.25
hsa-miR-5100	miR-5100 (miRNAs w/seed UCAGAUC)	1.37	TargetScan Human	Moderate (predicted)	DEK	DEK	1.42E-02	-1.26
hsa-miR-5100	miR-5100 (miRNAs w/seed UCAGAUC)	1.37	TargetScan Human	Moderate (predicted)	FASTKD3	FASTKD3	1.66E-02	-1.22
hsa-miR-5100	miR-5100 (miRNAs w/seed UCAGAUC)	1.37	TargetScan Human	Moderate (predicted)	FRYL	FRYL	2.48E-02	-1.23
hsa-miR-5100	miR-5100 (miRNAs w/seed UCAGAUC)	1.37	TargetScan Human	Moderate (predicted)	C11orf73	HIKESHI	2.98E-02	-1.21
hsa-miR-5100	miR-5100 (miRNAs w/seed UCAGAUC)	1.37	TargetScan Human	High (predicted)	MFAP3	MFAP3	4.12E-02	-1.22
hsa-miR-5100	miR-5100 (miRNAs w/seed UCAGAUC)	1.37	TargetScan Human	Moderate (predicted)	NCF4	NCF4	1.97E-02	-1.28
hsa-miR-5100	miR-5100 (miRNAs w/seed UCAGAUC)	1.37	TargetScan Human	Moderate (predicted)	OSBPL8	OSBPL8	8.73E-03	-1.29
hsa-miR-5100	miR-5100 (miRNAs w/seed UCAGAUC)	1.37	TargetScan Human	Moderate (predicted)	PIK3R1	PIK3R1	2.60E-02	-1.21
hsa-miR-5100	miR-5100 (miRNAs w/seed UCAGAUC)	1.37	TargetScan Human	Moderate (predicted)	RBM7	RBM7	2.83E-02	-1.33
hsa-miR-5100	miR-5100 (miRNAs w/seed UCAGAUC)	1.37	TargetScan Human	Moderate (predicted)	SCRN3	SCRN3	3.70E-02	-1.23
hsa-miR-5100	miR-5100 (miRNAs w/seed UCAGAUC)	1.37	TargetScan Human	Moderate (predicted)	SPDL1	SPDL1	1.80E-02	-1.32
hsa-miR-5100	miR-5100 (miRNAs w/seed UCAGAUC)	1.37	TargetScan Human	Moderate (predicted)	ZFP90	ZFP90	3.01E-02	-1.20
hsa-miR-6089	miR-6089 (miRNAs w/seed GAGGCCG)	2.74	TargetScan Human	Moderate (predicted)	COTL1	COTL1	4.54E-02	-1.21
hsa-miR-6089	miR-6089 (miRNAs w/seed GAGGCCG)	2.74	TargetScan Human	Moderate (predicted)	DYNLT3	DYNLT3	4.97E-02	-1.24
hsa-miR-6089	miR-6089 (miRNAs w/seed GAGGCCG)	2.74	TargetScan Human	Moderate (predicted)	GJB2	GJB2	1.38E-02	-1.26
hsa-miR-6089	miR-6089 (miRNAs w/seed GAGGCCG)	2.74	TargetScan Human	High (predicted)	GMFB	GMFB	3.70E-02	-1.21
hsa-miR-6089	miR-6089 (miRNAs w/seed GAGGCCG)	2.74	TargetScan Human	Moderate (predicted)	REEP3	REEP3	2.93E-02	-1.28
hsa-miR-6089	miR-6089 (miRNAs w/seed GAGGCCG)	2.74	TargetScan Human	Moderate (predicted)	TOR1AIP2	TOR1AIP2	3.26E-02	-1.29
hsa-miR-6089	miR-6089 (miRNAs w/seed GAGGCCG)	2.74	TargetScan Human	Moderate (predicted)	TPMT	TPMT	2.54E-02	-1.45
hsa-miR-664a-3p	miR-664-3p (and other miRNAs w/seed AUUCAUU)	1.29	TargetScan Human	Moderate (predicted)	AGL	AGL	2.62E-02	-1.23
hsa-miR-664a-3p	miR-664-3p (and other miRNAs w/seed AUUCAUU)	1.29	TargetScan Human	Moderate (predicted)	CSNK1G3	CSNK1G3	2.51E-02	-1.22
hsa-miR-664a-3p	miR-664-3p (and other miRNAs w/seed AUUCAUU)	1.29	TargetScan Human	Moderate (predicted)	DIMT1	DIMT1	7.36E-03	-1.23
hsa-miR-664a-3p	miR-664-3p (and other miRNAs w/seed AUUCAUU)	1.29	TargetScan Human	Moderate (predicted)	FASTKD3	FASTKD3	1.66E-02	-1.22
hsa-miR-664a-3p	miR-664-3p (and other miRNAs w/seed AUUCAUU)	1.29	TargetScan Human	Moderate (predicted)	GNPNAT1	GNPNAT1	8.00E-03	-1.25
hsa-miR-664a-3p	miR-664-3p (and other miRNAs w/seed AUUCAUU)	1.29	TargetScan Human	Moderate (predicted)	C11orf73	HIKESHI	2.98E-02	-1.21
hsa-miR-664a-3p	miR-664-3p (and other miRNAs w/seed AUUCAUU)	1.29	TargetScan Human	Moderate (predicted)	IDE	IDE	1.04E-02	-1.26
hsa-miR-664a-3p	miR-664-3p (and other miRNAs w/seed AUUCAUU)	1.29	TargetScan Human	Moderate (predicted)	LYRM7	LYRM7	4.89E-03	-1.43
hsa-miR-664a-3p	miR-664-3p (and other miRNAs w/seed AUUCAUU)	1.29	TargetScan Human	Moderate (predicted)	MARCH7	MARCHF7	3.61E-03	-1.22
hsa-miR-664a-3p	miR-664-3p (and other miRNAs w/seed AUUCAUU)	1.29	TargetScan Human	High (predicted)	MGST1	MGST1	1.50E-02	-1.37
hsa-miR-664a-3p	miR-664-3p (and other miRNAs w/seed AUUCAUU)	1.29	TargetScan Human	Moderate (predicted)	MSMO1	MSMO1	1.62E-02	-1.26
hsa-miR-664a-3p	miR-664-3p (and other miRNAs w/seed AUUCAUU)	1.29	TargetScan Human	High (predicted)	MTERF3	MTERF3	1.48E-02	-1.22
hsa-miR-664a-3p	miR-664-3p (and other miRNAs w/seed AUUCAUU)	1.29	TargetScan Human	High (predicted)	NMI	NMI	1.95E-02	-1.34
hsa-miR-664a-3p	miR-664-3p (and other miRNAs w/seed AUUCAUU)	1.29	TargetScan Human	Moderate (predicted)	PIK3R4	PIK3R4	7.05E-03	-1.22
hsa-miR-664a-3p	miR-664-3p (and other miRNAs w/seed AUUCAUU)	1.29	TargetScan Human	Moderate (predicted)	PTPN22	PTPN22	2.48E-02	-1.26
hsa-miR-664a-3p	miR-664-3p (and other miRNAs w/seed AUUCAUU)	1.29	TargetScan Human	Moderate (predicted)	RBM41	RBM41	3.23E-03	-1.21
hsa-miR-664a-3p	miR-664-3p (and other miRNAs w/seed AUUCAUU)	1.29	TargetScan Human	Moderate (predicted)	SQLE	SQLE	2.31E-02	-1.21
hsa-miR-664a-3p	miR-664-3p (and other miRNAs w/seed AUUCAUU)	1.29	TargetScan Human	High (predicted)	TRAPPC13	TRAPPC13	1.80E-02	-1.22

hsa-miR-664a-3p	miR-664-3p (and other miRNAs w/seed AUUCAUU)	1.29	TargetScan Human	Moderate (predicted)	TSNAX	TSNAX	3.23E-02	-1.23
hsa-miR-664a-3p	miR-664-3p (and other miRNAs w/seed AUUCAUU)	1.29	TargetScan Human	Moderate (predicted)	TTC33	TTC33	1.32E-02	-1.22
hsa-miR-664a-3p	miR-664-3p (and other miRNAs w/seed AUUCAUU)	1.29	TargetScan Human	Moderate (predicted)	UBE2V2	UBE2V2	4.99E-03	-1.25
hsa-miR-8485	miR-669c-3p (and other miRNAs w/seed ACACACA)	-1.30	TargetScan Human	High (predicted)	SPINT3	SPINT3	3.15E-03	1.33
hsa-miR-671-3p	miR-671-3p (and other miRNAs w/seed CCGGUUC)	1.21	TargetScan Human	High (predicted)	BRCA1	BRCA1	3.82E-02	-1.23
hsa-miR-671-3p	miR-671-3p (and other miRNAs w/seed CCGGUUC)	1.21	TargetScan Human	High (predicted)	PIP5K1A	PIP5K1A	4.91E-02	-1.20
hsa-miR-671-3p	miR-671-3p (and other miRNAs w/seed CCGGUUC)	1.21	TargetScan Human	Moderate (predicted)	USP25	USP25	8.62E-03	-1.22
hsa-miR-4492	miR-762 (and other miRNAs w/seed GGGCUGG)	2.36	TargetScan Human	Moderate (predicted)	AK2	AK2	9.41E-03	-1.24
hsa-miR-4492	miR-762 (and other miRNAs w/seed GGGCUGG)	2.36	TargetScan Human	Moderate (predicted)	CDCA8	CDCA8	3.23E-02	-1.21
hsa-miR-4492	miR-762 (and other miRNAs w/seed GGGCUGG)	2.36	TargetScan Human	High (predicted)	CDCP1	CDCP1	3.84E-02	-1.24
hsa-miR-4492	miR-762 (and other miRNAs w/seed GGGCUGG)	2.36	TargetScan Human	High (predicted)	COTL1	COTL1	4.54E-02	-1.21
hsa-miR-4492	miR-762 (and other miRNAs w/seed GGGCUGG)	2.36	TargetScan Human	High (predicted)	DDIAS	DDIAS	4.77E-02	-1.22
hsa-miR-4492	miR-762 (and other miRNAs w/seed GGGCUGG)	2.36	TargetScan Human	Moderate (predicted)	DDX10	DDX10	1.57E-02	-1.21
hsa-miR-4492	miR-762 (and other miRNAs w/seed GGGCUGG)	2.36	TargetScan Human	Moderate (predicted)	DGKE	DGKE	2.75E-02	-1.21
hsa-miR-4492	miR-762 (and other miRNAs w/seed GGGCUGG)	2.36	TargetScan Human	Moderate (predicted)	DOCK5	DOCK5	2.88E-02	-1.35
hsa-miR-4492	miR-762 (and other miRNAs w/seed GGGCUGG)	2.36	TargetScan Human	High (predicted)	EPB41L2	EPB41L2	1.20E-02	-1.28
hsa-miR-4492	miR-762 (and other miRNAs w/seed GGGCUGG)	2.36	TargetScan Human	High (predicted)	FCF1	FCF1	9.68E-03	-1.31
hsa-miR-4492	miR-762 (and other miRNAs w/seed GGGCUGG)	2.36	TargetScan Human	Moderate (predicted)	GRAMD3	GRAMD2B	6.29E-03	-1.36
hsa-miR-4492	miR-762 (and other miRNAs w/seed GGGCUGG)	2.36	TargetScan Human	Moderate (predicted)	KIF23	KIF23	2.53E-02	-1.23
hsa-miR-4492	miR-762 (and other miRNAs w/seed GGGCUGG)	2.36	TargetScan Human	Moderate (predicted)	KIFAP3	KIFAP3	1.28E-02	-1.24
hsa-miR-4492	miR-762 (and other miRNAs w/seed GGGCUGG)	2.36	TargetScan Human	High (predicted)	KPNA6	KPNA6	3.28E-02	-1.20
hsa-miR-4492	miR-762 (and other miRNAs w/seed GGGCUGG)	2.36	TargetScan Human	Moderate (predicted)	LILRB4	LILRB4	3.57E-02	-1.23
hsa-miR-4492	miR-762 (and other miRNAs w/seed GGGCUGG)	2.36	TargetScan Human	Moderate (predicted)	LYRM7	LYRM7	4.89E-03	-1.43
hsa-miR-4492	miR-762 (and other miRNAs w/seed GGGCUGG)	2.36	TargetScan Human	Moderate (predicted)	MOCS2	MOCS2	3.61E-02	-1.22
hsa-miR-4492	miR-762 (and other miRNAs w/seed GGGCUGG)	2.36	TargetScan Human	Moderate (predicted)	NCOA7	NCOA7	1.57E-03	-1.27
hsa-miR-4492	miR-762 (and other miRNAs w/seed GGGCUGG)	2.36	TargetScan Human	Moderate (predicted)	NUDT5	NUDT5	3.74E-02	-1.24
hsa-miR-4492	miR-762 (and other miRNAs w/seed GGGCUGG)	2.36	TargetScan Human	Moderate (predicted)	PIK3R3	PIK3R3	5.19E-03	-1.28
hsa-miR-4492	miR-762 (and other miRNAs w/seed GGGCUGG)	2.36	TargetScan Human	Moderate (predicted)	PYROXD1	PYROXD1	5.19E-03	-1.35
hsa-miR-4492	miR-762 (and other miRNAs w/seed GGGCUGG)	2.36	TargetScan Human	Moderate (predicted)	RRM2	RRM2	4.67E-02	-1.23
hsa-miR-4492	miR-762 (and other miRNAs w/seed GGGCUGG)	2.36	TargetScan Human	Moderate (predicted)	SGOL1	SGO1	2.11E-02	-1.25
hsa-miR-4492	miR-762 (and other miRNAs w/seed GGGCUGG)	2.36	TargetScan Human	High (predicted)	SLC35B4	SLC35B4	2.87E-02	-1.20
hsa-miR-4492	miR-762 (and other miRNAs w/seed GGGCUGG)	2.36	TargetScan Human	Moderate (predicted)	TGM2	TGM2	1.07E-02	-1.25
hsa-miR-4492	miR-762 (and other miRNAs w/seed GGGCUGG)	2.36	TargetScan Human	Moderate (predicted)	TMOD2	TMOD2	3.86E-02	-1.31
hsa-miR-4492	miR-762 (and other miRNAs w/seed GGGCUGG)	2.36	TargetScan Human	Moderate (predicted)	TOR1AIP2	TOR1AIP2	3.26E-02	-1.29
hsa-miR-4492	miR-762 (and other miRNAs w/seed GGGCUGG)	2.36	TargetScan Human	Moderate (predicted)	TTC1	TTC1	1.82E-02	-1.23
hsa-miR-4492	miR-762 (and other miRNAs w/seed GGGCUGG)	2.36	TargetScan Human	Moderate (predicted)	UTP11L	UTP11	1.36E-02	-1.25
hsa-miR-4492	miR-762 (and other miRNAs w/seed GGGCUGG)	2.36	TargetScan Human	Moderate (predicted)	VANGL1	VANGL1	1.26E-02	-1.49
hsa-miR-4492	miR-762 (and other miRNAs w/seed GGGCUGG)	2.36	TargetScan Human	Moderate (predicted)	WARS2	WARS2	8.04E-03	-1.23
hsa-miR-4492	miR-762 (and other miRNAs w/seed GGGCUGG)	2.36	TargetScan Human	Moderate (predicted)	ZFP69B	ZFP69B	1.69E-02	-1.21
hsa-miR-4492	miR-762 (and other miRNAs w/seed GGGCUGG)	2.36	TargetScan Human	Moderate (predicted)	ZFP90	ZFP90	3.01E-02	-1.20
hsa-miR-4492	miR-762 (and other miRNAs w/seed GGGCUGG)	2.36	TargetScan Human	Moderate (predicted)	ZNF429	ZNF429	3.12E-02	-1.27
hsa-miR-4492	miR-762 (and other miRNAs w/seed GGGCUGG)	2.36	TargetScan Human	Moderate (predicted)	ZNF561	ZNF561	1.47E-03	-1.25
hsa-miR-7704	miR-7704 (miRNAs w/seed GGGGUCG)	1.34	TargetScan Human	Moderate (predicted)	BRCA1	BRCA1	3.82E-02	-1.23
hsa-miR-7704	miR-7704 (miRNAs w/seed GGGGUCG)	1.34	TargetScan Human	Moderate (predicted)	CRYZ	CRYZ	8.14E-03	-1.30
hsa-miR-7704	miR-7704 (miRNAs w/seed GGGGUCG)	1.34	TargetScan Human	Moderate (predicted)	EIF2AK4	EIF2AK4	1.62E-02	-1.20
hsa-miR-7704	miR-7704 (miRNAs w/seed GGGGUCG)	1.34	TargetScan Human	Moderate (predicted)	GJB2	GJB2	1.38E-02	-1.26
hsa-miR-7704	miR-7704 (miRNAs w/seed GGGGUCG)	1.34	TargetScan Human	Moderate (predicted)	LILRB4	LILRB4	3.57E-02	-1.23
hsa-miR-7704	miR-7704 (miRNAs w/seed GGGGUCG)	1.34	TargetScan Human	Moderate (predicted)	LYSMD3	LYSMD3	4.06E-02	-1.21
hsa-miR-7704	miR-7704 (miRNAs w/seed GGGGUCG)	1.34	TargetScan Human	Moderate (predicted)	MCM8	MCM8	4.32E-02	-1.23
hsa-miR-7704	miR-7704 (miRNAs w/seed GGGGUCG)	1.34	TargetScan Human	High (predicted)	MGST1	MGST1	1.50E-02	-1.37
hsa-miR-7704	miR-7704 (miRNAs w/seed GGGGUCG)	1.34	TargetScan Human	Moderate (predicted)	MRS2	MRS2	2.00E-02	-1.20
hsa-miR-7704	miR-7704 (miRNAs w/seed GGGGUCG)	1.34	TargetScan Human	Moderate (predicted)	MX2	MX2	2.52E-02	-1.20
hsa-miR-7704	miR-7704 (miRNAs w/seed GGGGUCG)	1.34	TargetScan Human	Moderate (predicted)	PUS7L	PUS7L	1.04E-02	-1.21
hsa-miR-7704	miR-7704 (miRNAs w/seed GGGGUCG)	1.34	TargetScan Human	Moderate (predicted)	RBBP9	RBBP9	9.79E-03	-1.23
hsa-miR-7704	miR-7704 (miRNAs w/seed GGGGUCG)	1.34	TargetScan Human	Moderate (predicted)	RHOBTB3	RHOBTB3	3.12E-02	-1.24
hsa-miR-7704	miR-7704 (miRNAs w/seed GGGGUCG)	1.34	TargetScan Human	Moderate (predicted)	RRM2	RRM2	4.67E-02	-1.23
hsa-miR-7704	miR-7704 (miRNAs w/seed GGGGUCG)	1.34	TargetScan Human	High (predicted)	SUSD3	SUSD3	4.90E-03	-1.22
hsa-miR-7704	miR-7704 (miRNAs w/seed GGGGUCG)	1.34	TargetScan Human	Moderate (predicted)	TGM2	TGM2	1.07E-02	-1.25
hsa-miR-7704	miR-7704 (miRNAs w/seed GGGGUCG)	1.34	TargetScan Human	Moderate (predicted)	USP28	USP28	8.06E-03	-1.21
hsa-miR-7704	miR-7704 (miRNAs w/seed GGGGUCG)	1.34	TargetScan Human	Moderate (predicted)	ZNF561	ZNF561	1.47E-03	-1.25
hsa-miR-7704	miR-7704 (miRNAs w/seed GGGGUCG)	1.34	TargetScan Human	Moderate (predicted)	ZNF641	ZNF641	8.80E-03	-1.22
